# miR-378b Regulates Insulin Sensitivity by Targeting Insulin Receptor and p110α in Alcohol-Induced Hepatic Steatosis

**DOI:** 10.3389/fphar.2020.00717

**Published:** 2020-05-20

**Authors:** Yuan-yuan Li, Yu-juan Zhong, Qi Cheng, Ying-zhao Wang, Yuan-yuan Fan, Cheng-fang Yang, Zuheng Ma, Yong-wen Li, Li Li

**Affiliations:** ^1^College of Pharmacy, Guilin Medical University, Guilin, China; ^2^Department of Molecular Medicine and Surgery, Karolinska Institutet, Stockholm, Sweden; ^3^Center for Diabetic Systems Medicine, Guangxi Key Laboratory of Excellence, Guilin, China

**Keywords:** MiR-378b, insulin resistance, insulin receptor, p110α, alcoholic hepatic steatosis

## Abstract

Insulin resistance has been implicated in alcoholic liver disease. A previous study has shown that microRNAs (miRNAs) play a major role in the production, secretion, and function of insulin. MiRNAs are capable of repressing multiple target genes that in turn negatively regulate various physiological and pathological activities. However, current information on the biological function of miRNAs in insulin resistance is limited. The goal of the present study was to elucidate the role of miR-378b in alcohol-induced hepatic insulin resistance and its underlying mechanism. This study has observed that miR-378b is up-regulated in National Institute on Alcohol Abuse and Alcoholism (NIAAA) alcoholic mouse models as well as in ethanol-induced L-02 cells *in vitro*. Furthermore, miR-378b overexpression impaired the insulin signaling pathway, and inhibition of miR-378b improved insulin sensitivity *in vivo* and *in vitro*. A mechanistic study revealed that IR and p110α are direct targets of miR-378b. Together, these results suggest that miR-378b controls insulin sensitivity by targeting the insulin receptor (IR) as well as p110α and possibly play an inhibitory role in the development of insulin resistance, thereby providing insights into the development of novel diagnostic and treatment methods.

## Introduction

Alcoholic liver disease (ALD) is a common liver disorder with high global morbidity and mortality rates (Ramaiah et al., 2004). ALD pathogenesis consists of stages including steatosis, steatohepatitis, and fibrosis/cirrhosis (Chacko and Reinus, 2016). One of the most important organs for insulin action is the liver. Insulin resistance plays a pivotal role in the formation of ALD (Magdaleno et al., 2017; Chen et al., 2018). However, excessive alcohol uptake also increases the risk for insulin resistance, which is characterized by the inability of insulin-sensitive tissues to respond to insulin, resulting in various metabolic syndromes (Aberg et al., 2018; Tatsumi et al., 2018). Alcohol overconsumption may promote the pathogenesis of hepatic insulin resistance by inhibiting insulin signaling (Carr and Correnti, 2015). In addition, hepatic steatosis induced by alcohol leads to intracellular metabolic imbalance, can promote insulin resistance in liver cells (Samuel and Shulman, 2012; Perry et al., 2014). Thus, ALD and insulin resistance are intertwined in liver diseases.

Insulin receptor/insulin-receptor substrate (IR/IRS), combined with phosphoinositide 3-kinase (PI3K) heterodimers and serine-threonine protein kinase (Akt/PKB), are three well-defined and major nodes of the insulin signaling pathway (Taniguchi et al., 2006). PI3K comprises a regulatory subunit (p85) as well as a catalytic subunit (p110). In addition, p110α plays a central role in hepatic insulin/PI3K signaling and regulates glucose and lipid homeostasis (Liu et al., 2014). In normal physiological conditions, insulin binds to insulin receptors (IRs) on target tissues that in turn stimulates receptor autophosphorylation and internalization, thereby recruiting and activating insulin receptor substrate proteins 1 and 2 (IRS1/2). Then, IRS1/2 activates PI3K that converts phosphatidylinositol 4,5-bisphosphate (PIP2) into phosphatidylinositol (3,4,5)-trisphosphate (PIP3), which is situated on the cell membrane. Akt then binds to PIP3 at its pleckstrin homology domain, followed by its phosphorylation and activation (He et al., 2007; Samuel and Shulman, 2016; Parker et al., 2018). Elevated blood glucose stimulates islet β cells to secrete insulin and activates the PI3K/AKT signaling pathway in the liver, which play the following roles: 1) Inhibition of glycogen synthase kinase 3 (GSK3) increase glycogen synthase (GS) activity and thereby promote liver glycogen synthesis; 2) promote phosphorylation of forkhead box factor 1 (FoxO1) and reduces gluconeogenesis; 3) activate sterol regulatory element binding protein 1 (SREBP-1) and promote the synthesis of endogenous fatty acids (Perry et al., 2014; Carr and Correnti, 2015). Chronic ethanol consumption can disrupt IR, IRS1/2, PI3K, PIP3, and Akt expression or phosphorylation, which in turn decreases hepatocyte sensitivity to insulin and blocks the PI3K-Akt pathway, resulting in the decrease of glycogen synthesis, the increase of gluconeogenesis, the increase of fatty acid *de novo* synthesis, the increase of blood lipid. However, the body can only compensate for insulin resistance by increasing insulin secretion. A growing body of evidence shows that the decline of IR also contributes to the cause and development of IR and type 2 diabetes mellitus (T2DM). Knocking out IR leads to severe insulin resistance and impaired glucose homeostasis (Yang et al., 2015). Therefore, the expression levels of hepatic IR and p110α play an important role in insulin sensitivity of the whole body. However, the underlying mechanism of ethanol-induced downregulation of IR and p110α is still not fully understood.

MicroRNAs (miRNAs) pertain to endogenous non-coding RNAs that are 19–25 nucleotides in length and are conserved across species (Kim, 2005). Mature miRNAs have 2–8 relatively conserved nucleotides at its 5'-termini that are called “seed sequences” that interact with its target messenger RNA (mRNA) *via* specific base pairing. This miRNA interaction induces the degradation and/or disrupts the translation of its target gene mRNA, which is considered as a post-transcriptional regulatory mechanism of target gene expression (Bartel, 2004; Bartel, 2005). Although the specific target genes and biological functions of various miRNAs remain unclear, miRNAs are thought to play a central role in maintaining normal and pathological states. Growing evidence has shown that miRNAs are largely involved in the control of insulin signaling. For example, let-7 and miR-103/107 are mainly involved in controlling insulin signaling in peripheral tissues by targeting caveolin-1 and IR/IRS2, respectively (Frost and Olson, 2011; Trajkovski et al., 2011). Obesity caused by consumption of a high-fat diet induces insulin resistance in mice, which is associated with the upregulation of specific miRNAs, including miR-802, miR-29a, and miR-195 (Kornfeld et al., 2013; Yang et al., 2014a; Yang et al., 2014b). Human miR-378b, which has been localized to chromosome 3, belongs to the miR-378 family, and its seed sequence similar to miR-378a. An earlier study has revealed that miR-378b is upregulated during keratinocyte differentiation as well as promotes cell differentiation *via* NKX3.1 (Wang et al., 2015). In addition, the upregulation of miR-378b leads to the obstruction of the insulin signaling pathway and glucose metabolism (Liu et al., 2014). Unfortunately, the role of miR-378b in ethanol-induced liver insulin sensitivity remains unclear.

Numerous studies have shown that alcohol can cause alcoholic liver damage by activating oxidative stress, inhibiting liver fat-acid metabolism, and increasing liver lipid synthesis (Li et al., 2017; Li et al., 2018; Yang C. et al., 2018; Yang C. F. et al., 2018). Studies have also revealed that alcohol reduces hepatic glycogen synthesis and increases gluconeogenesis by regulating the liver insulin signaling pathway (Pang et al., 2009; Magdaleno et al., 2017). However, the exact mechanism remains unclear. Earlier, we found that miR-378b is upregulated in the liver tissues of ALD rats compared to ALD rats treated with methyl ferulic acid through high-throughput sequencing. Therefore, this study focused on the role and regulation of miR-378b in alcohol-induced liver insulin resistance. In this study, we show that the level of miR-378b is upregulated in the hepatic tissue ethyl alcohol (EtOH)-fed mice and EtOH-induced L-02 cells. This study shows that miR-378b directly targets the three prime untranslated region (3'UTR) of the IR and p110α genes to downregulate their protein expression, thereby disrupting insulin signaling. Hence, miR-378b upregulation in ALD is implicated in the pathogenesis of hepatic insulin resistance.

## Materials and Methods

### Animals and Protocols

Weight 20–25g male C57BL/6 mice, purchased by Hunan Sja Laboratory Animal Co., Ltd. (Hunan, China). The animal experiments have been approved by the Institutional Ethical Committee of Guilin Medical University. All experimental procedures were performed following the Health Guideline of Animal Use and Care Committee of Guilin Medical University. The mice were randomly assigned to either the control diet (CD)-fed group or EtOH-fed group. An National Institute on Alcohol Abuse and Alcoholism (NIAAA) model was constructed as earlier described with minor modifications (Bertola et al., 2013). Briefly, following a one-week period of acclimation using a control liquid diet, the mice of the EtOH-fed group were fed ethanol (5% v/v) liquid diets (LD) (Trophic Animal Feed High-Tech Co., Ltd., Nantong, China), whereas the mice in the CD-fed group received LD. All mice were pair-fed for four weeks. Mice were euthanized and we collected blood and liver tissues for further analysis.

### Adeno-Associated Virus Administration

The full mouse miR-378b sequences were amplified by PCR from mouse genomic DNA and cloned into pAAV-MCS digested by *BamH*I and *Hind*III. The adeno-associated virus (AAV, serotype 9, a gift from GeneChem, Shanghai, China), which has higher infection efficiency and induces long-lasting expression, was used in packaging recombinant AAV (Mingozzi and High, 2011). The packaged adeno-associated virus was named AAV-miR-378b. The empty (untransformed) adeno-associated virus vector named AAV-NC and served as control. Then, AAV-miR-378b, AAV-NC were given at a dose of 1 × 10^11^ vg per animal *via* tail vein injection using a 1-ml sterile syringe prior to ALD model construction.

### Tolerance Tests

Glucose-tolerance test (GTT) and Insulin-tolerance test (ITT) were carried out as previously described with minor modification (Qu et al., 2019). Glucose-tolerance test (GTT) was conducted on mice that were fasted overnight (12 h). After measuring fasted blood glucose (FBG) levels, each mouse received an intraperitoneal (i.p.) glucose injection at a dose of 2 g/kg body weight. Blood glucose levels in the tail vein were assessed after 15, 30, 60, and 120 min, respectively. Insulin-tolerance test (ITT) was performed using mice that were fasted for 6 h. After measuring FBG levels, every mouse was treated with insulin at a dose of 0.75 U/kg body weight as shown in the figures. Blood glucose levels were taken at indicated time points.

### Histopathological Analysis

Histopathological analysis was carried out as previously described with minor modification (Li et al., 2017). Liver tissues were fixed for 24 h in 10% phosphate-buffered formalin and buried in paraffin blocks, followed by staining for routine histological analysis. H&E staining was performed using standard protocols. Pathological changes were evaluated and photomicrographs were collected using an optical microscope (OLYMPUS BX41, OLYMPUS, Tokyo, Japan).

### Serum and Liver Biochemical Analysis

Hepatic lipids were extracted using a previously reported method (Cheng et al., 2018). Total triglycerides (TGs) in sera and livers were assessed using an Erba XL-600 automatic biochemical analyzer with TG determination kits (U82811020, Guilin Unitech Medical Electronics Co., Ltd., Guilin, China). The levels of alanine aminotransferase (ALT) and aspartate aminotransferase (AST) were measured using commercially available diagnostic kits (Guilin Elite Medical Electronics Co., Ltd., Guilin, China) by the Erba XL-600 automatic biochemistry analyzer. The levels of glucose and insulin were measured using biochemical (U83730050, Guilin Unitech Medical Electronics Co., Ltd., Guilin, China) and ultra-sensitive mouse insulin immunoassay kit (Li Ka Shing Faculty of Medicine, The University of Hong Kong) according to the supplier's protocol, respectively. Homeostasis model assessment of insulin resistance (HOMA-IR) index was calculated according to the formula: [fasting glucose level (mM)] × [fasting serum insulin (mU/L)]/22.5 (Du et al., 2018).

### Cell Culture

Human hepatocyte L-02 cells (no GDC079) were obtained from the China Center for Type Culture Collection of Wuhan University (Wuhan, China). The L-02 cells were cultured in Dulbecco's modified Eagle medium (DMEM) (817147, Thermo Fisher, MA, USA) medium containing 10% fetal bovine serum (FBS) (E600001-0100, Sangon Biotech, Inc., Shanghai, China) as well as 5% penicillin-streptomycin mixture (1212018, Sangon Biotech Inc., Shanghai, China). The cells were grown at 37°C in 5% CO_2_ air. In addition, our previous study found that 200 mM ethanol successfully induced insulin resistance in L-02 cells (Cheng et al., 2019), so we performed a subsequent experiment with an ethanol concentration of 200 mM.

### Measurement of Hepatocyte Glycogen Content

The glycogen content of L-02 cells and liver were assessed using glycogen kits (A043-1-1, Nanjing Jiancheng Bioengineering Institute, Nanjing, China) using the anthrone reagent method. Glycogen levels were normalized to the observed total protein contents. Total protein concentration was measured using the bicinchoninic acid assay (BCA) kit (P0010S, Beyotime Biotechnology, Beijing, China) and conducted following the supplier's protocols.

### Plasmid Construction and Transfection

The synthetic miR-378b mimic, inhibitor, and negative control RNA were obtained from GeneChem (Shanghai, China). Transfection was conducted using Gene Pulser Xcell™ Electroporation system (Bio-Rad Laboratories, Inc., Hercules, CA, USA) following the supplier's protocols. After 12 h transfection, the medium was changed to DMEM containing 200 mM ethanol. The treated cells were harvested after 48 h for the further analysis.

### RNA Isolation and Quantitative Real-Time PCR

Total RNA of liver tissues or L-02 cells were extracted using TRIzol reagent (DP419, Tiangen Biotech Co., Ltd., Beijing, China), following the supplier's protocol. Before reverse transcription using FastQuant RT Kit (KR106, Tiangen Biotech Co., Ltd., Beijing, China), total RNA was quantified in a NanoDrop One Ultra Micro Nucleic Acid Protein Analyzer (Gene Company Ltd., Kunming, China). Target genes were amplified using the MJ PTC-200 PCR system (Bio-Rad, Hercules, CA, USA) and the RT PCR kit (2× Taq PCR Master Mix, Aidlab Biotech Co., Ltd., Beijing, China). The PCR products were identified using 1.5% agarose gel electrophoresis. The mRNA level of β-actin was used as an internal control. For miRNA detection, total RNA was reverse-transcribed using All-in-One™ miRNA First-Strand cDNA Synthesis kit (QP103, GeneCopoeia™, Guangzhou, China) and subsequently measured by quantitative real-time (qRT)-PCR using miR-378b specific primer (GeneCopoeia™, Guangzhou, China). The U6 was used as the reference gene on the expression levels of miR-378b. Primer sequences were listed as following: p110α (forward 5'-CCACGACCATCATCAGGTGAA-3'; reverse 5'-CCTCACGGAGGC ATTCTAAAGT -3'), IR (forward 5'- AAAACGAGGCCCGAAGATTTC-3'; reverse 5'-GAGCCCATAGACCCGGAAG-3').

### Western Blotting and Antibodies

L-02 cells or liver tissues were lysed with RIPA lysis buffer (P0013B, Beyotime Institute of Biotechnology, Shanghai, China) supplemented with protease inhibitor (100×) and phosphatase inhibitor (C900369-0260, Sangon Biotech Inc., Shanghai, China). Protein concentrations were assessed using a modified form BCA protein concentration assay kit (Beyotime Bio Co., Nanjing, China). Proteins were separated by sodium dodecyl sulfate-polyacrylamide gel electrophoresis (SDS-PAGE) and then transferred onto the nitrocellulose membrane (Pall Co., NY, USA). Then, the membranes were incubated with the appropriate antibodies. The antigen-antibody complex on the membrane was detected with enhanced chemiluminescence (ECL) reagent (E002-100, 7sea Biotech Co., Shanghai, China). ImageJ was used for blotting and quantitative analysis. The intensity values were normalized to that of β-actin. The p110α antibody (#4249) was obtained from Cell Signaling Technology (Cell Signaling, Danvers, MA, USA). The phosphorylated AKT1 (D155022) and AKT1 (D151621) antibodies were purchased from Sangon Biotech Co., Ltd. (Shanghai, China). Primary antibodies against for p-IR Anti-Insulin Receptor (phospho Y1185) (ab62321), IR (ab131238), p85α (ab191606), AKT1 (ab227385), p-AKT1 (ab81283) were purchased from Abcam Technology (Cambridge, UK). The β-actin (TA-09), mouse anti-goat (EM3511-01) and goat anti-rabbit secondary antibodies (EM35110-01) were supplied by ZSGB Biotech Co., Ltd. (Beijing, China).

### Co-Immunoprecipitation Assay

To study the relationship between p85α and p110α, an immunoprecipitation assay was performed in accordance with the manufacturer's approach. After homogenized as described above, liver tissues or cell lysates were incubated overnight with specific antibodies at 4°C. Then, the mixture with the pre-formed antibody-antigen complex was added to the protein A-coupled beads (C600688-A, Sangon Biotech Inc., Shanghai, China). After incubating for 2 h at 4°C, discarded the supernatant, and the beads were washed seven times using cold phosphate-buffered saline (PBS). After the final wash, 50 μl of the sample buffer were added and boiled for western blotting.

### Luciferase Reporter Assays

To validate that IR/p110α gene is indeed the target of miR-378b, we purchased a plasmid harboring luciferase with the 3' UTR sequence of IR/p110α mRNA. Then, the mutant or wild-type of 3' UTR sequences of IR/p110α were inserted into the XbaI restriction sites of the pGL3 vector (GeneChem, Shanghai, China). 293T cells (Cell Bank of Chinese Academy of Sciences, Shanghai, China) infected with miR-378 mimics or ctrl RNA were seeded into 24-well plates. 0.6 μg pGL3 vector with above sequence was cotransfected with 0.05 μg pRL-TK vector into the cells by Lipofectamine 2000 (11668-019, Invitrogen, Carlsbad, CA, USA). At 48 h after transfection, 293T cells were harvested. The Dual-Luciferase Reporter Assay System (E2920, Promega, Madison, WI, USA) was used to measure luciferase activity.

### Statistical Analysis

All the experiments were conducted at least thrice independently, and the results were expressed as the means ± SD. A p value <0.05 was deemed statistically significant. Statistical significance was evaluated using an analysis of variance (ANOVA) and the student's t-test. The software used for data analysis and drawing were GraphPad Prism 7.0 and Excel. No data, samples, or animals were excluded or omitted from reporting.

## Results

### Expression Level of mir-378b Increased in Ethyl Alcohol Fed Mice

**Figure 1:**
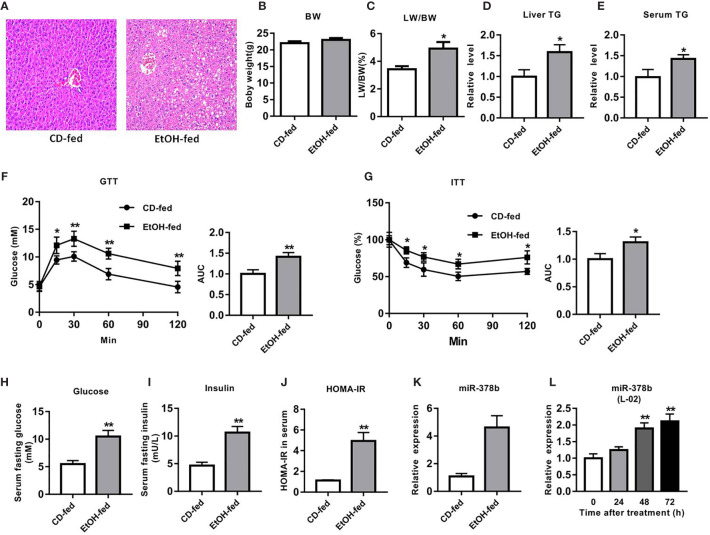
miR-378b is upregulated in EtOH-fed mice and EtOH-induced L-02 cells. **(A)** Representative images of H&E staining of liver sections from control diet (CD)-fed mice (left) or EtOH-fed mice (right). **(B)** Body weights of the mice before the experiment and before being killed. **(C)** The liver index. **(D)** Liver triglyceride (TG) levels. **(E)** Serum TG levels. **(F)** Glucose-tolerance test (GTT) and area under the curve (AUC) data. **(G)** Insulin-tolerance test (ITT) and area under the curve (AUC) data. **(H)** Fasting serum glucose levels. **(I)** Fasting serum insulin concentrations. **(J)** Homeostasis model assessment of insulin resistance (HOMA-IR) index. **(K)** The expression level of miR-378b in liver tissues. **(L)** The expression level of miR-378b in L-02 cells. All data are expressed as the mean ± SD of at least three separate experiments (n=6). *p < 0.05 *vs.* control; **p < 0.01 *versus* control.

To evaluate the liver damage in EtOH-fed and CD-fed mice, HE staining was performed. Histopathological analysis revealed that the liver tissues of EtOH-fed mice had numerous fat vacuoles of variable size, the liver cell cord was irregularly structured, and the hepatic cells disorganized, whereas the liver tissues in CD-fed mice exhibited normal lobular structure with radiating hepatic cords, without signs of fatty tissue degeneration (**Figure 1A**). Furthermore, there was no remarkable difference in the weights between EtOH-fed and CD-fed mice (**Figure 1B**). However, the liver index of the EtOH-fed group increased by 43.4% compared with the CD-fed group (**Figure 1C**). Compared with the control group, liver TG and serum TG levels in the EtOH-fed mice significantly increased by 44.7 and 59.2%, respectively (**Figures 1D, E**).

The EtOH-fed mice showed higher FBG levels and impaired glucose processing throughout the body by GTT and ITT (**Figures 1F, G**). The HOMA-IR index in EtOH-fed mice also significantly increased by 339.7% compared with the controls. The results clearly showed that glucose tolerance and insulin tolerance were impaired, and hyperglycemia developed in EtOH-fed mice, which are the characteristics of insulin resistance (**Figures 1H–J**). Because this study was centered on miRNAs that are related to ethanol-induced insulin resistance, we employed EtOH-fed mice in identifying miRNAs that are modulated by alcohol. qRT-PCR analysis showed that miR-378b expression in the model mice was over three-fold greater than that in CD-fed mice (**Figure 1K**). These results suggest that liver miR-378b high expression level is related to liver insulin resistance.

### miR-378b Is Upregulated in EtOH-Induced L-02 Cells

To study the function of mir-378b in the control of insulin sensitivity, the levels of miR-378b in L-02 cells was examined under normal and ethanol-induced conditions. Subsequently, L-02 cells were treated with ethanol for different durations, then the levels of miR-378b were assessed by qRT-PCR. Interestingly, the expression levels of miR-378b significantly increased in time dependent manner after ethanol treatment, and the levels of miR-378b in L-02 cells induced by ethanol for 48 and 72 h significantly increased by 65.2 and 111.7%, respectively, when compared with the control group (**Figure 1L**). Together, these results indicate that miR-378b is upregulated in the EtOH-treated L-02 cells, thereby implicating miR-378b in insulin resistance.

### Upregulation of miR-378b Disrupts Insulin Sensitivity in L-02 Cells

**Figure 2:**
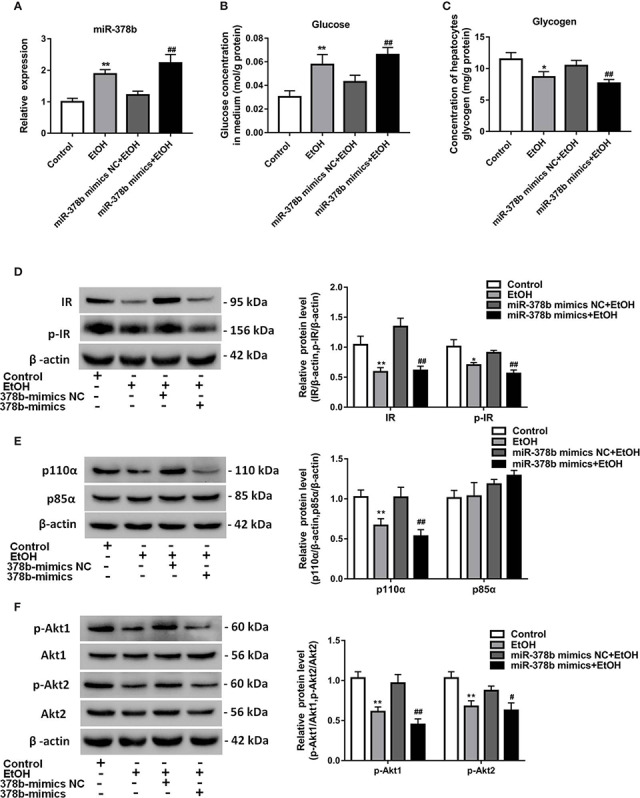
miR-378b overexpression suppresses insulin sensitivity in L-02 cells. **(A)** The expression level of miR-378b in L-02 cells. **(B)** The glucose levels in medium. **(C)** The glycogen content in L-02 cells. **(D)** Western blot analysis for protein expression of insulin receptor (IR) and p-IR. **(E)** Western blot analysis for protein expression of p85α and p110α. **(F)** Western blot analysis for protein expression of Akt1, Akt 2, p-Akt1, p-Akt2. All data are expressed as the mean ± SD of at least three separate experiments. ^∗^*p* < 0.05, ^∗∗^*p* < 0.01 *vs.* control. ^#^*p* < 0.05, ^##^*p* < 0.01 *vs.* miR-378b-mimics NC.

To research the impact of miR-378b on the insulin signaling pathway, L-02 cells were transfected with miR-378b mimic and incubated in the presence of ethanol. qRT-PCR analysis indicated that miR-378b level marked rise by 1.83-fold compared with the control group after miR-378b mimic transfection (**Figure 2A**). Moreover, the glucose levels of the medium in the miR-378b overexpression group and EtOH-induced group were significantly increased by 90.1 and 53.5%, respectively, when compared with the control group (**Figure 2B**). The miR-378b mimic also remarkable reduced glycogen levels by 26.7% in L-02 cells (**Figure 2C**). Furthermore, we analyzed the expression of the insulin signaling pathway in L-02 cells transfected with miR-378b mimic and found that the levels of IR and p-IR significantly decreased by 54.3 and 38.2%, respectively, after stimulation with EtOH relative to the control group (**Figure 2D**). In addition, the influence of p85α and p110α on the PI3K-AKT signaling pathway in hepatocytes were examined by immunoprecipitation. The results showed that the protein expression levels of p110α combined with p85α significantly decreased by 47.7% when transfected with miR-378b mimics and incubated in the presence of ethanol (**Figure 2E**). In addition, the protein expression levels of p-Akt1 and p-Akt2 markedly decreased by 53.4 and 28.2%, respectively, in the miR-378b overexpression group compared with the control groups (**Figure 2F**). In a word, these findings suggest that the over expressed miR-378b aggravates insulin resistance in L-02 cells.

### Suppression of miR-378b Alleviates EtOH-Induced Insulin Resistance *In Vitro*

**Figure 3:**
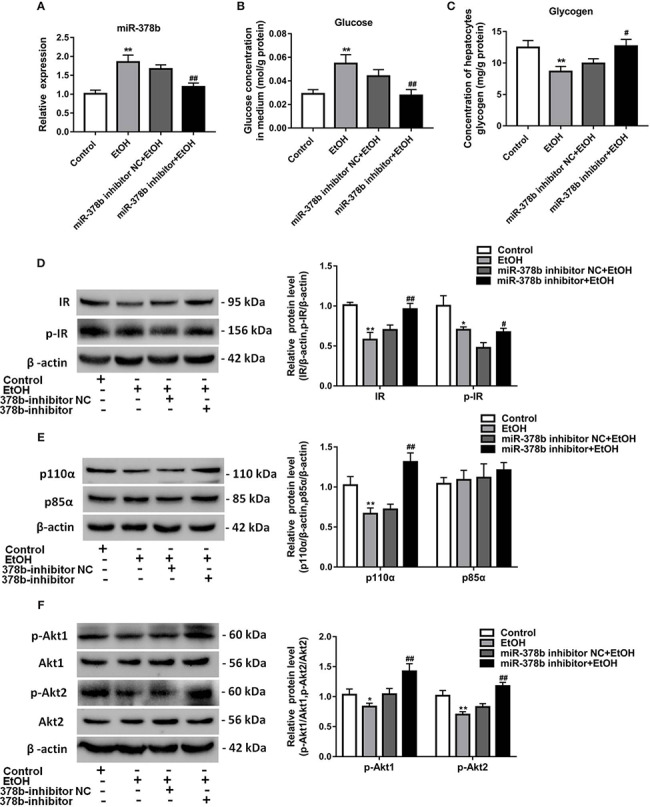
Suppression of miR-378b alleviates EtOH-induced insulin resistance *in vitro*. **(A)** The expression level of miR-378b in L-02 cells. **(B)** The glucose levels in medium. **(C)** The glycogen content in L-02 cells. **(D)** Western blot analysis for protein expression of insulin receptor (IR) and p-IR. **(E)** Western blot analysis for protein expression of p85α and p110α. **(F)** Western blot analysis for protein expression of Akt1, Akt 2, p-Akt1, p-Akt2. All data were expressed as the mean ± SD of at least three separate experiments. ^∗^*p* < 0.05, ^∗∗^*p* < 0.01 *vs.* control. ^#^*p* < 0.05, ^##^*p* < 0.01 *vs.* miR-378b-inhibitor NC.

The expression of miR-378b increased in EtOH-induced insulin resistant L-02 cells. Insulin sensitivity impaired by miR-378b overexpression was also observed in L-02 cells. Based on the results earlier described, we postulated that miR-378b inhibition also enhances insulin sensitivity in conditions of EtOH-induced insulin resistance. To verify our hypothesis, we transfected L-02 cells with miR-378b inhibitor and incubated these in the presence of ethanol. **Figure 3A** shows that the level of miR-378b significantly ascended by 83.2% in the ethanol group and transfection of the miR-378b inhibitor significantly decreased its level by 28.4% in L-02 cells. In addition, compared with the control group, the glucose levels in the medium significantly decreased by 37.1%, and glycogen levels significantly increased by 27.6% with transfection of miR-378b inhibitor in L-02 cells (**Figures 3B, C**). Furthermore, we analyzed the expression of the insulin signaling pathway in L-02 cells transfected with miR-378b inhibitor and found that the levels of IR and p-IR, significantly increased by 37.1 and 41.2%, respectively, in the L-02 cells transfected with miR-378b compared with the negative control groups (**Figure 2D**). Furthermore, immunoprecipitation showed that the protein levels of p110α and p85α significantly increased by 82.4% after transfection with the miR-378b inhibitor and incubated in the presence of ethanol (**Figure 3E**). In addition, the protein levels of p-Akt1 and p-Akt2 expressed in liver tissue significantly increased by 36.9 and 42.3%, respectively, in the miR-378b inhibitor group compared with the negative control groups (**Figure 2F**). As expected, these effects of ethanol were reversed after transfecting with the miR-378b inhibitor, indicating that inhibition of miR-378b increased insulin sensitivity in L-02 cells (**Figures 3D–F**).

### miR-378b Directly Targets IR and p110α

**Figure 4:**
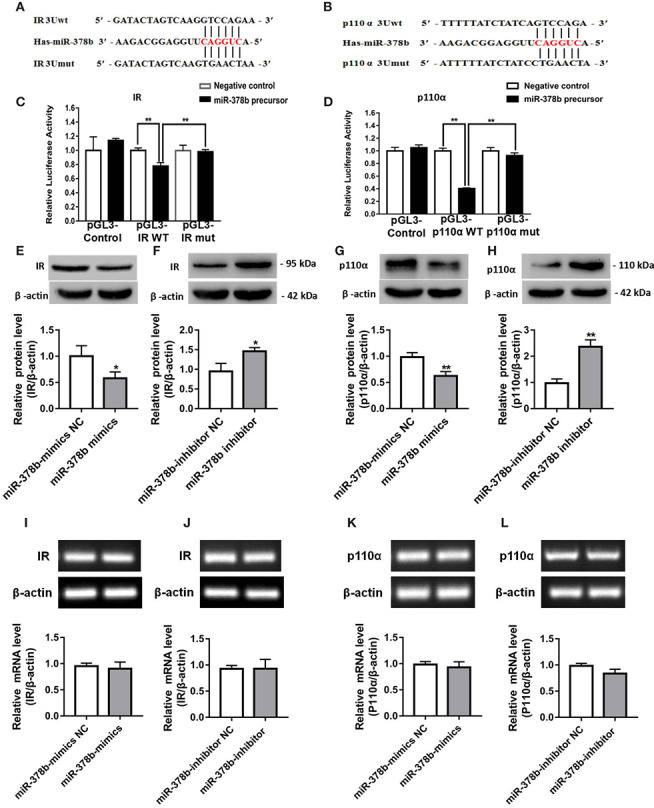
miR-378b directly targets IR and p110α. **(A, B)** Predicted duplex formation between miR-378b and human wild-type/mutant IR-3'-UTR (IR-3U-wt/mut) or human wild-type/mutant p110α-3'-UTR (p110α-3U-wt/mut). **(C, D)** Luciferase reporter assay for the interaction between wild-type 3'-UTR of insulin receptor (IR) or of p110α and miR-378b, as well as mutant 3'-UTR of IR or of p110α and miR-378b in the 293T cells. **(E–H)** Western blot analysis for protein expression of IR or p110α in L-02 cells. **(I–L)** Messenger RNA (mRNA) expression levels of IR or p110α in L-02 cells. All data were expressed as the mean ± SD of at least three separate experiments. ^∗^*p* < 0.05, ^∗∗^*p* < 0.01 *vs.* control.

To elucidate the molecular mechanism underlying the miR-378b-mediated insulin signaling pathway, using TargetScan bioinformatics tools, we hypothesized that IR and p110α are two potential targets of miR-378b, with their 3′ UTRs as miRNA-binding sites (**Figures 4A, B**). Bioinformatics analysis showed that IR and p110α are key components of insulin signaling. To further investigate whether these observed suppressive effects are direct actions, specific fragments of the wild-type and mutated IR and p110α 3′ UTRs were ligated to a luciferase reporter vector. The results showed that miR-378b mimics reduced luciferase activity by 26.2 and 129.8% of the reporter containing human IR and p110α-3′ UTRs, respectively, in 293T cells but had no effect on the reporters with mutated IR and p110α-3′ UTRs (**Figures 4C, D**), suggesting that IR and p110α are indeed miR-378b targets. To further determine whether p110α and IR are regulated by miR-378b, we transfected L-02 cells with either miR-378b mimics or inhibitor. IR and p110α proteins in the L-02 cells were respectively downregulated by 42 and 36.6% after transfection with the miR-378b mimics (**Figures 4E, G**). In contrast, L-02 cells transfected with miR-378b inhibitor resulted in the upregulation of IR and p110α by 54.2 and 142.8%, respectively (**Figures 4F, H**). No significant change in the mRNA level of IR and p110α (**Figures 4I–L**). On the basis of those study results, we suggested that the possible mechanism of miR-378b targeting IR and p110α is that miR-378b partially binds to the mRNA of IR and regulates the translation process of IR and p110α, but has no effect on the transcription process of IR and p110α.

### miR-378b Overexpression Aggravates Insulin Signaling *In Vivo*

**Figure 5:**
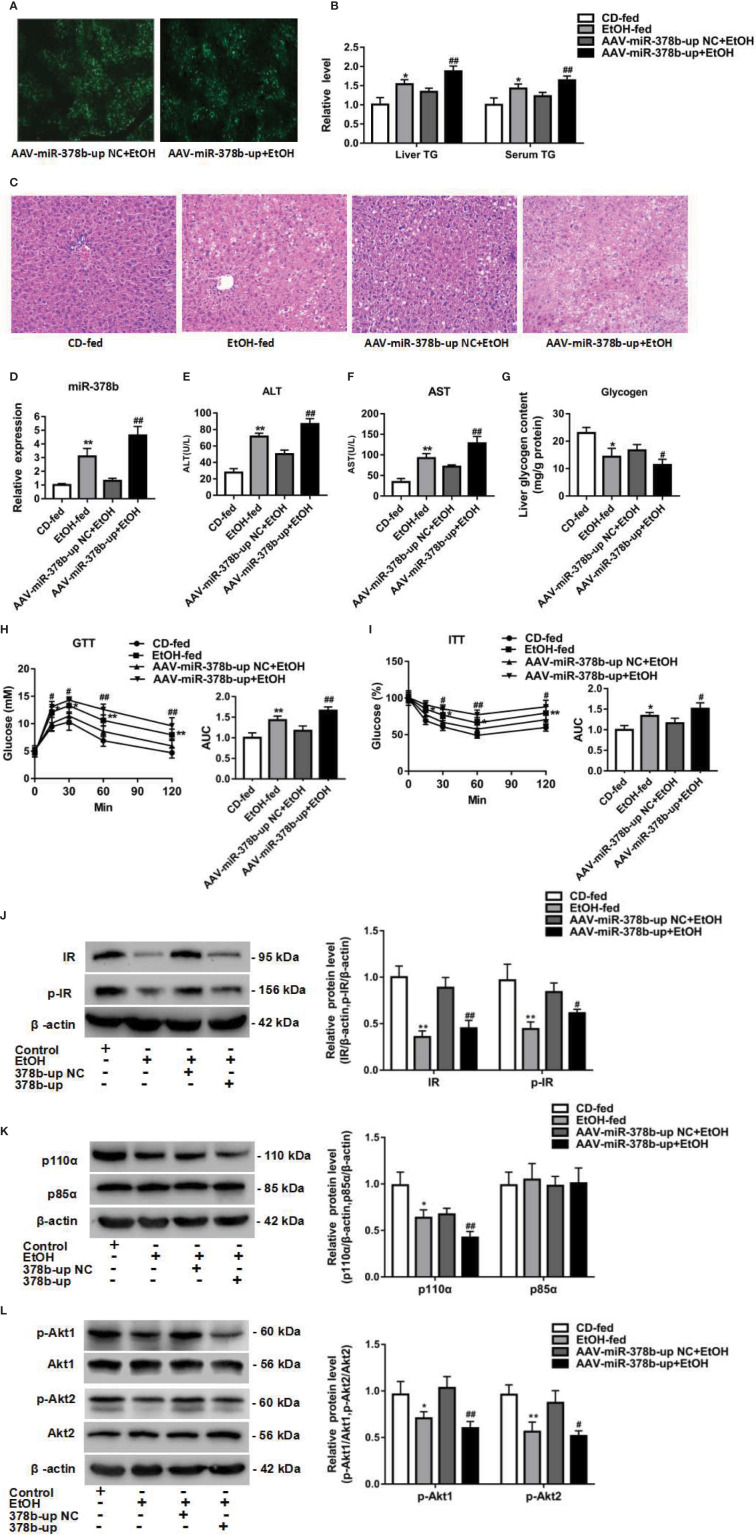
miR-378b overexpression aggravates insulin signaling *in vivo*. **(A)** The infection efficiency of AAV-miR-378b-up NC and AAV-miR-378b-up in liver tissue. **(B)** Liver and serum triglyceride (TG) levels. **(C)** Representative images of H&E staining of liver sections. **(D)** The expression of miR-378b in liver tissue. **(E)** Serum alanine aminotransferase (ALT) levels. **(F)** Serum aspartate aminotransferase (AST) levels. **(G)** Liver glycogen content. **(E)** Serum TG levels. **(H)** Glucose-tolerance test (GTT). **(I)** Insulin-tolerance test (ITT). **(J)** Western blot analysis for protein expression of insulin receptor (IR) and p-IR. **(K)** Western blot analysis for protein expression of p85α and p110α. **(L)** Western blot analysis for protein expression of Akt1, Akt 2, p-Akt1, p-Akt2. All data were expressed as the mean ± SD of at least three separate experiments (n=6). ^∗^*p* < 0.05, ^∗∗^*p* < 0.01 compared with the CD-fed group. ^#^*p* < 0.05, ^##^*p* < 0.01 compared with the AAV-miR-378b-up NC group.

To study the influence of miR-378b overexpression on EtOH-induced insulin resistance *in vivo*, we injected EtOH-fed mice with adeno-associated virus particles containing AAV-miR-378b-up or AAV-miR-378b-up-NC *via* tail vein. **Figure 5A** shows that the transfer effect of AAV-miR-378b-up or AAV-miR-378b-up-NC was almost the same in the tissue of EtOH-fed mice liver. MiR-378b expression in the liver of AAV-miR-378b-up-treated mice was 3.54-fold higher than that in the liver of the negative control mice (**Figure 5D**). Moreover, the TG levels in both serum and liver significantly increased by 34.1 and 39.8% in EtOH-fed mice after AAV-miR-378b-up injection (**Figure 5B**). The increase in lipid accumulation in EtOH-fed mice overexpressing miR-378b was further confirmed by H&E staining (**Figure 5C**). Moreover, the results showed that the ALT and AST levels were significantly increased by AAV-miR-378b-up-treated (**Figures 5E, F**). In addition, compared with the AAV-miR-378b-up-NC group, the glycogen levels significantly decreased by AAV-miR-378b-up-treated (**Figures 5G**). Furthermore, miR-378b overexpression resulted in impaired glucose tolerance and insulin tolerance (**Figures 5H, I**). These results show that miR-378b overexpression influences insulin signaling.

The above findings prompted us to investigate the effect of miR-378b on the insulin signaling pathway. Therefore, to assess whether the upregulation of miR-378b induces insulin resistance in EtOH-fed mice, we injected miR-378b into EtOH-fed mice and assessed the phosphorylation of various insulin signaling intermediates. MiR-378b overexpression significantly reduced IR phosphorylation and IR expression by 49.2 and 26.9%, respectively, in EtOH-fed mice (**Figure 5J**). In addition, phosphorylation of the downstream molecules of IR such as Akt1, and Akt2 were significantly reduced by 41.9%, and 40.8%, respectively (**Figures 5J, L**). Furthermore, the protein expression of p110α bound to p85α was also significantly reduced by 37.1% in EtOH-fed mice injected with AAV-miR-378b-up (**Figure 5K**). Based on these results, we proposed that the over expressed miR-378b aggravates insulin resistance *in vivo*.

### Loss of miR-378b Improves Insulin Resistance in EtOH-Fed Mice

**Figure 6:**
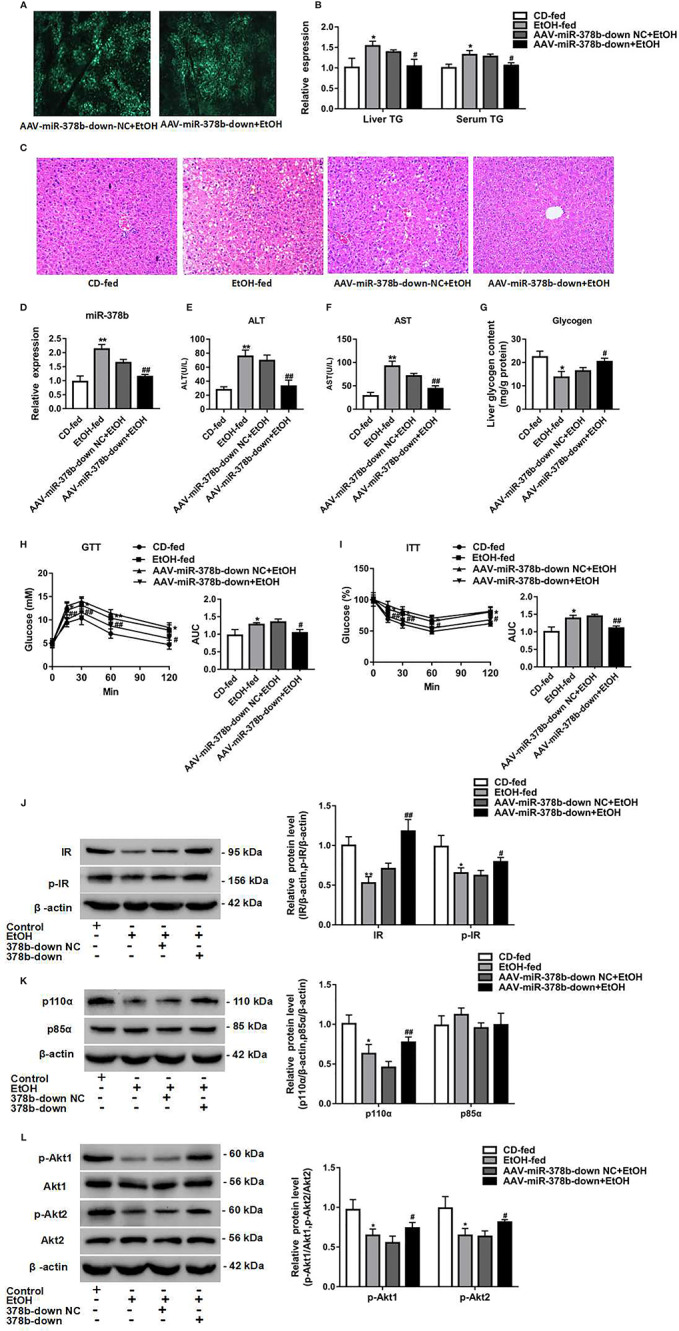
Loss of miR-378b improves insulin resistance in EtOH-fed mice. **(A)** The infection efficiency of AAV-miR-378b-down NC and AAV-miR-378b-down in liver tissue. **(B)** Liver and serum triglyceride (TG) levels. **(C)** Representative images of H&E staining of liver sections. **(D)** The expression of miR-378b in liver tissue. **(E)** Serum alanine aminotransferase (ALT) levels. **(F)** Serum aspartate aminotransferase (AST) levels. **(G)** Liver glycogen content. **(E)** Serum TG levels. **(H)** Glucose-tolerance test (GTT). **(I)** Insulin-tolerance test (ITT). **(J)** Western blot analysis for protein expression of insulin receptor (IR) and p-IR. **(K)** Western blot analysis for protein expression of p85α and p110α. **(L)** Western blot analysis for protein expression of Akt1, Akt 2, p-Akt1, p-Akt2. All data were expressed as the mean ± SD of at least three separate experiments (n=6). ^∗^*p* < 0.05, ^∗∗^*p* < 0.01 compared with the control diet (CD)-fed group. ^#^*p* < 0.05, ^##^*p* < 0.01 compared with the AAV-miR-378b-down NC group.

Suppression of miR-378b protected hepatocytes from EtOH-induced insulin resistance. Next, the results observed in L-02 cells were extended to mouse models of EtOH-fed insulin resistance. Adeno-associated virus expressing a miR-378b-inhibitor (AAV-miR-378b-down) or a negative control adeno-associated virus vector containing GFP (AAV-miR-378b-down-NC) were injected from the tail vein into the liver of mice. The adeno-associated virus showed fluorescence, and the transfection efficiency of AAV-miR-378b-down or AAV-miR-378b-down-NC was almost the same by fluorescence microscopy (**Figure 6A**). Transfection of AAV-miR-378b-down reduced the expression of miR-378b by 30.3% compared with the AAV-miR-378b-down-NC group (**Figure 6D**). Moreover, AAV-miR-378b-down infection resulted in a marked reduction in hepatic TG content by 17.2%, as well as serum TG levels by 24.7% in EtOH-fed mice (**Figure 6B**). The H&E staining revealed that the hepatic lipid accumulation levels were significantly elevated compared with the controls in both chronic alcohol diet-fed mice as well as AAV-miR-378b-down-NC infected mice (**Figure 6C**). Moreover, the results showed that the increased levels of ALT and AST induced by ethanol in serum were significantly attenuated by AAV-miR-378b-down- treated (**Figures 6E, F**). In addition, compared with the AAV-miR-378b-down-NC group, the glycogen levels were significantly increased by AAV-miR-378b-down-treated (**Figures 6G**). In contrast, administration of AAV-miR-378b-down significantly reduced the development of alcoholic insulin resistance. GTT showed that exogenous glucose was cleared faster in AAV-miR-378b-down-treated mice than in AAV-miR-378b-down-NC-treated mice. Meanwhile, ITT revealed that injection of miR-378b increased insulin sensitivity in EtOH-fed mice (**Figures 6H, I**).

To address the mechanisms underlying EtOH-induced insulin resistance regulation by miR-378b, we tested the action of miR-378b on the expression of insulin signaling intermediates by adeno-associated virus-mediated inhibition of miR-378b. As expected, western blot analysis showed that inhibition of miR-378b significantly increased IR levels by 67% and IR, Akt1, and Akt2 phosphorylation levels by 68.1, 41.6, and 28.4%, respectively, in EtOH-fed mice (**Figures 6J, L**). Moreover, we assessed the effects of p85α and p110α on liver PI3K-AKT signaling pathway using immunoprecipitation. p85α protein expression levels in mice did not change with injection of AAV-miR-378b-down. However, the protein expression of p110α bound to p85α was significantly increased by 69.3% in EtOH-fed mice injected with AAV-miR-378b-down (**Figure 6K**). Collectively, these findings indicate that inhibition of miR-378b in EtOH-fed mice improves insulin resistance.

## Discussion

Growing evidence suggests that miRNAs play central roles in various aspects of insulin resistance. Furthermore, the development of insulin resistance controls the progression from simple steatosis toward cirrhosis in ALD (Carr and Correnti, 2015). Thus, elucidating the mechanistic processes underlying insulin signaling may improve our understanding of the development of alcohol-induced insulin resistance. In the present study, we found that miR-378b expression boosted in the EtOH-fed mice and EtOH-trigger L-02 cells and at the same time, it was accompanied with insulin signal pathway dysfunction. Further investigation show that the over expressed miR-378b reduced PI3K/AKT activation, whereas inhibition of miR-378b improved insulin resistance *in vivo and in vitro*. Our findings demonstrate for the first time that miR-378b has a key role in alcohol-induced hepatic insulin resistance.

MiRNA can directly or indirectly regulate insulin sensitivity through epigenetic mechanisms (Yang et al., 2012). The possible targets of differentially expressed miRNAs have central roles in insulin secretion, lipid metabolism, glucose metabolism, and inflammation, which are the main process involved in the occurrence and development of alcoholic liver disease (Hartmann and Tacke, 2016; Satishchandran et al., 2018). A previous study has shown that mice with liver fibrosis have downregulated miR-378b levels in the liver (Hyun et al., 2016). However, our results suggested that miR-378b expression in the livers of NIAAA mice (EtOH-fed) were significantly increased. Some factors such as feeding periods, different diet compositions, and degree of liver damage may explain the observed contradiction. Previous reports have display that the expression patterns of circulatory and hepatic miRNA shows a reverse trend (Coulouarn et al., 2009; Choi et al., 2013). Furthermore, a study suggested that alcohol overconsumption and overnutrition could promote the development of insulin resistance (Carr and Correnti, 2015). Furthermore, the metabolic disorders of rodent species are complex and related to other factors, including elevated levels of glucose, lipid, as well as hormones. Therefore, to determine which factor controls the expression of miR-378b, we treated L-02 cells with ethanol. Our results showed that treatment with ethanol notably raised the miR-378b expression level and disrupted the phosphorylation levels of insulin signaling pathway. This study data suggest that ethanol controls miR-378b expression, and miR-378b may be involved in hepatic insulin resistance.

miR-378b, a member of the miR-378 family, is highly conserved across species, from chimp to humans. A previous study has shown that miR-378b is expressed in normal human dermal fibroblasts, and circulating miR-378b is a sensitive biomarker for photo-aging (Joo et al., 2017). It was also found to protect dermal fibroblasts from UVB-inhibited collagen I by targeting SIRT6 (Kim et al., 2017). However, the role of miR-378b in the pathogenesis of hepatic insulin resistance remains elusive. To determine the role of miR-378b in hepatic insulin resistance, we transfected miR-378b mimics or inhibitor or NC into L-02 cells. As expected, our research data indicated that the over expressed miR-378b lowered insulin sensitivity and caused the insulin signaling pathway dysfunction. On the contrary, further study indicated that miR-378b blocker markedly improved insulin sensitivity in L-02 cells. These findings suggest that the liver plays a central role in glucogenesis and lipogenesis (Baranowski et al., 2014). In this study, we observed that the overexpression of miR-378b in L-02 cells markedly decreased glycogen content, whereas the inhibition of miR-378b enhanced glycogen content. In brief, our research demonstrated that miR-378b has a negative effect on insulin signaling pathway *in vitro*.

In the current study, two targets of miR-378b, IR and p110α, have been identified and experimentally verified. IR is a ligand-activated receptor and member of the tyrosine kinase family of transmembrane signaling proteins, which are collectively known as major regulators of cell growth, differentiation, and metabolism (Lee and Pilch, 1994). Furthermore, IR also serves as an important center of Insulin signal transduction, with its activation tightly regulated. During insulin stimulation, IR is activated *via* autophosphorylation, which consequently phosphorylates numerous IRS proteins. PI3K, which is composed of a catalytic subunit p85 and regulatory subunit p110, functions in various pathological and physiological conditions, including metabolic regulation, immune responses, and cancer (Pentassuglia et al., 2016). p110α, a subunit of Pi3k that is known to be highly expressed in mammalian tissues. Previous studies on p110α have focused on breast (Bhat-Nakshatri et al., 2016) and lung cancer (Bonelli et al., 2015). Nevertheless, our understanding of biological effect of p110α in ethanol-induced insulin resistance is limited. A previous study suggested that inhibition of IR or p110α significantly decreases AKT phosphorylation (Liu et al., 2014; Srivastava et al., 2018). In this research, our data indicated that the levels of IR and p110α were significantly lowered in the liver of NIAAA mice. Furthermore, In L-02 cells, miR-378b is usually negatively correlated with IR or P110. In addition, using TargetScan, IR and p110α were identified as miR-378b target genes. Luciferase constructs harboring the 3'-UTRs of IR or p110α resulted in significantly lower activity in 293T cells that were overexpressing miR-378b. To verify the role of miR-378b in the downregulation of IR or p110α, we transfected miR-378b mimics, inhibitor, or NC into L-02 cells. MiR-378b overexpression resulted in a decrease in IR or p110α expression, whereas the downregulation of miR-378b improved IR or p110α expression. These findings further support the contribution of miR-378b in hepatic insulin resistance.

We also established an NIAAA mice model using Lieber-DeCarli liquid diet supplemented with 5% ethanol. We observed that miR-378b expression increased in the livers of EtOH-fed mice. Furthermore, our research also verified the impairment of the insulin signaling pathway in the liver of EtOH-fed mice relative to the NC group. We conducted a gain-of-function experiment using miR-378b. After tail vein injection of AAV-miR-378b or AAV-NC, at least a two-fold increase in hepatic miR-378b expression was observed in the livers of EtOH-fed mice relative to the NC group. Remarkably, the over expressed hepatic miR-378b pronouncedly increased TG levels and disrupted insulin signaling, whereas inhibition of miR-378b significantly reduced TG levels and enhanced insulin signaling by downregulating IR/p110α. Our findings indicate that the upregulation of miR-378b contributes to hepatic insulin resistance in EtOH-fed mice. In sum, Our research show that miR-378b has a negative effect on activation of the insulin signaling pathway *in vitro*.

In conclusion, our researches show that excessive drinking can induce high expression of miR-378b. miR-378b has incomplete binding to IR and p110α mRNA 3-UTRs, so it does not affect the stability of IR and p110α mRNA and does not affect the transcription process. Instead, it can affect the protein translation process of IR and p110α mRNA, so that IR and p110α proteins are down-regulated in the liver, and AKT phosphorylation is reduced, affecting the insulin signaling cascade in the liver. Due to the inhibition of the PI3K/AKT pathway, glycogen synthesis in the liver is reduced and blood glucose is increased, which promote the *de novo* synthesis of liver fatty acids and increase adrenoleukodystrophy (VLDL) secretion, leading to fatty liver and hyperlipidemia. High expression of miR-378b in the liver is closely related to ALD formation, and miR-378b is a promising new target for ALD drug therapy.

## Data Availability Statement

All data generated or assessed during this study are presented in the published article.

## Ethics Statement

The animal study was reviewed and approved by the institutional ethical committee of Guilin Medical University.

## Author Contributions

Y-YL and Y-JZ conducted experiments, analyzed data, and wrote manuscript. Y-ZW, Y-YF, C-FY, and ZM conducted experiments and analyzed data. LL, Y-WL, and QC analyzed data and revised manuscript. LL and Y-WL served as the guarantors of this work and as such, received full access to all the results in the study and take full responsibility for data integrity and analytical accuracy.

## Funding

This research was supported by the National Natural Science Foundation of China (NO. 81760669; 81860660), the Guangxi Natural Science Foundation Project of Guangxi Province, China (Nos. 2017GXNSFAA198259; 2018GXNSFDA281012).

## Conflict of Interest

The authors declare that the research was conducted in the absence of any commercial or financial relationships that could be construed as a potential conflict of interest.
