# Supplementary figures and images for: miR-378b Regulates Insulin Sensitivity by Targeting Insulin Receptor and p110α in Alcohol-Induced Hepatic Steatosis
Source: Front Pharmacol. 2020 May 20;11:717. doi: 10.3389/fphar.2020.00717 (PMC7251170; doi:10.3389/fphar.2020.00717)

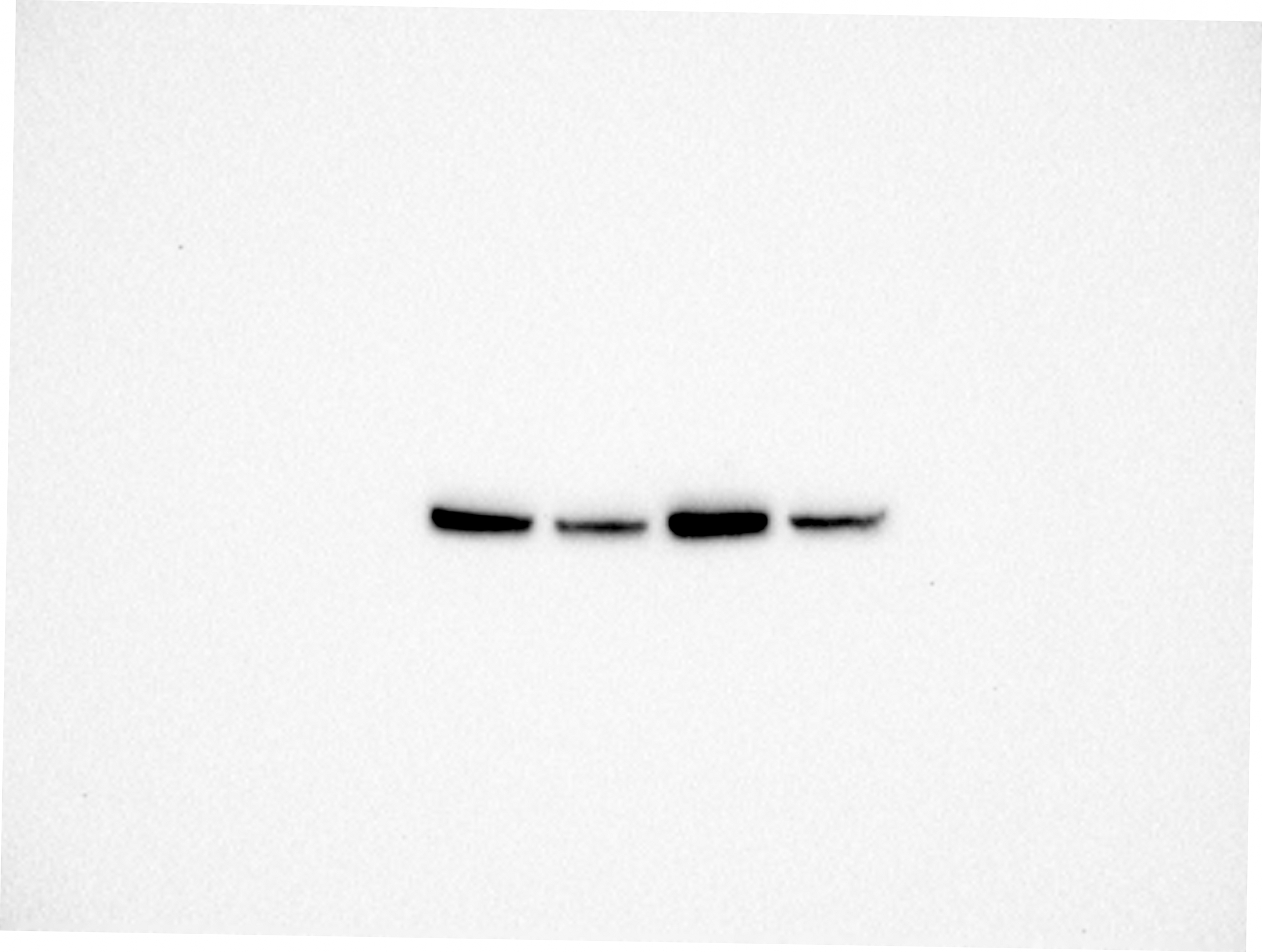

Supplement: Supplementary file 1 [file DataSheet_1.zip › 1A.tif]

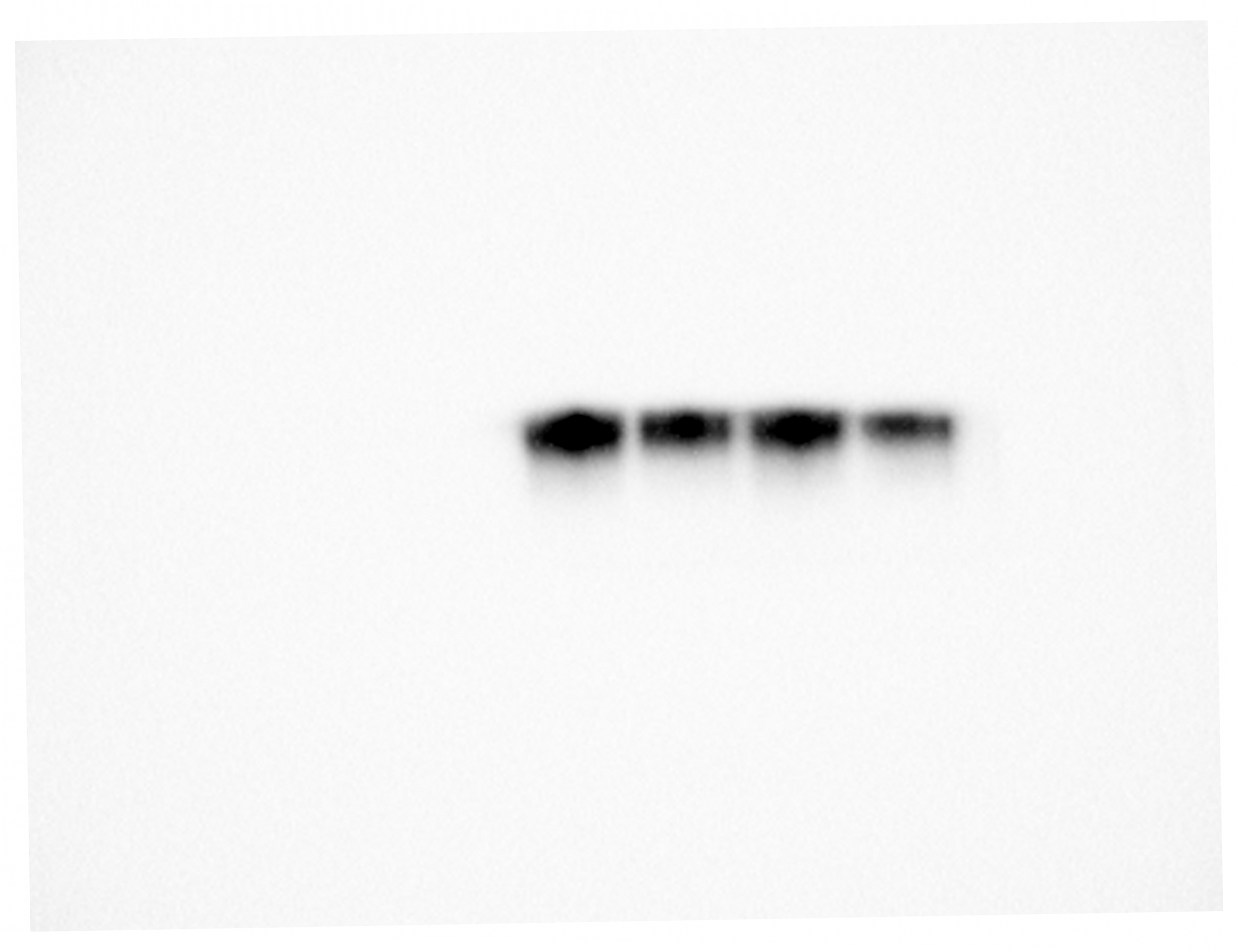

Supplement: Supplementary file 1 [file DataSheet_1.zip › 1B.tif]

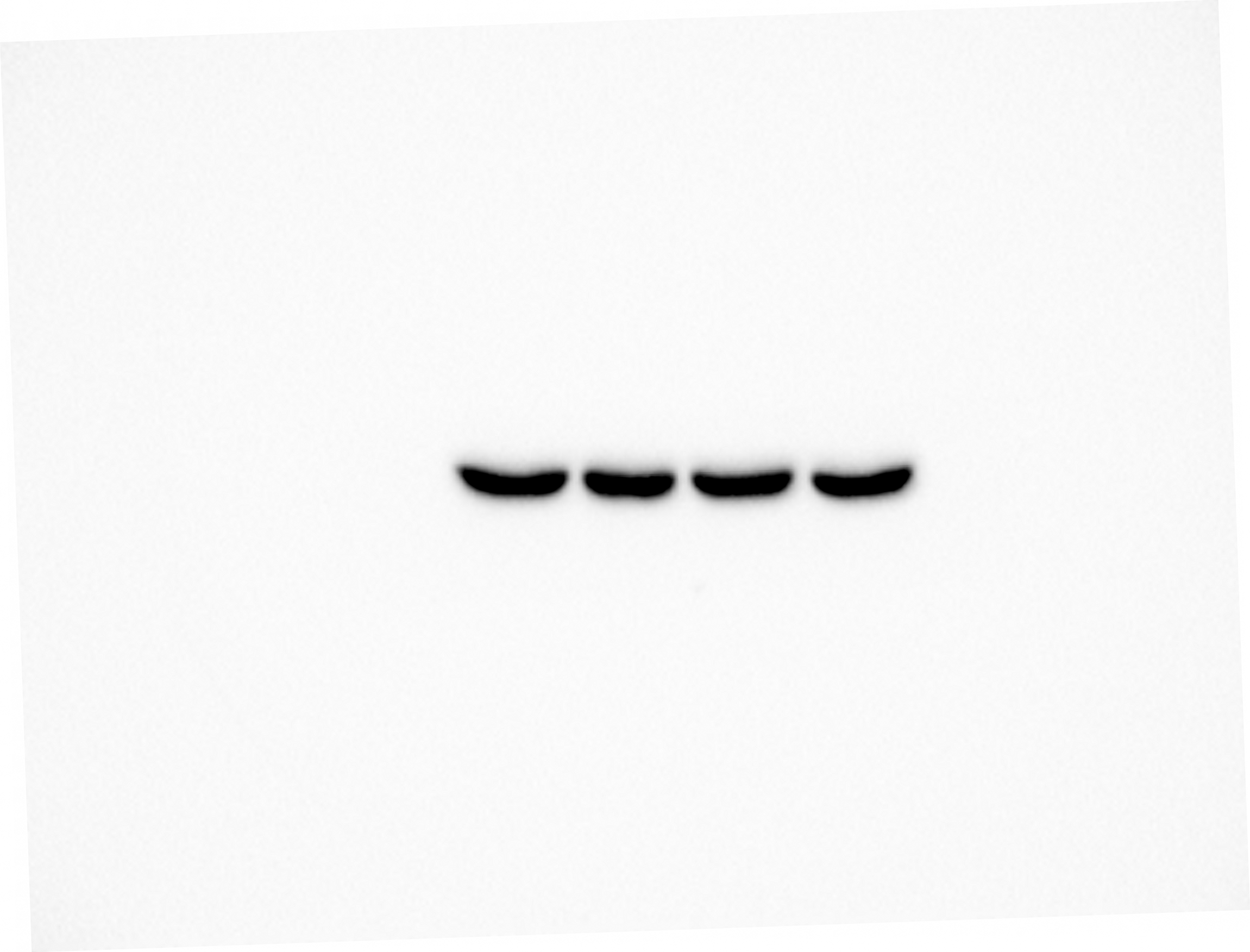

Supplement: Supplementary file 1 [file DataSheet_1.zip › 1C.tif]

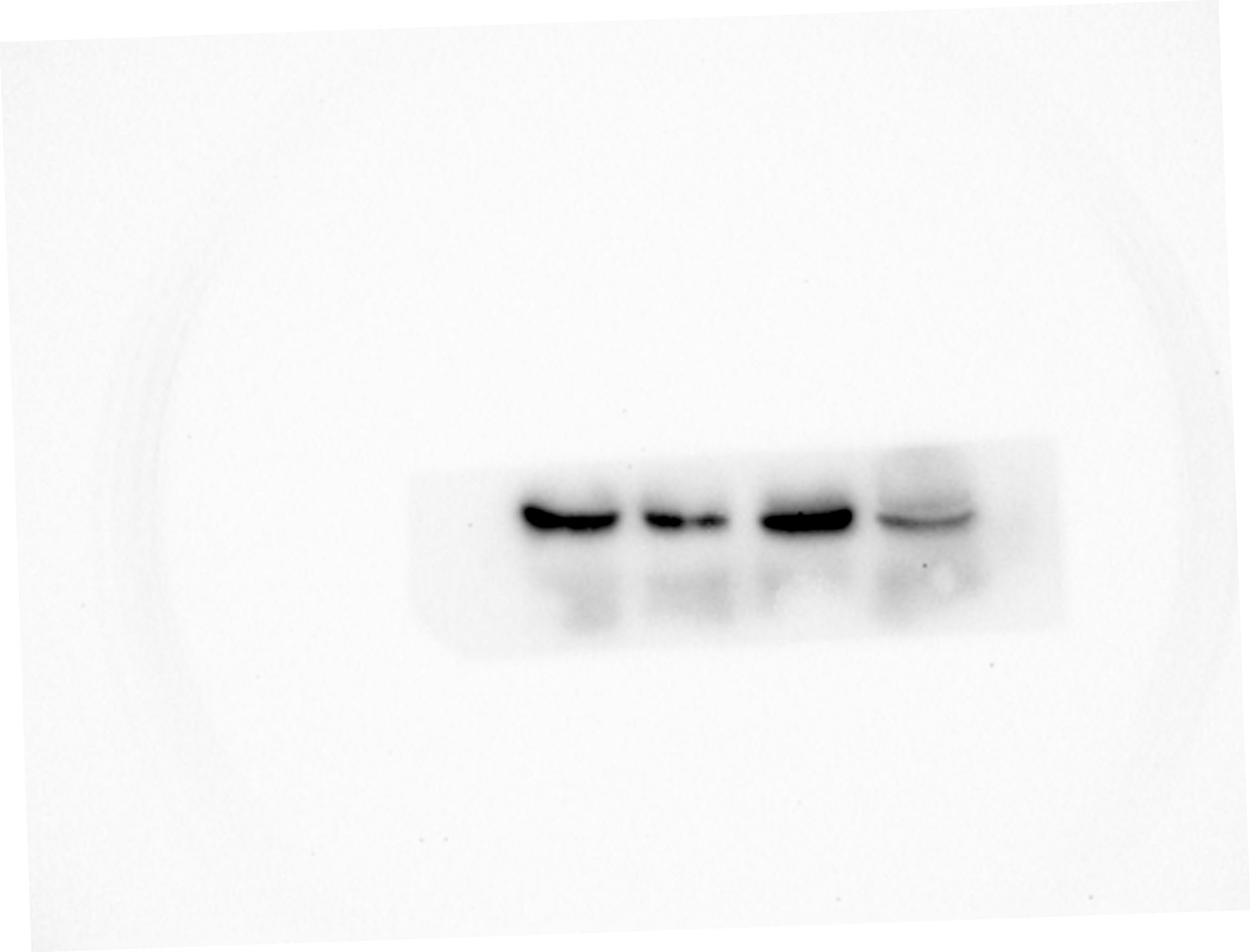

Supplement: Supplementary file 1 [file DataSheet_1.zip › 1D.tif]

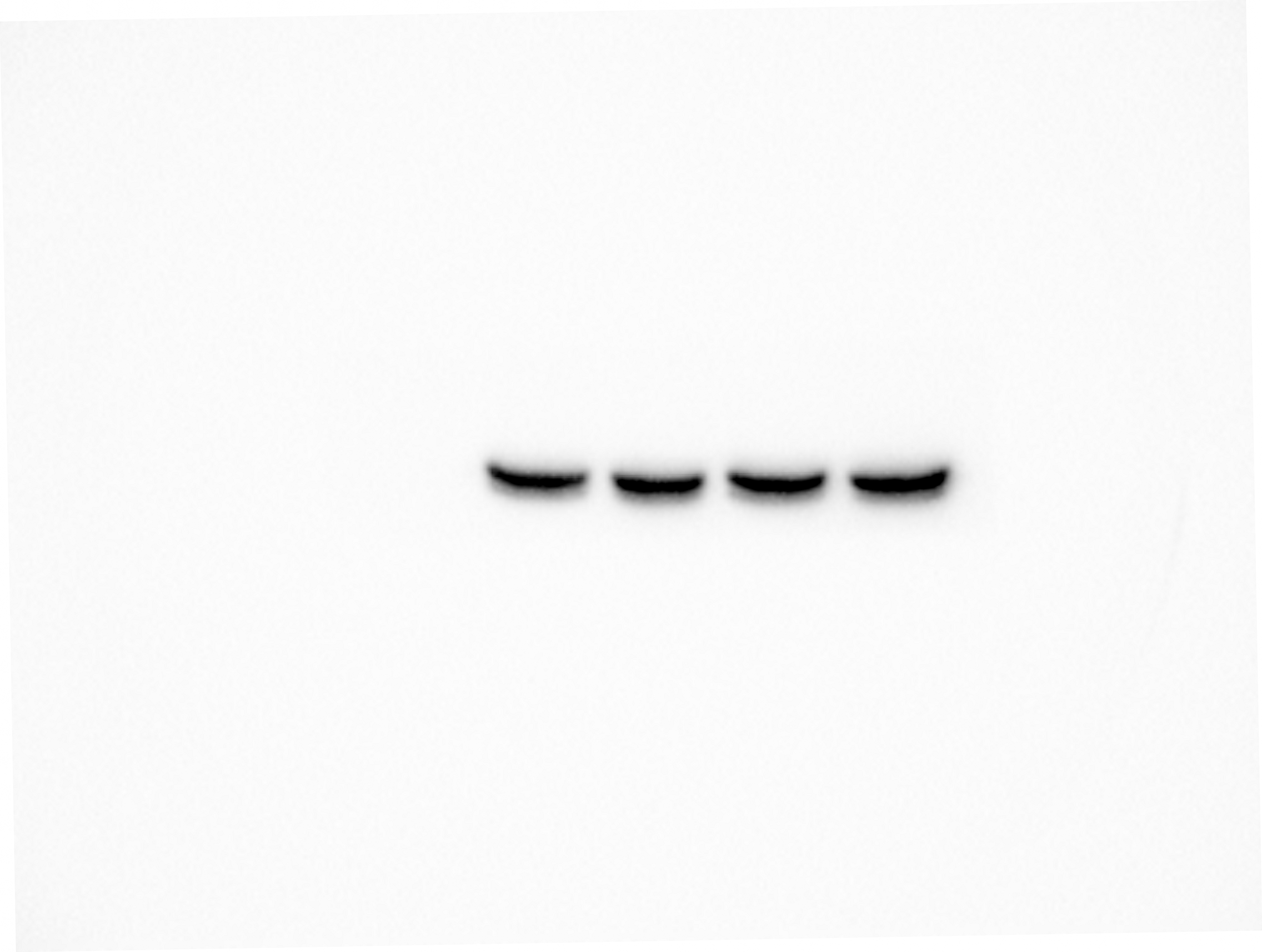

Supplement: Supplementary file 1 [file DataSheet_1.zip › 1E.tif]

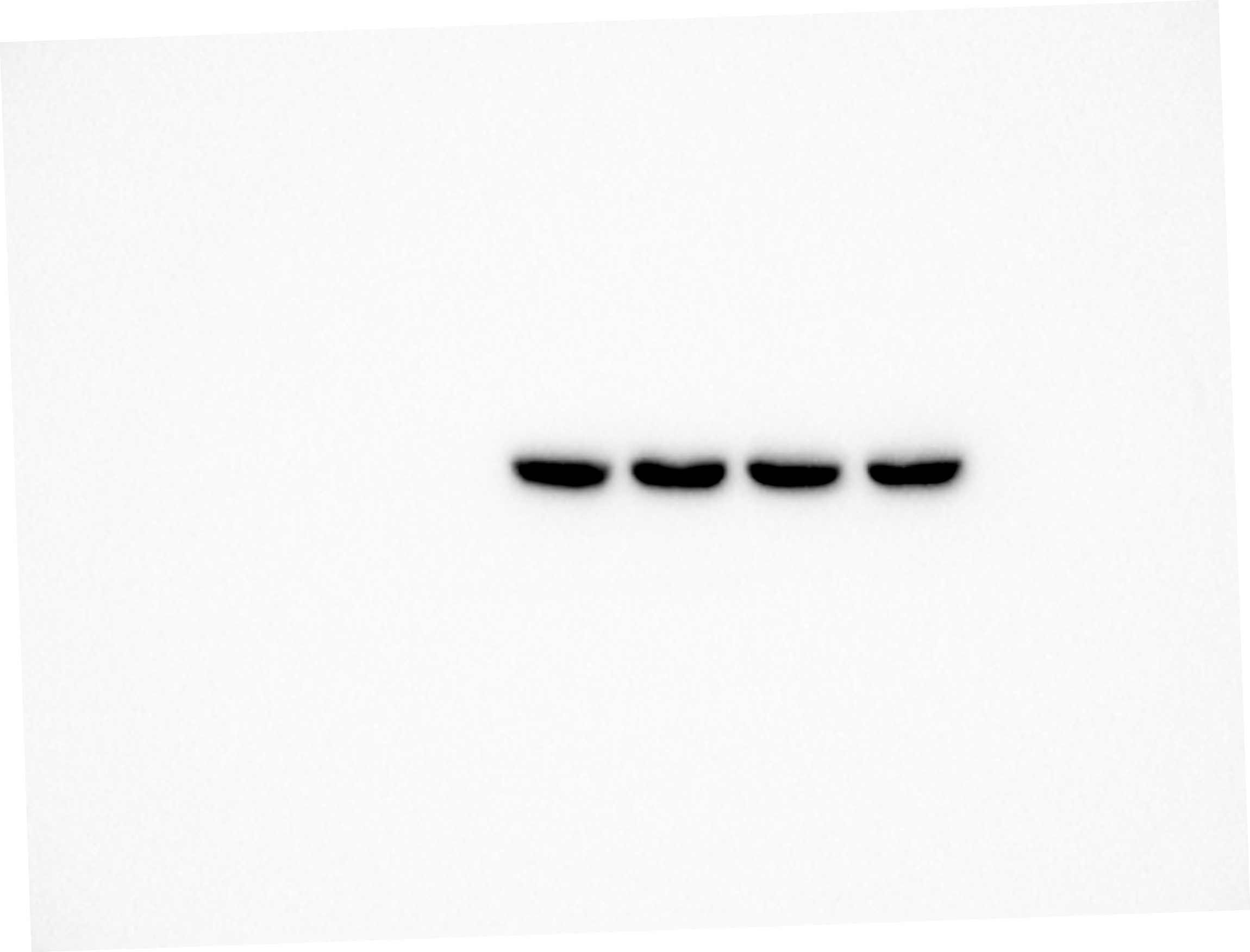

Supplement: Supplementary file 1 [file DataSheet_1.zip › 1F.tif]

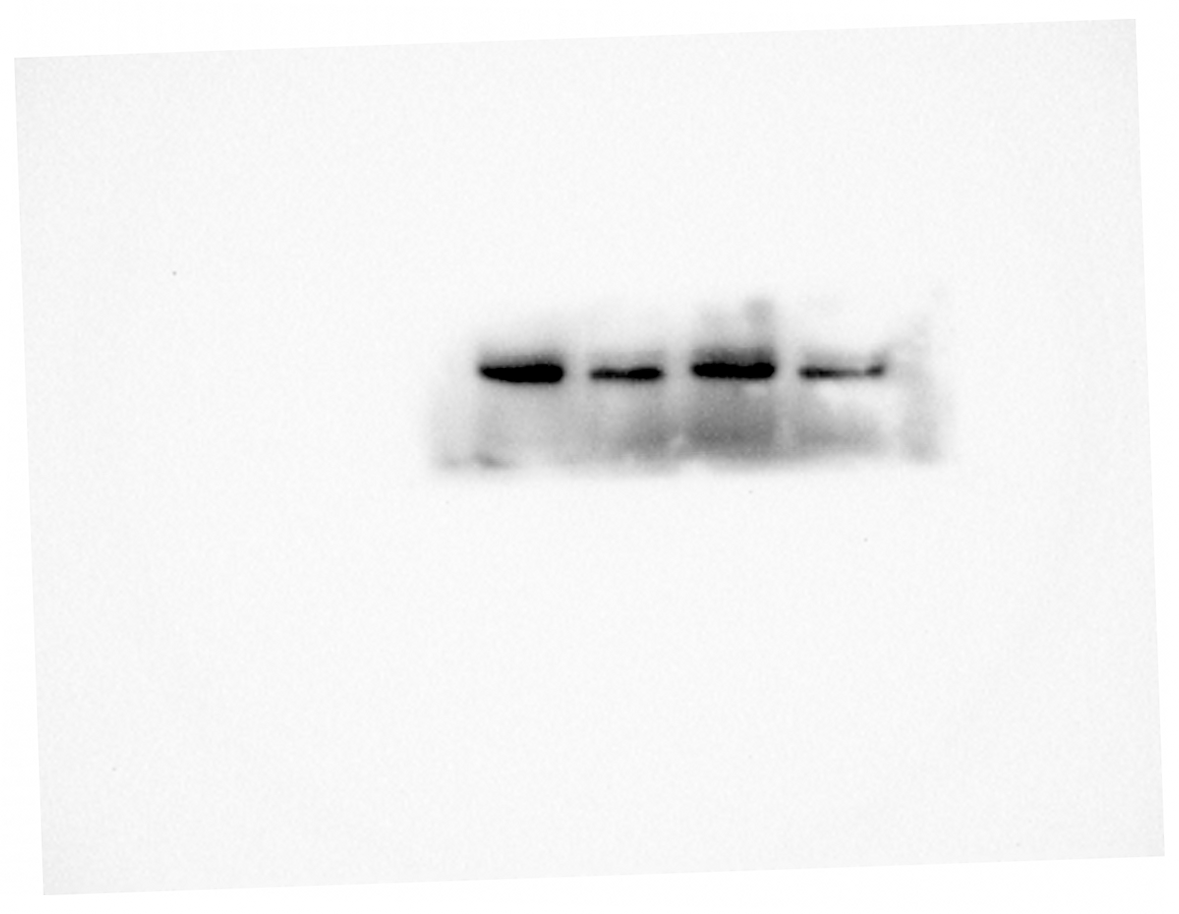

Supplement: Supplementary file 1 [file DataSheet_1.zip › 1G.tif]

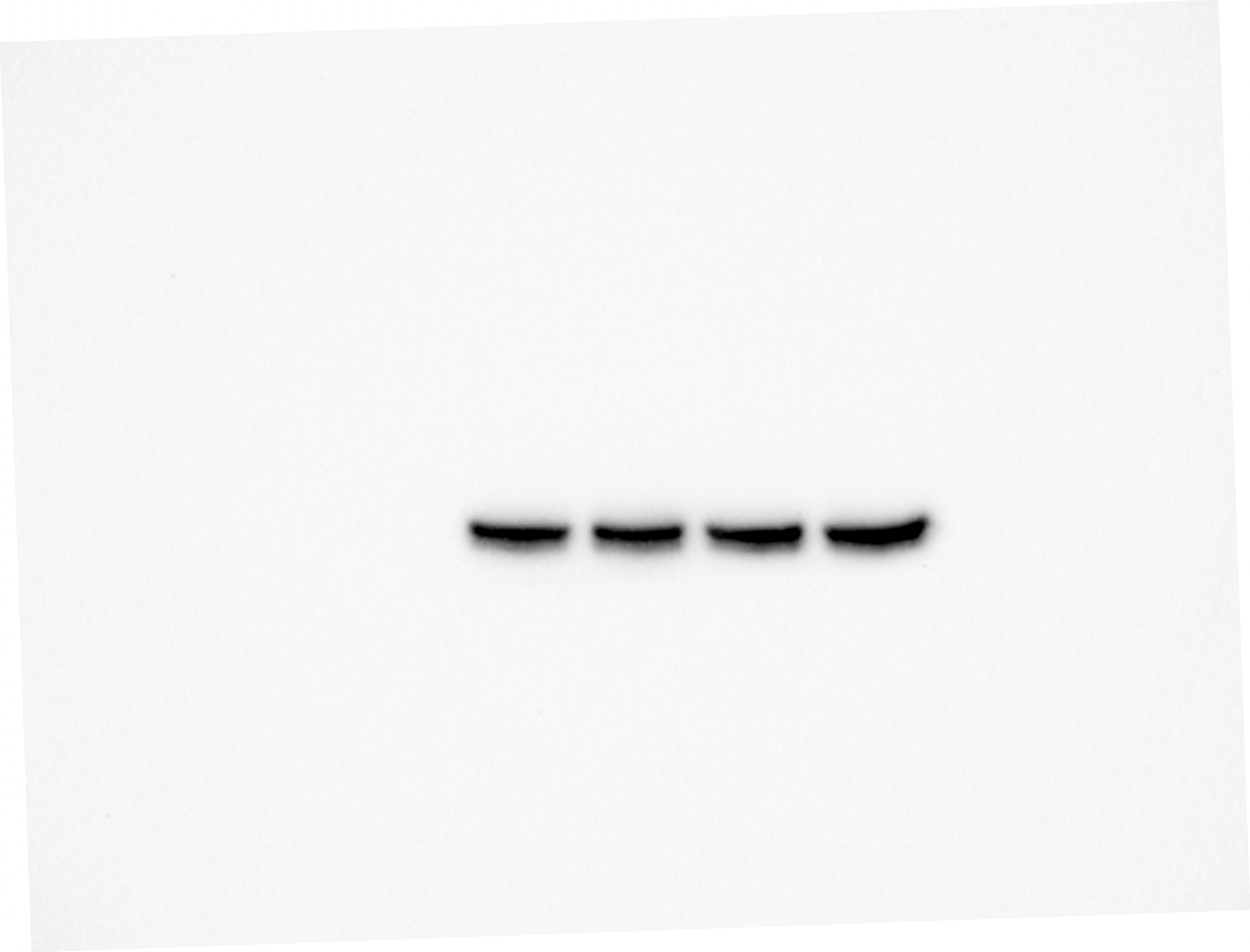

Supplement: Supplementary file 1 [file DataSheet_1.zip › 1H.tif]

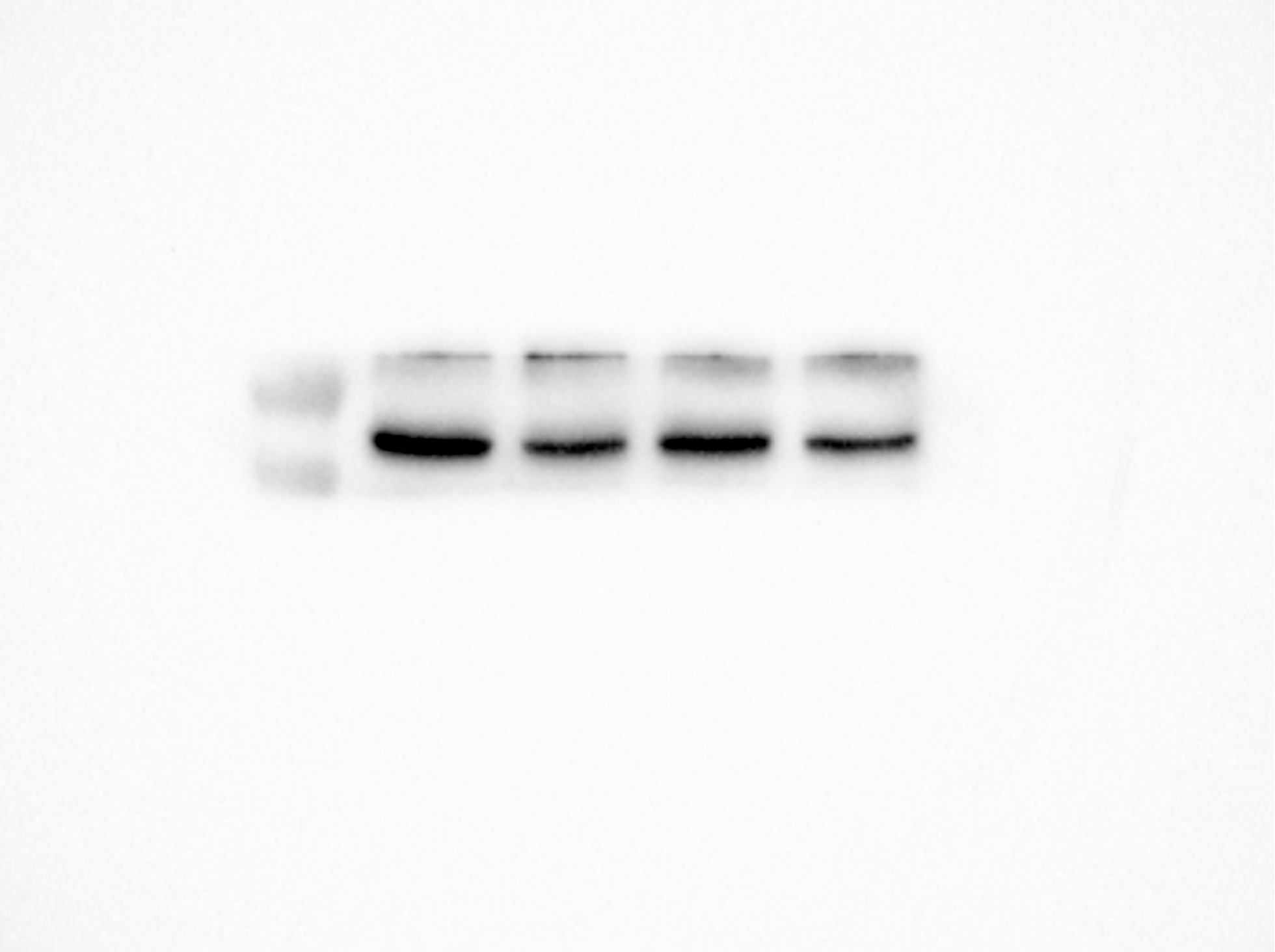

Supplement: Supplementary file 1 [file DataSheet_1.zip › 1I.tif]

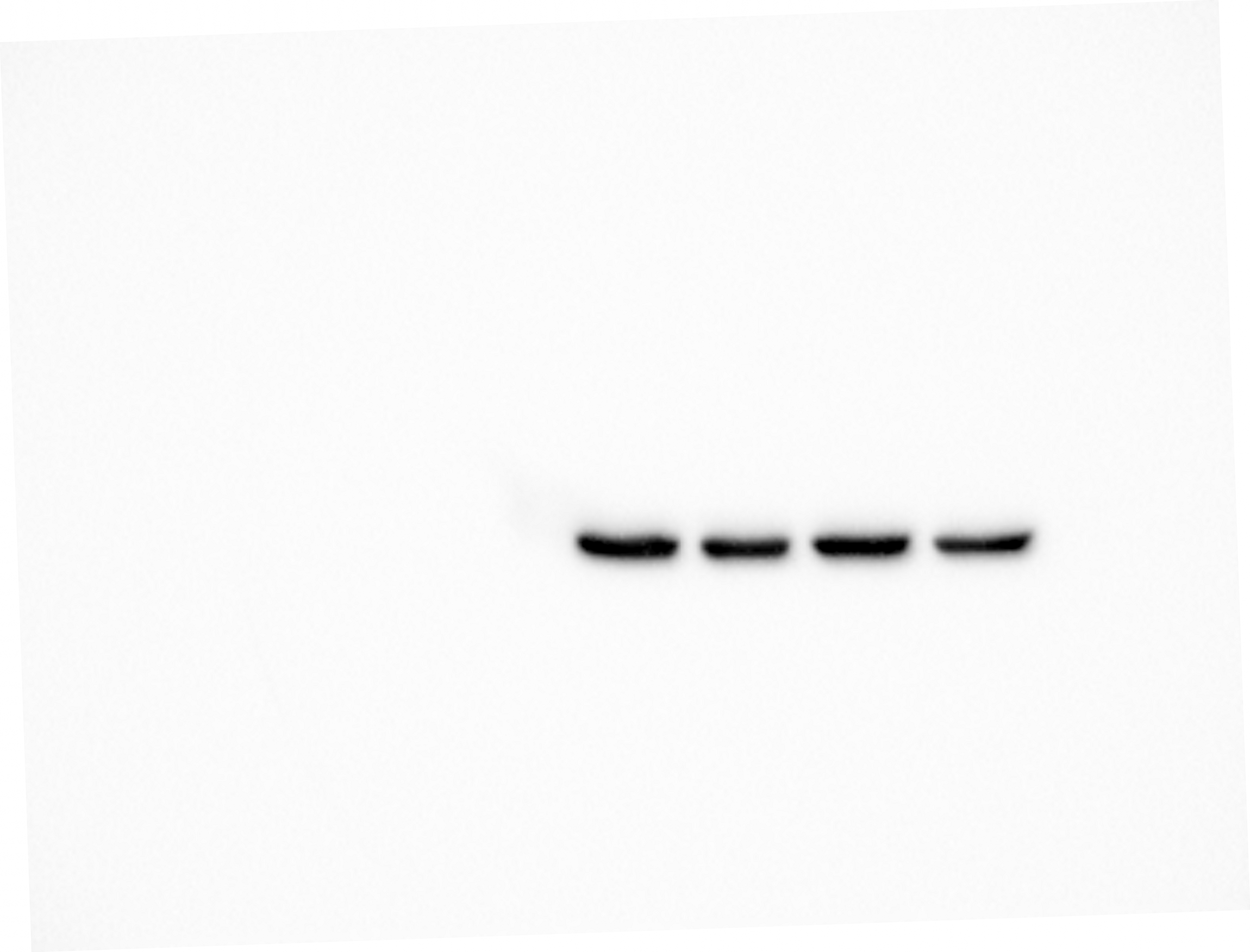

Supplement: Supplementary file 1 [file DataSheet_1.zip › 1J.tif]

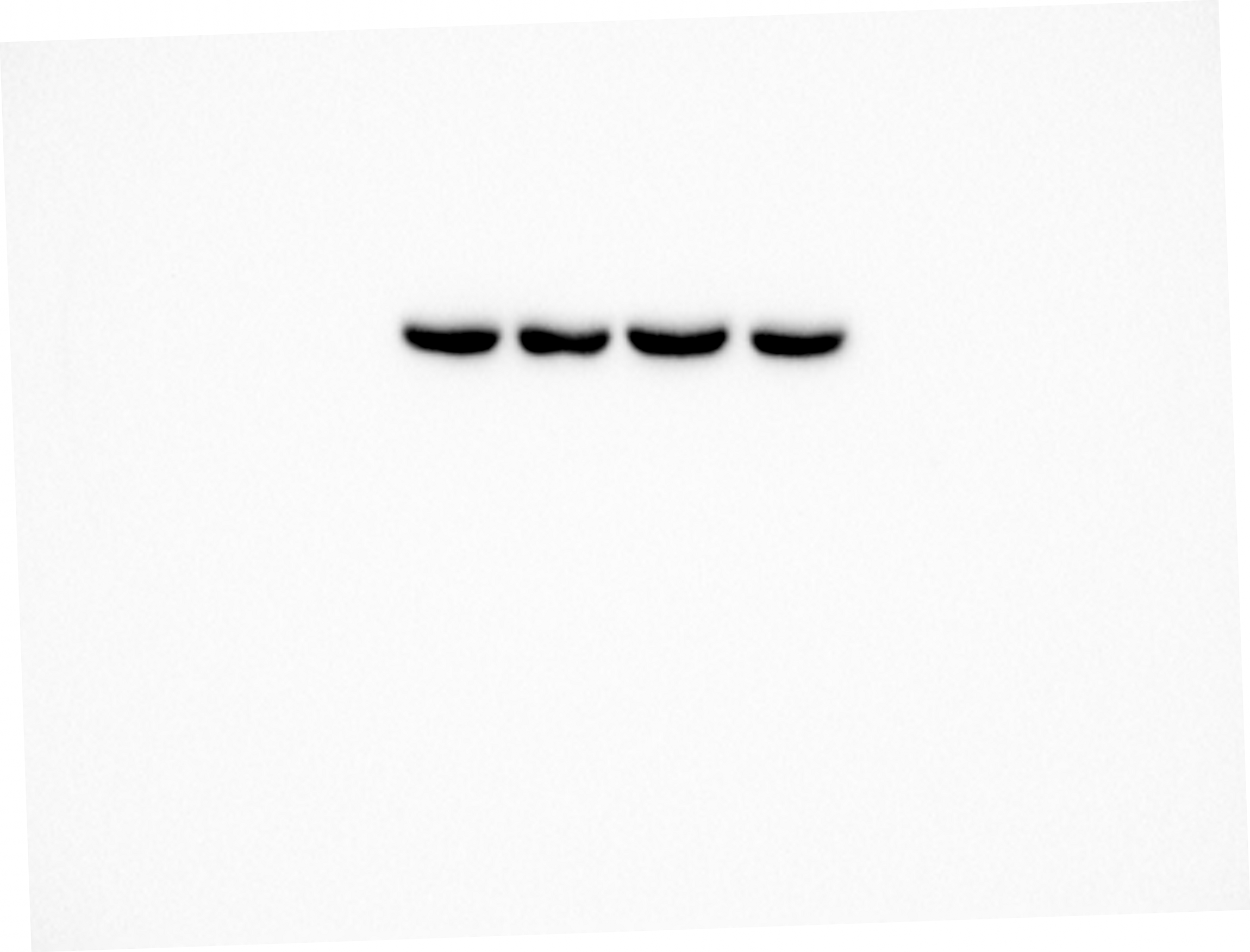

Supplement: Supplementary file 1 [file DataSheet_1.zip › 1K.tif]

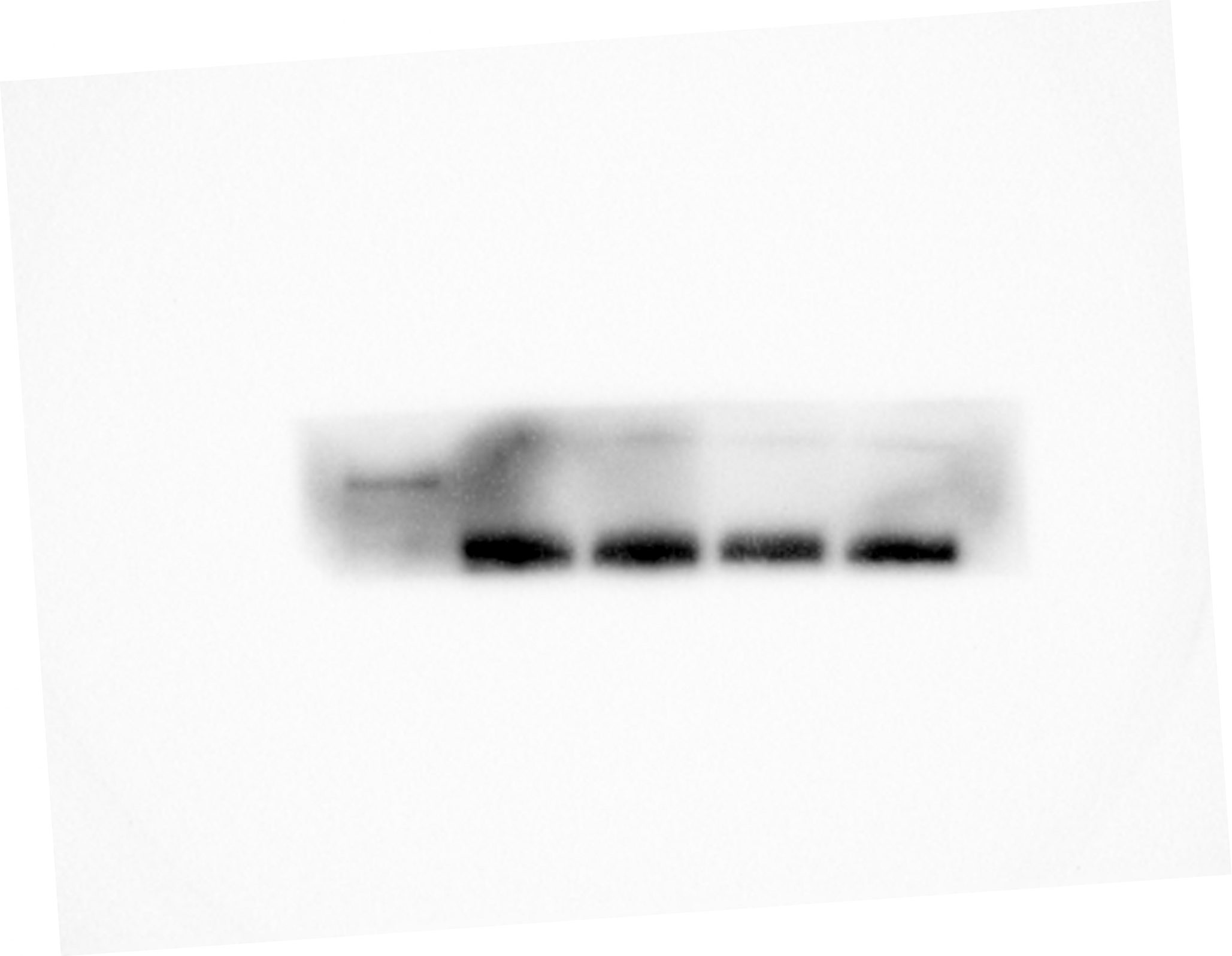

Supplement: Supplementary file 2 [file DataSheet_2.zip › 2B.tif]

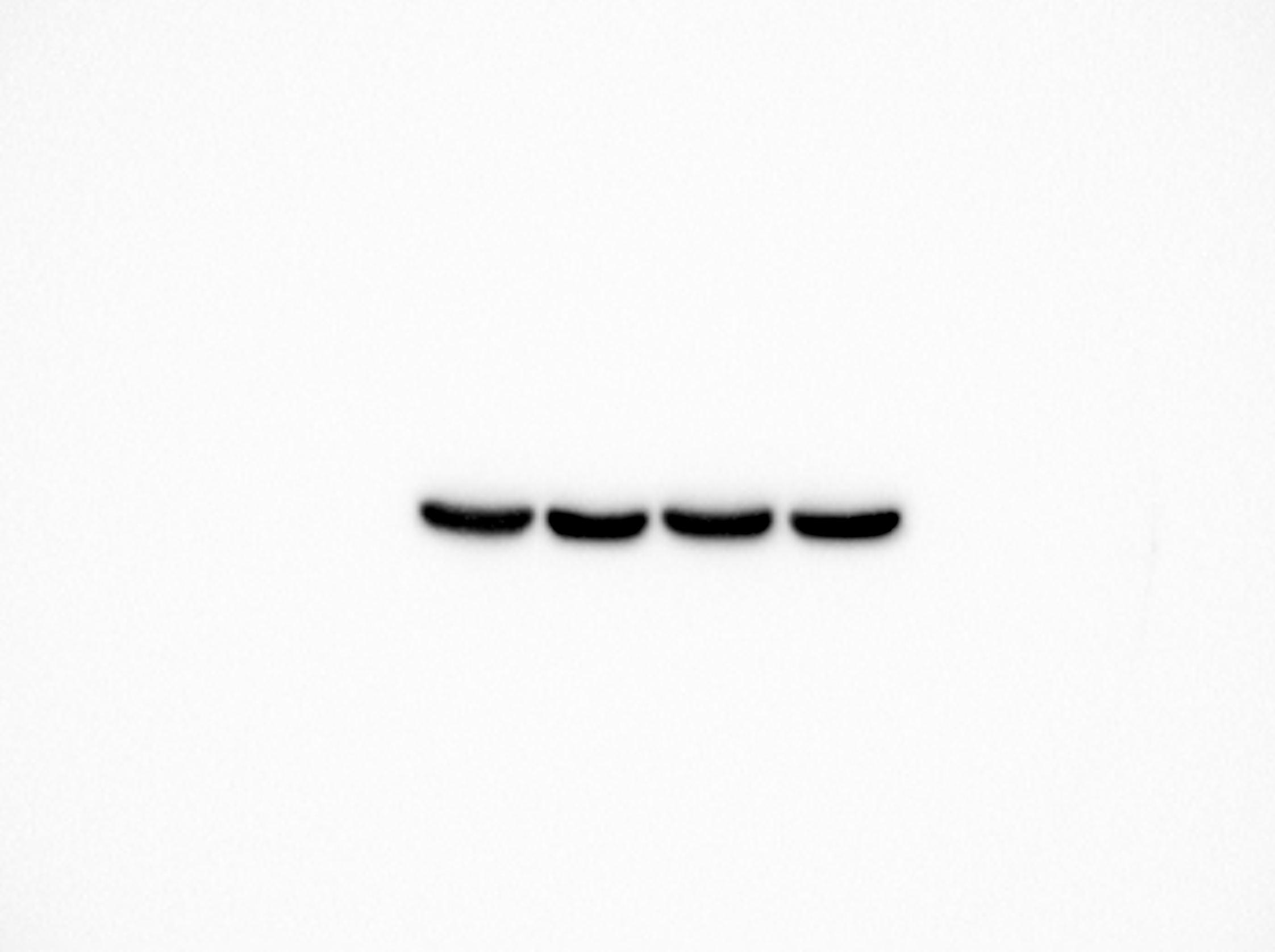

Supplement: Supplementary file 2 [file DataSheet_2.zip › 2C.tif]

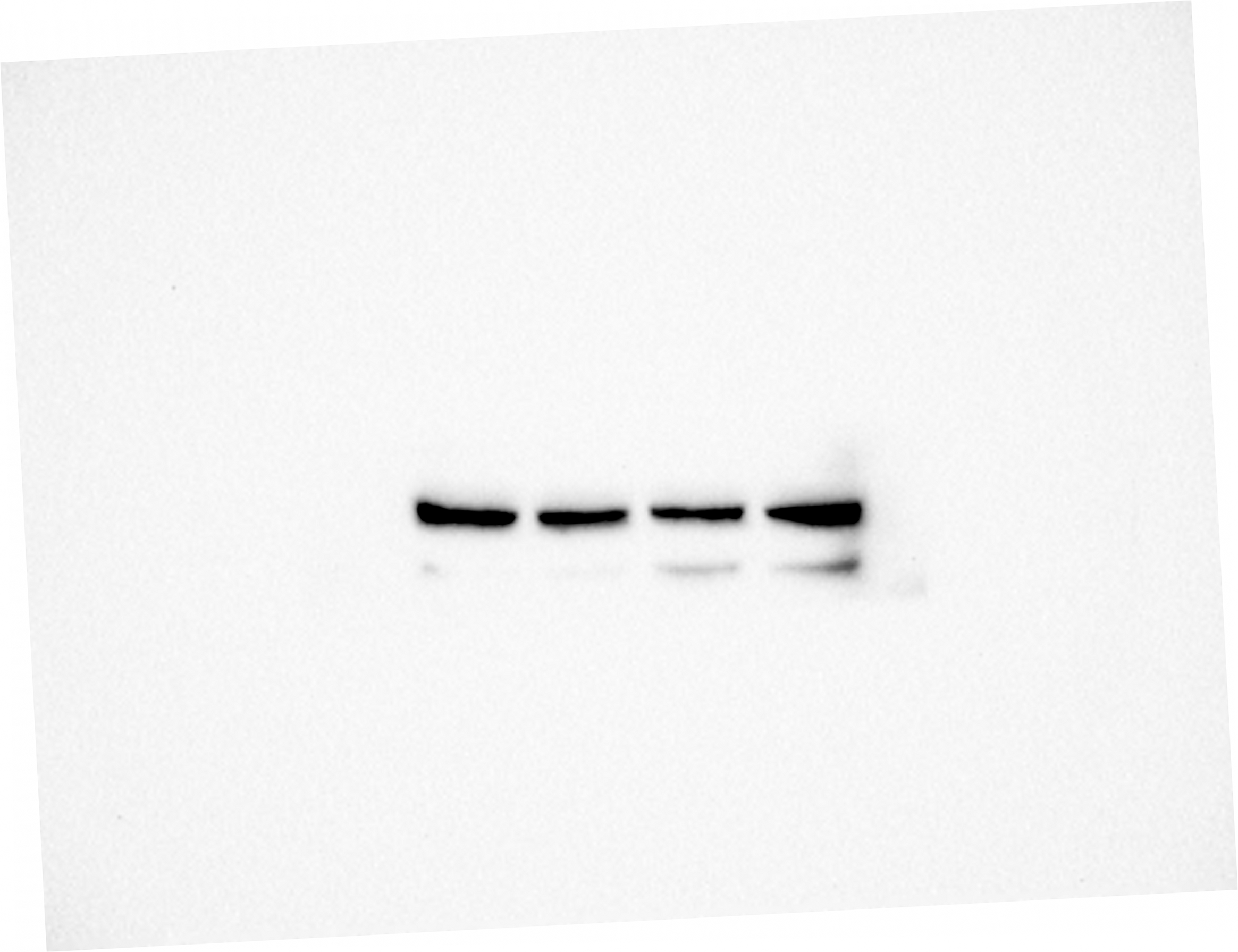

Supplement: Supplementary file 2 [file DataSheet_2.zip › 2D.tif]

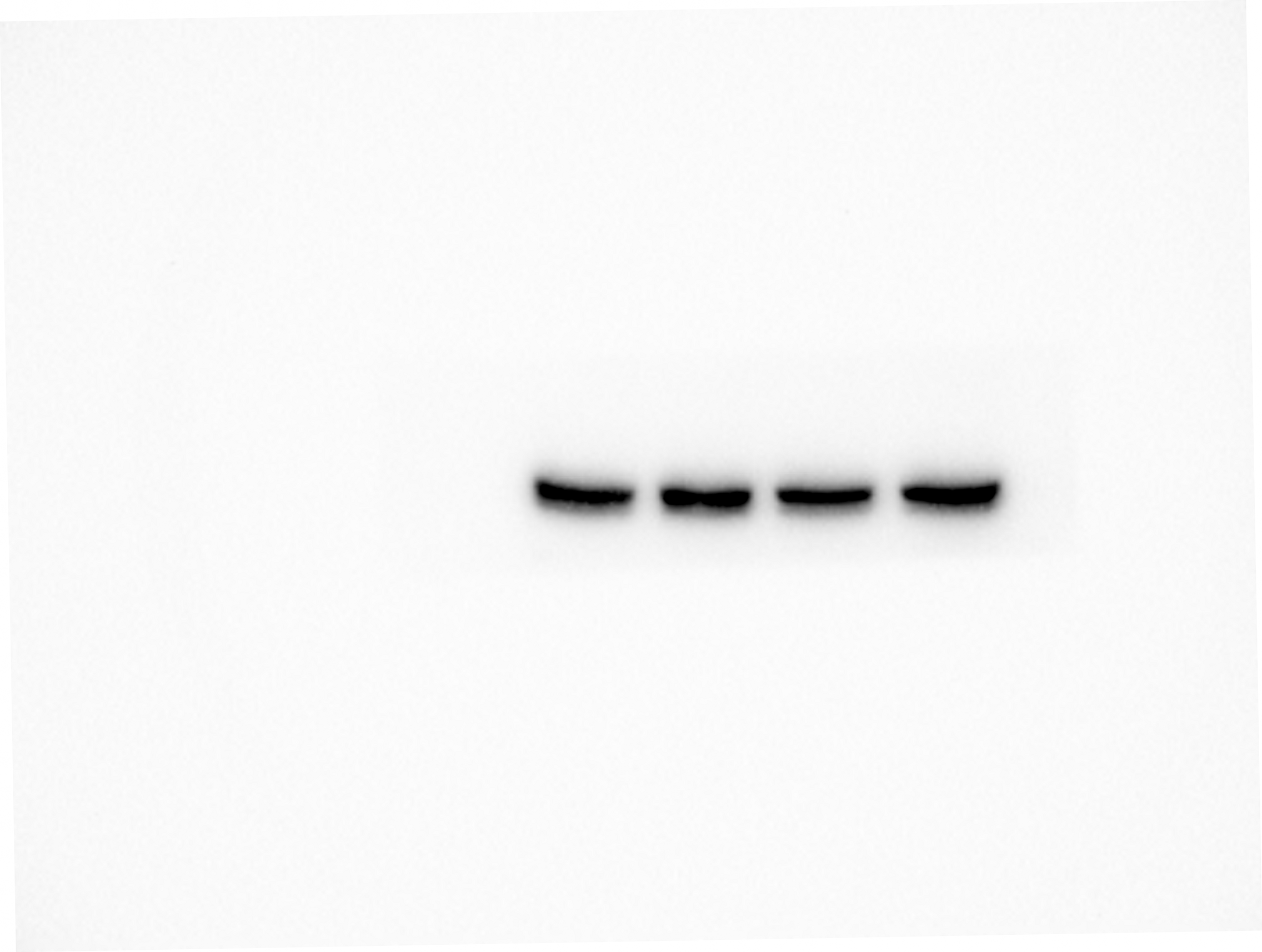

Supplement: Supplementary file 2 [file DataSheet_2.zip › 2E.tif]

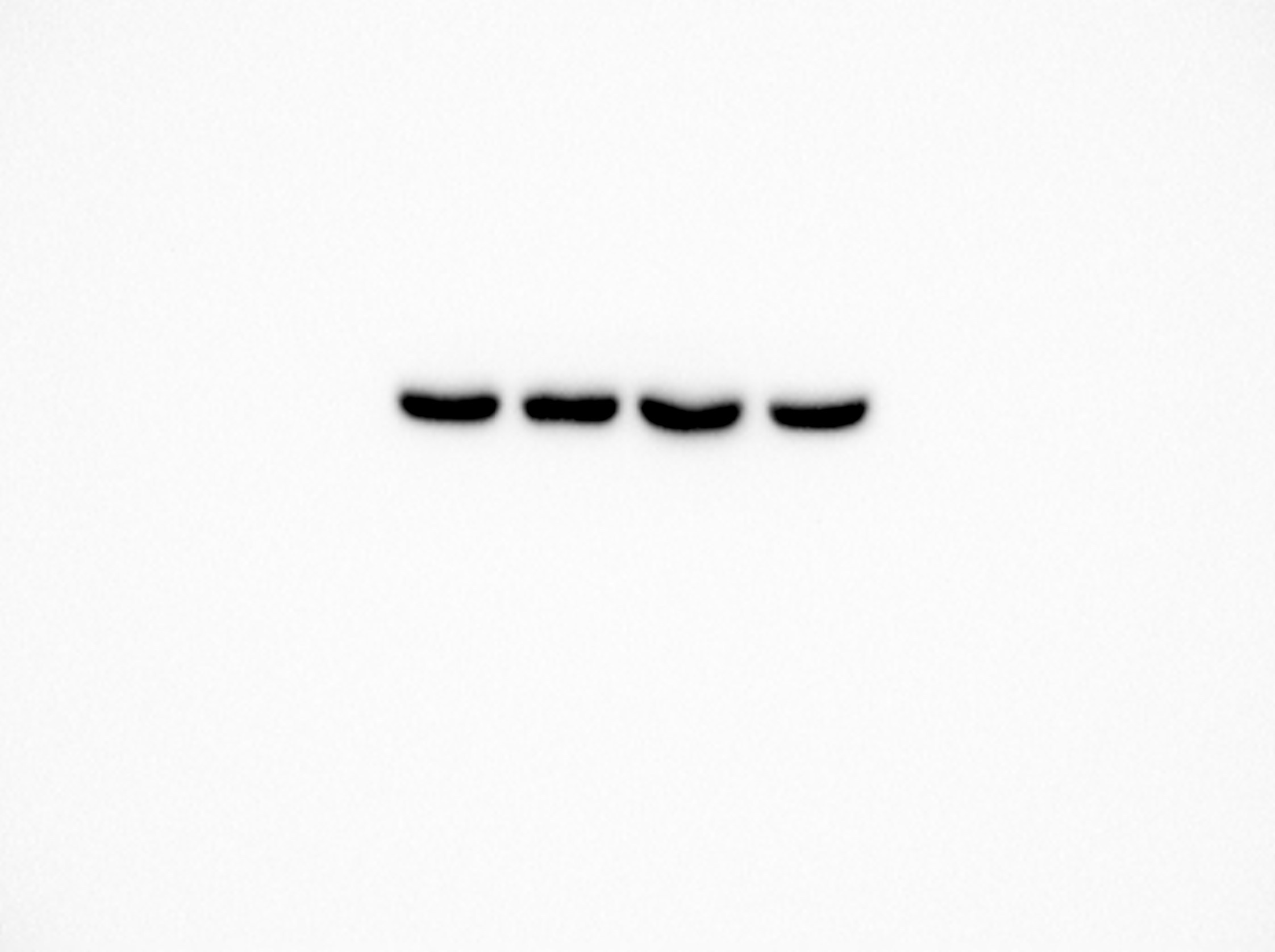

Supplement: Supplementary file 2 [file DataSheet_2.zip › 2F.tif]

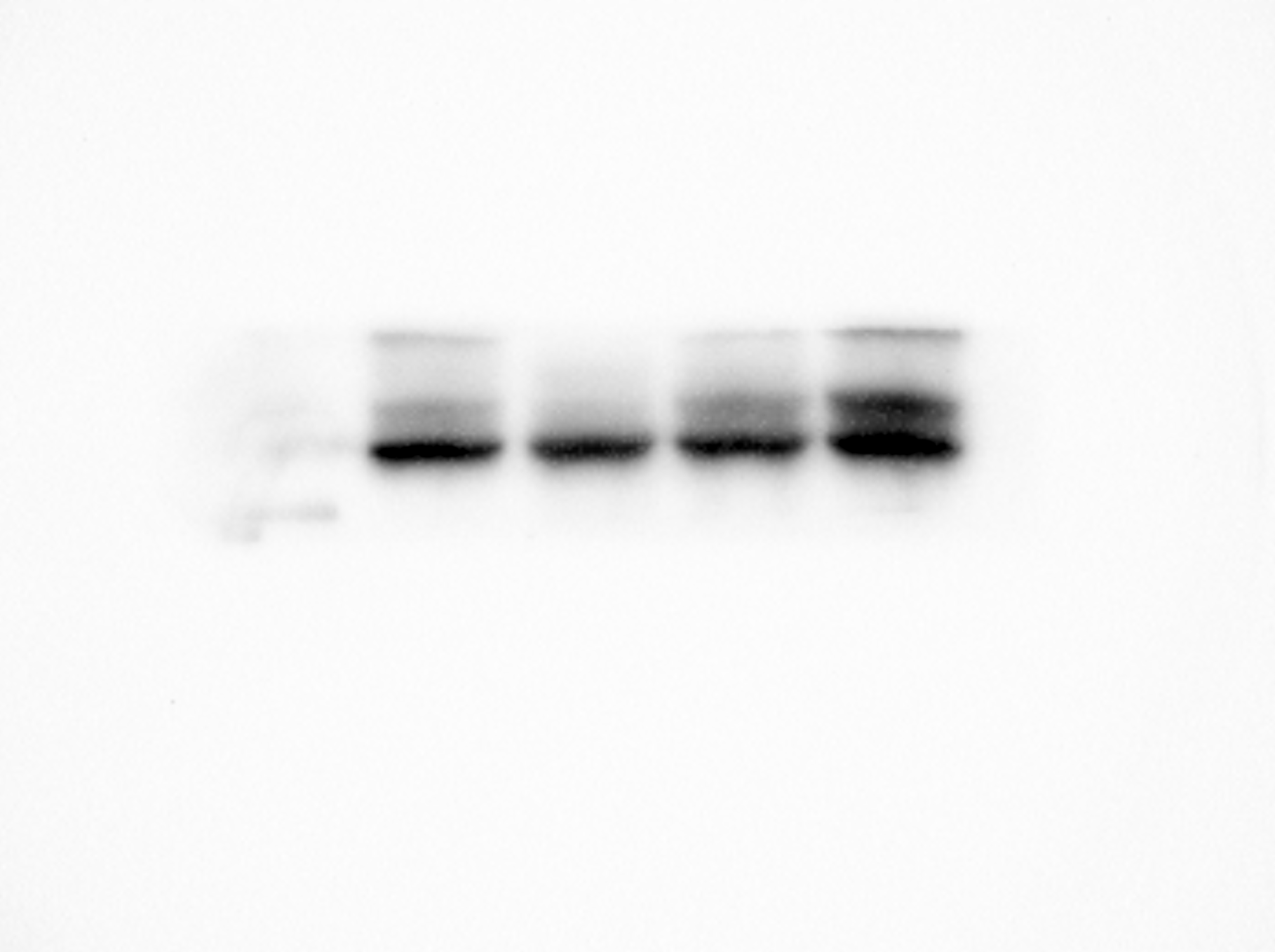

Supplement: Supplementary file 2 [file DataSheet_2.zip › 2G.tif]

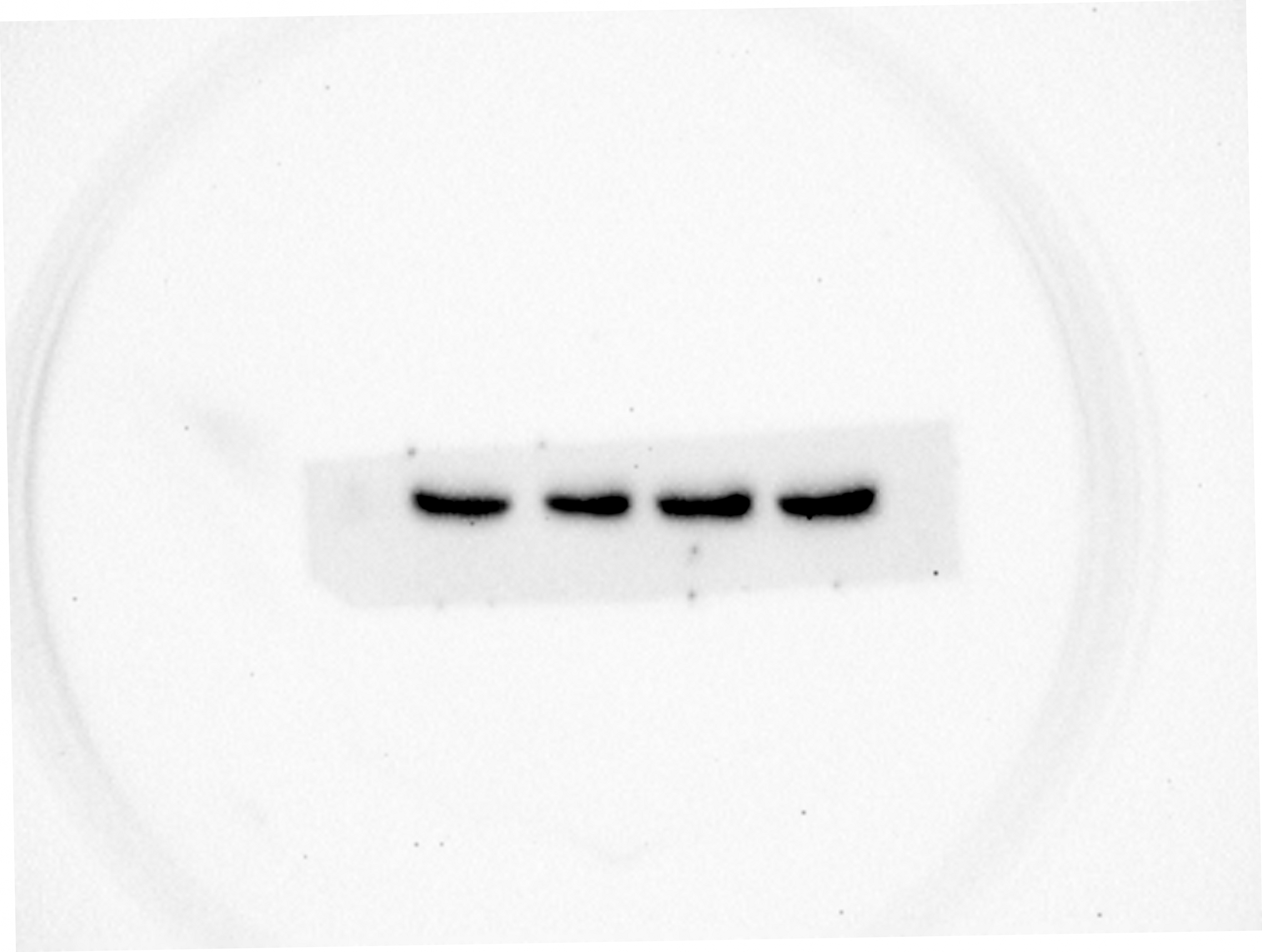

Supplement: Supplementary file 2 [file DataSheet_2.zip › 2H.tif]

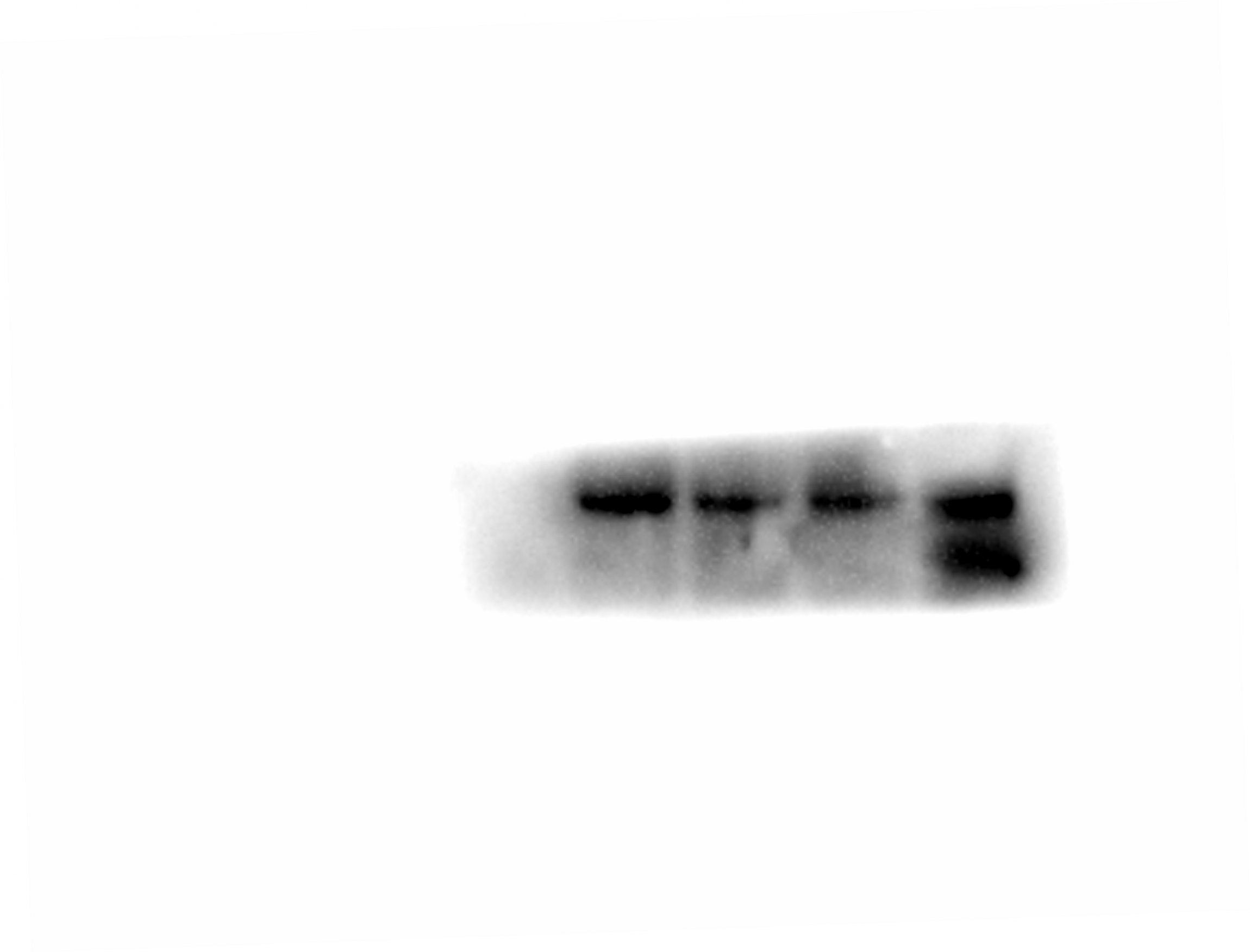

Supplement: Supplementary file 2 [file DataSheet_2.zip › 2I.tif]

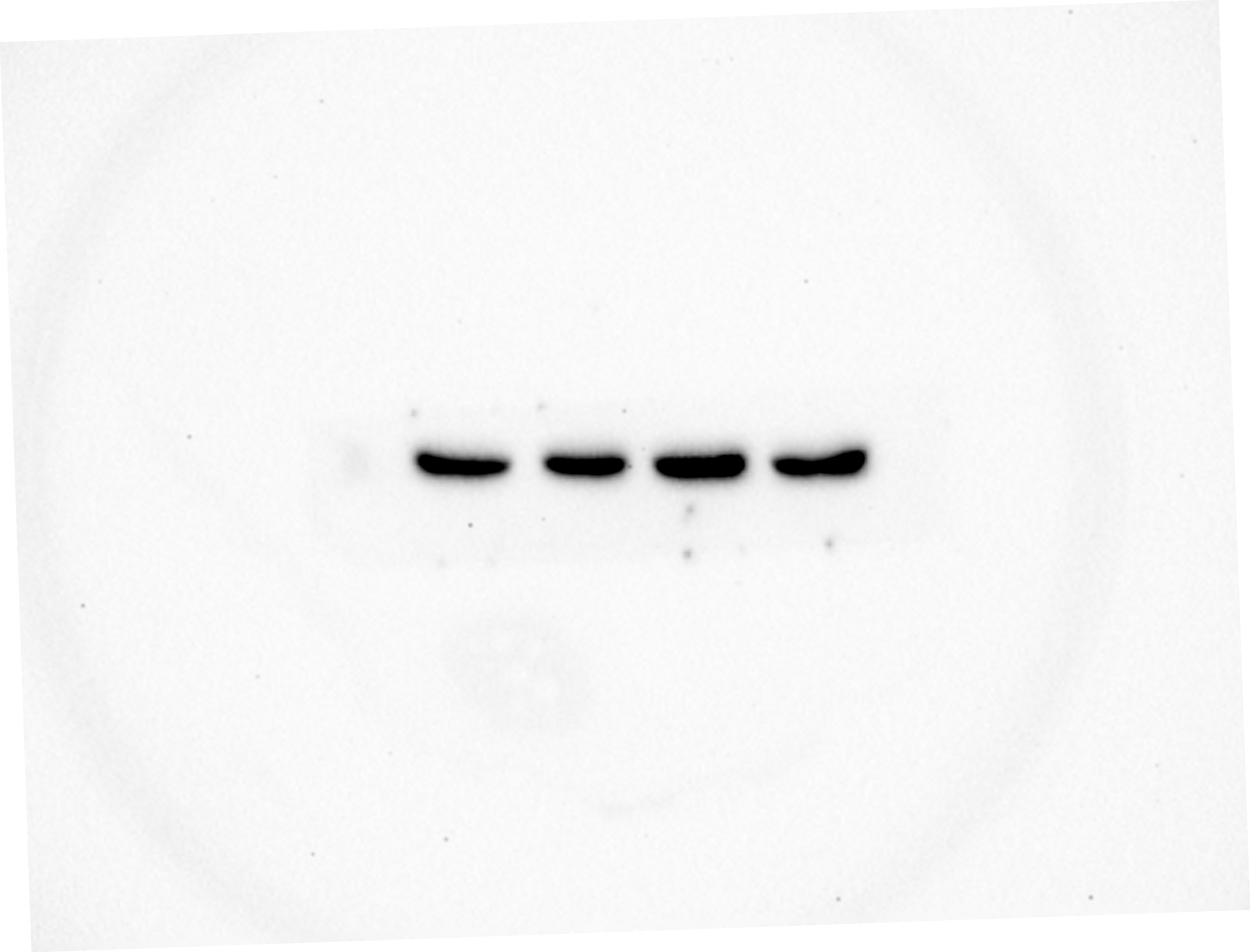

Supplement: Supplementary file 2 [file DataSheet_2.zip › 2J.tif]

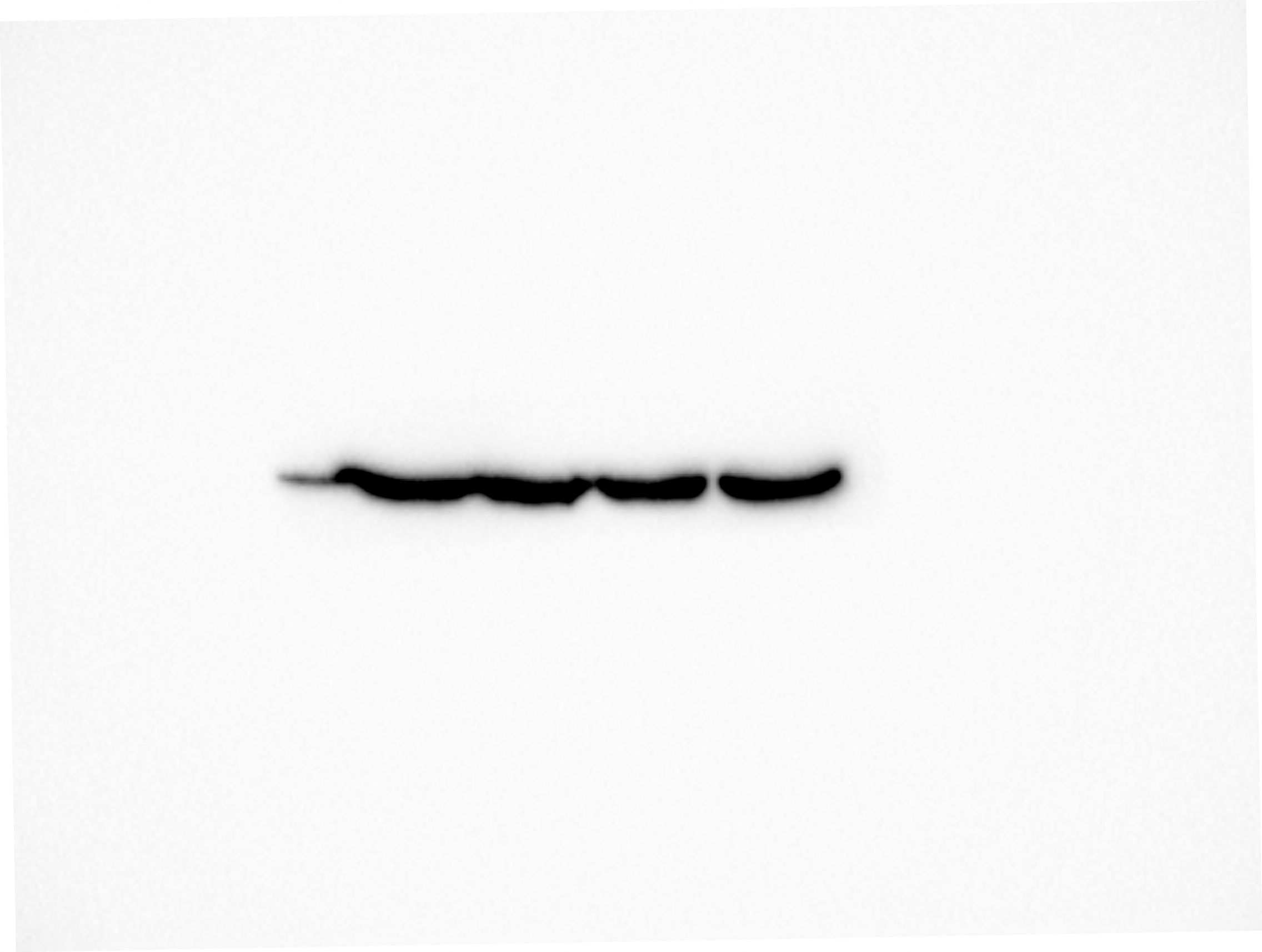

Supplement: Supplementary file 2 [file DataSheet_2.zip › 2K.tif]

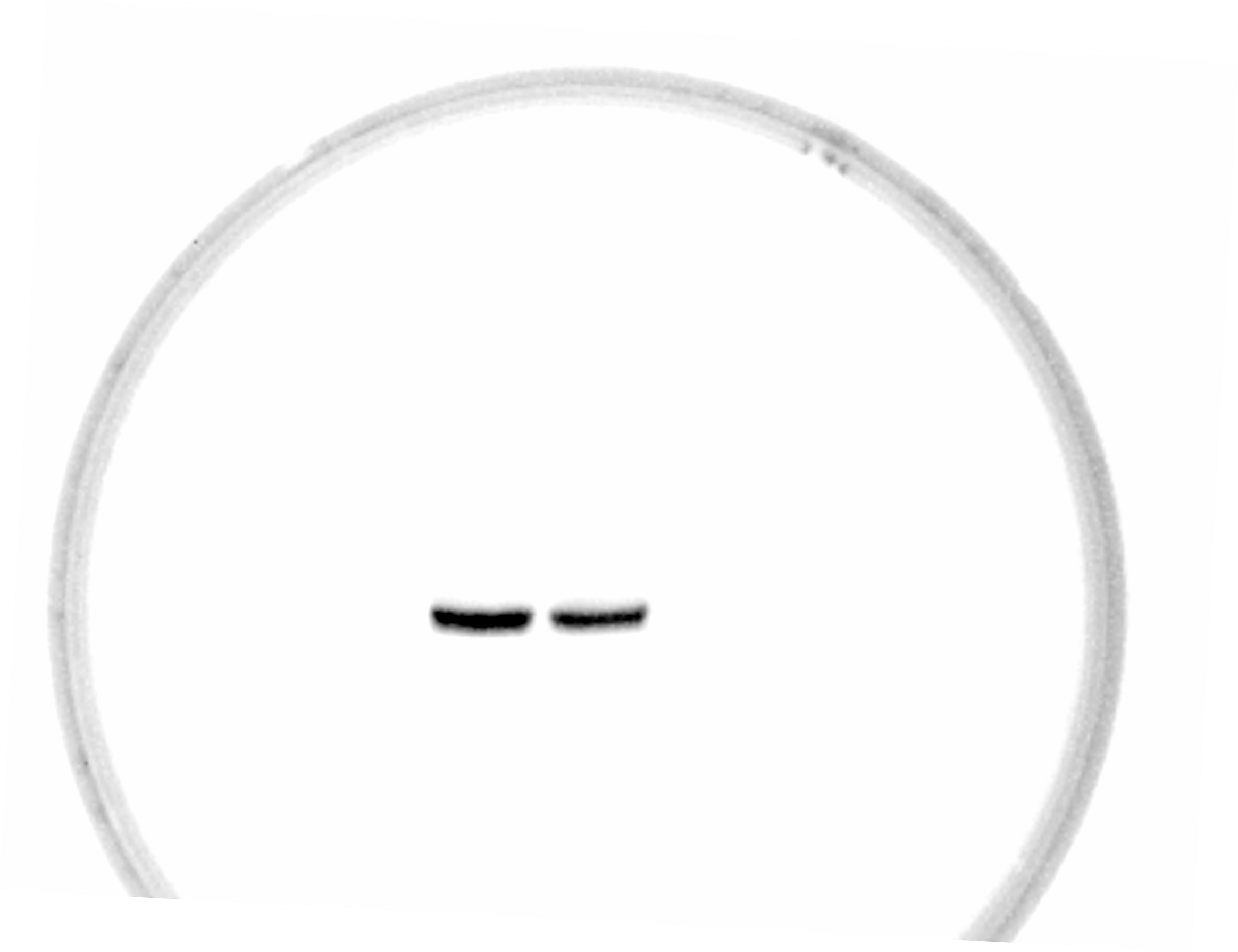

Supplement: Supplementary file 3 [file DataSheet_3.zip › 3A.tif]

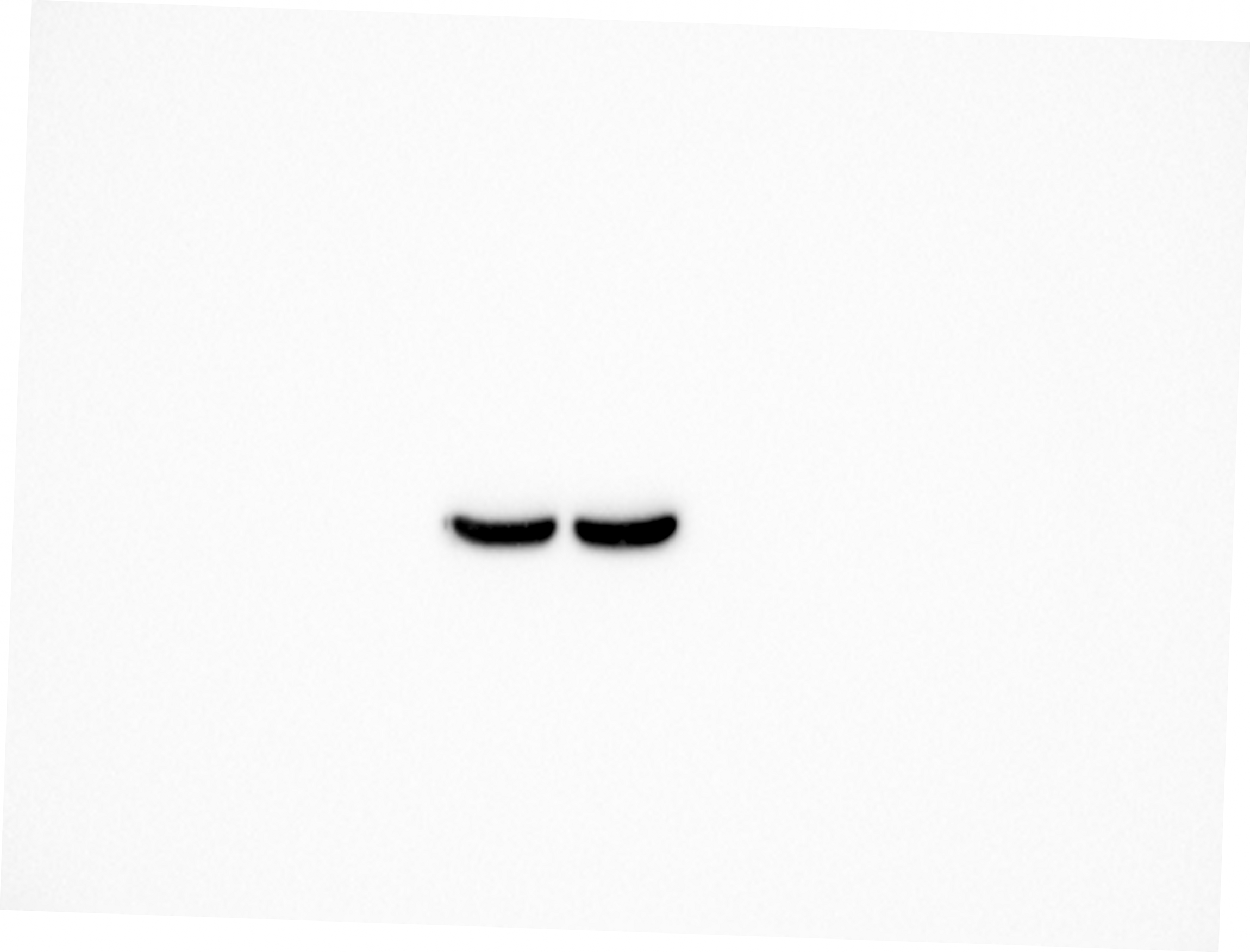

Supplement: Supplementary file 3 [file DataSheet_3.zip › 3B.tif]

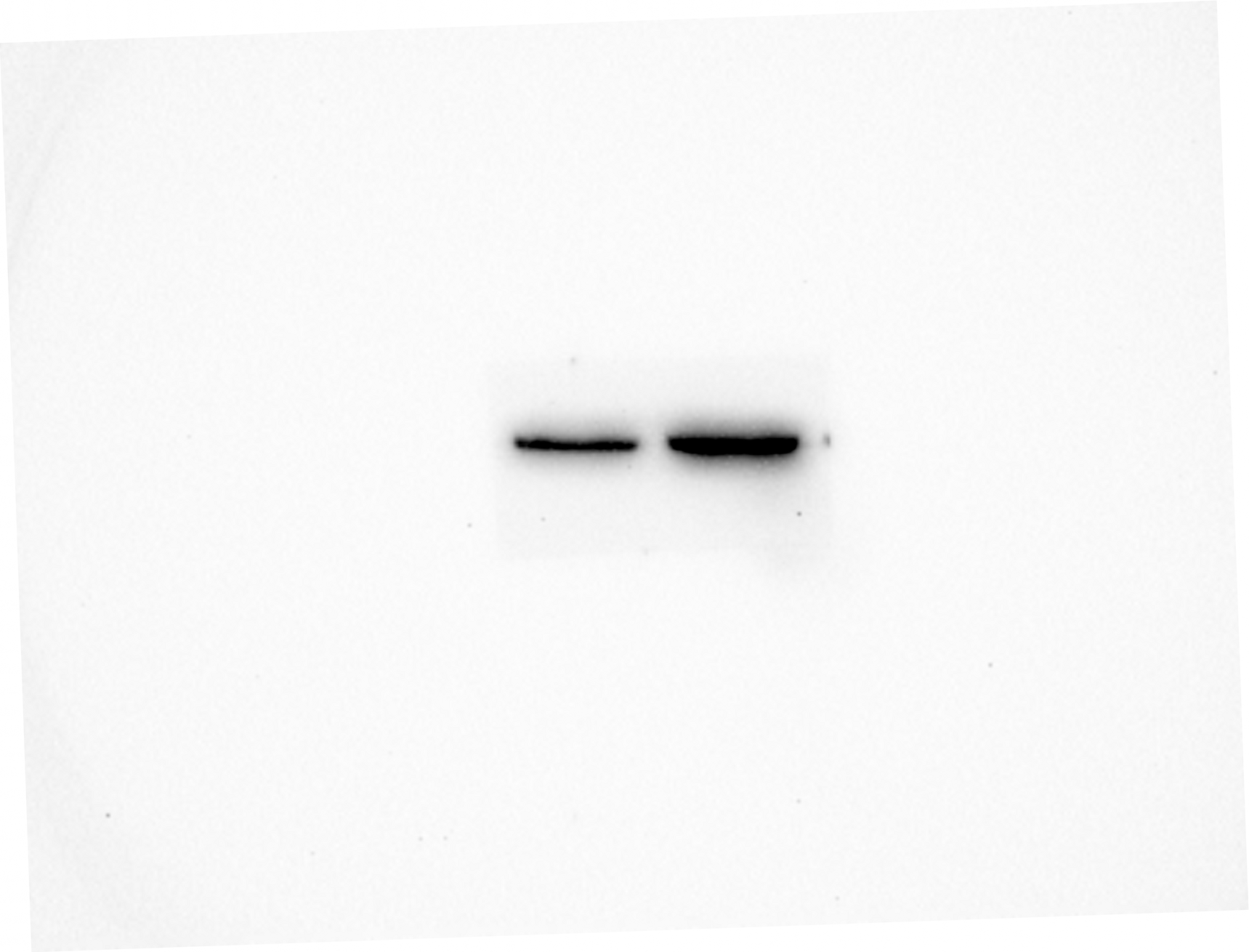

Supplement: Supplementary file 3 [file DataSheet_3.zip › 3C.tif]

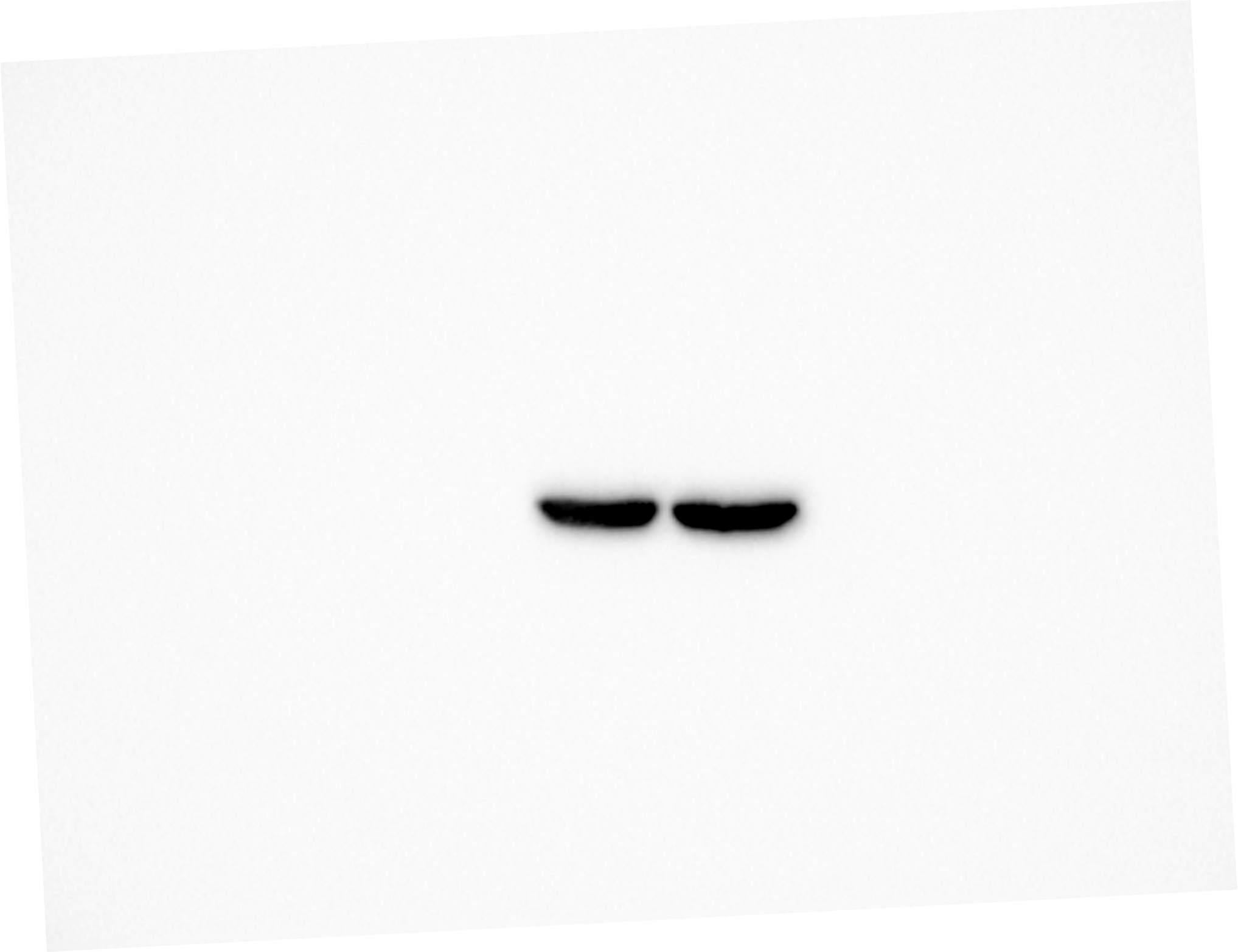

Supplement: Supplementary file 3 [file DataSheet_3.zip › 3D.tif]

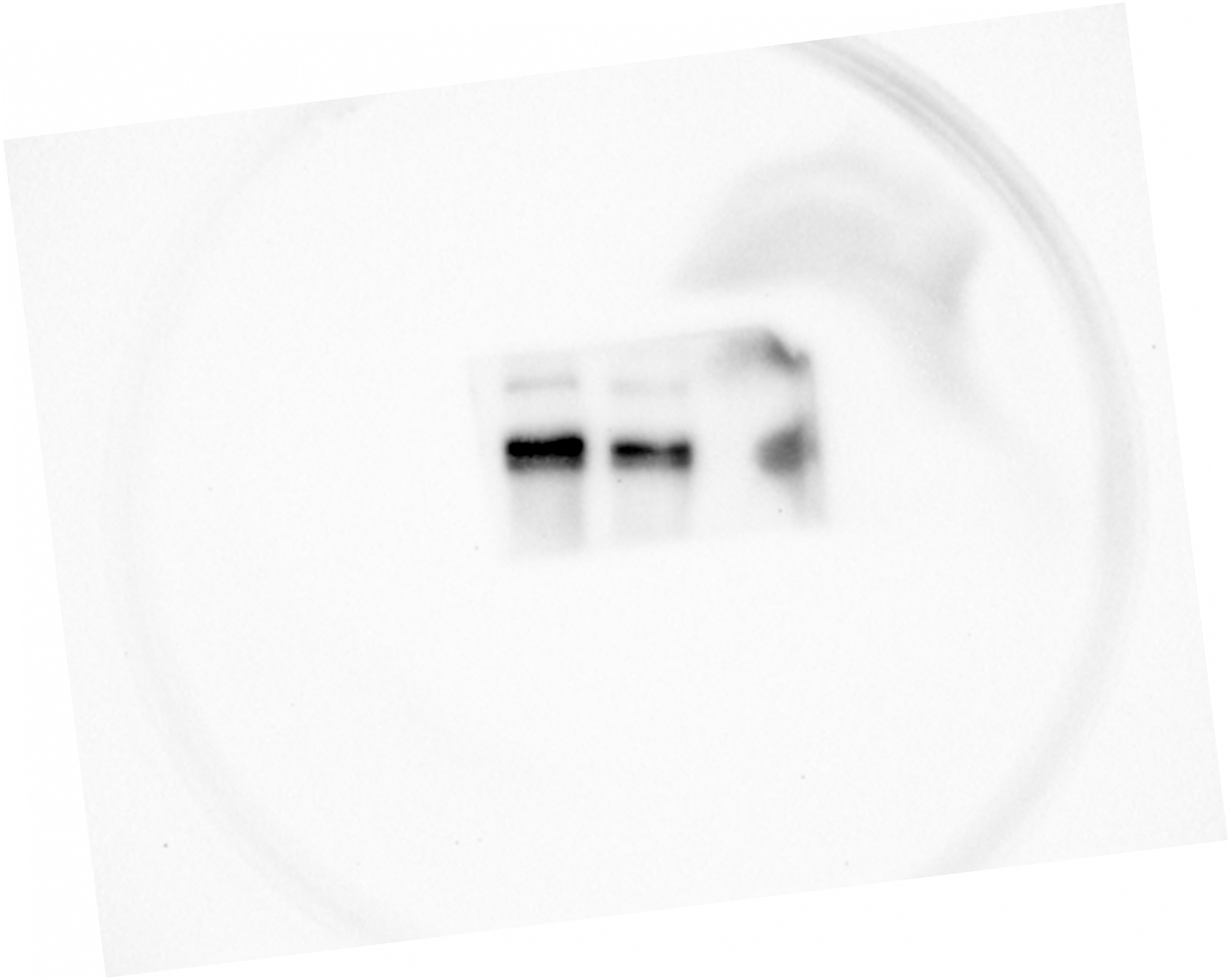

Supplement: Supplementary file 3 [file DataSheet_3.zip › 3E.tif]

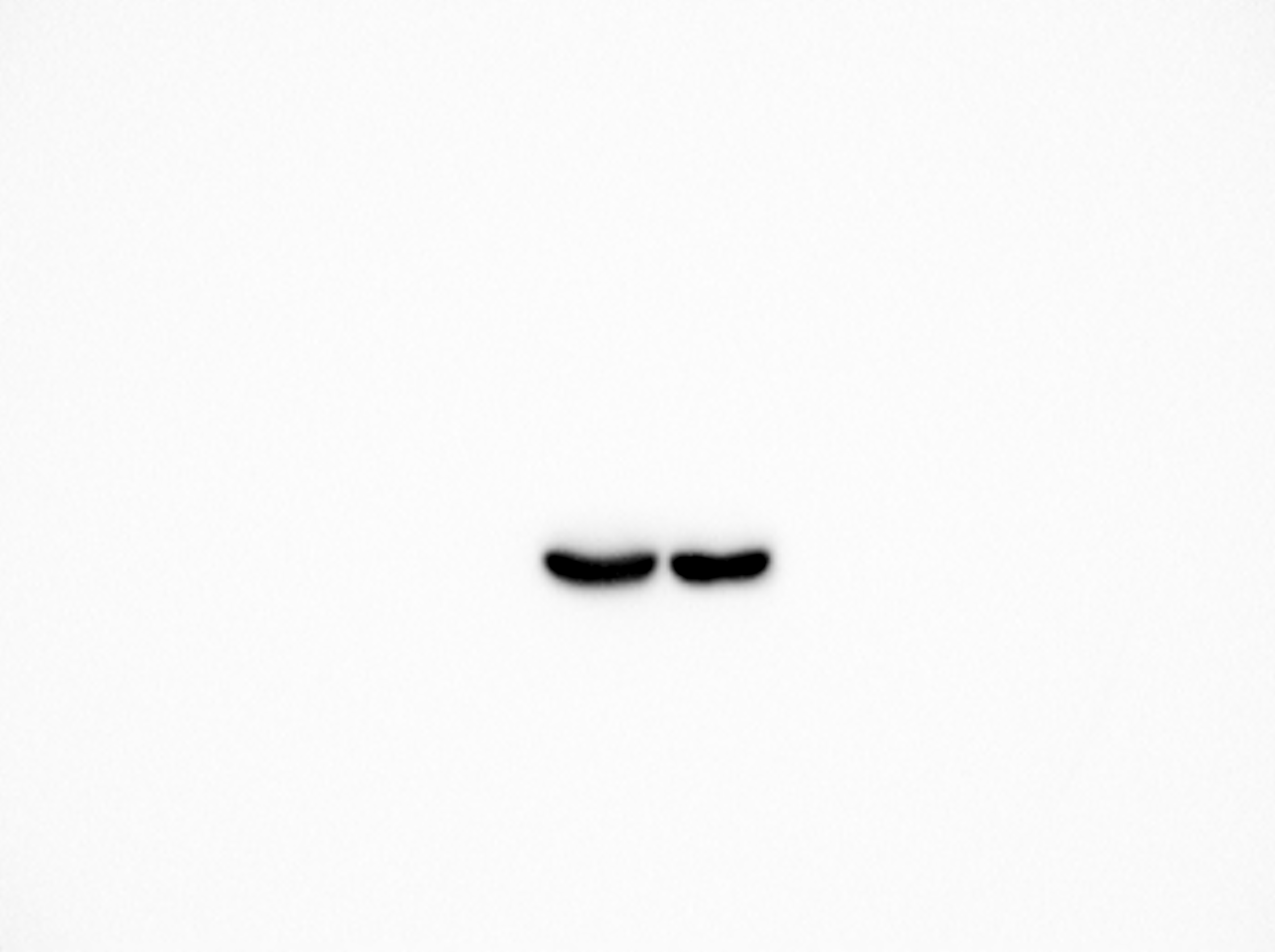

Supplement: Supplementary file 3 [file DataSheet_3.zip › 3F.tif]

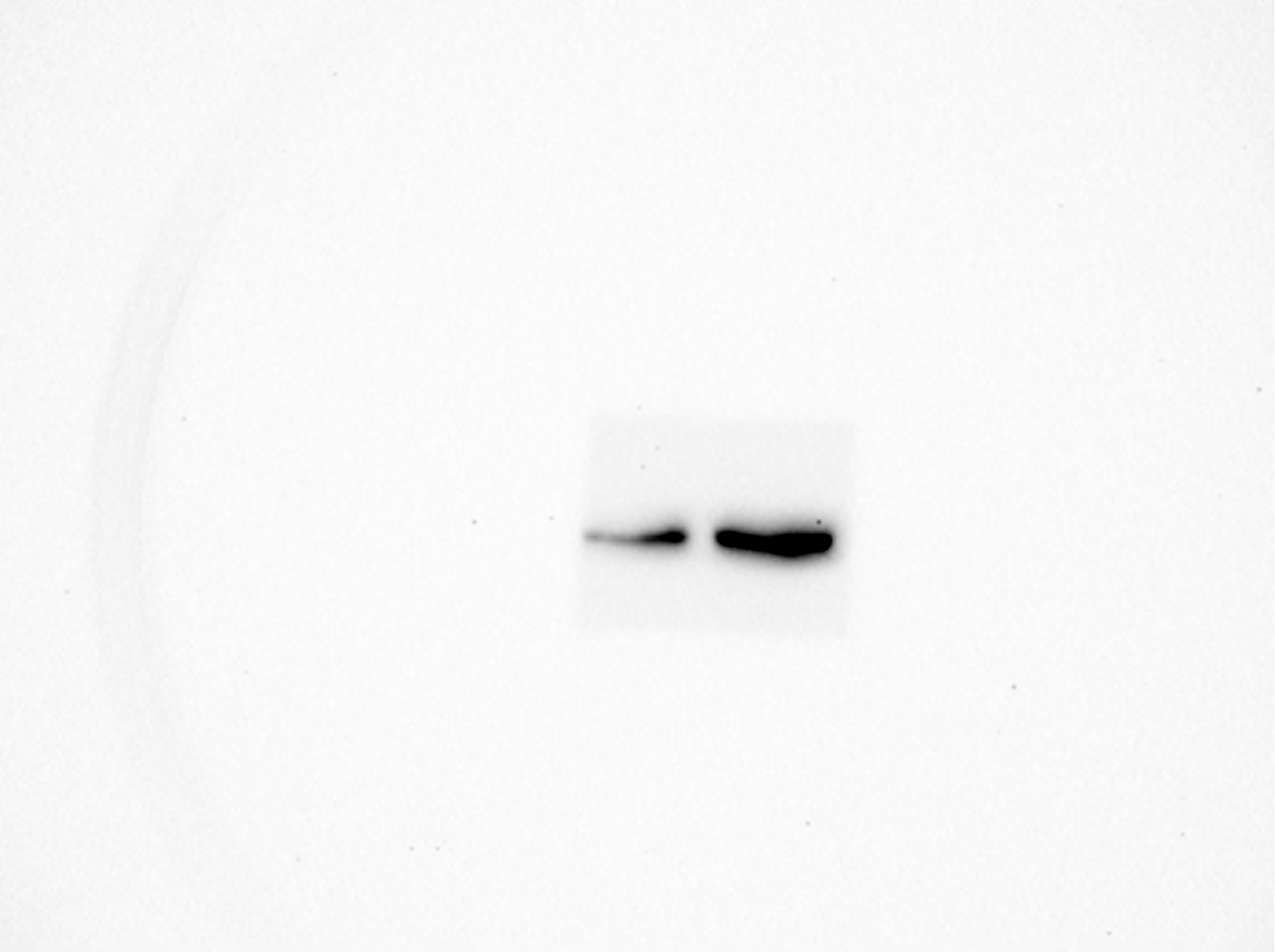

Supplement: Supplementary file 3 [file DataSheet_3.zip › 3G.tif]

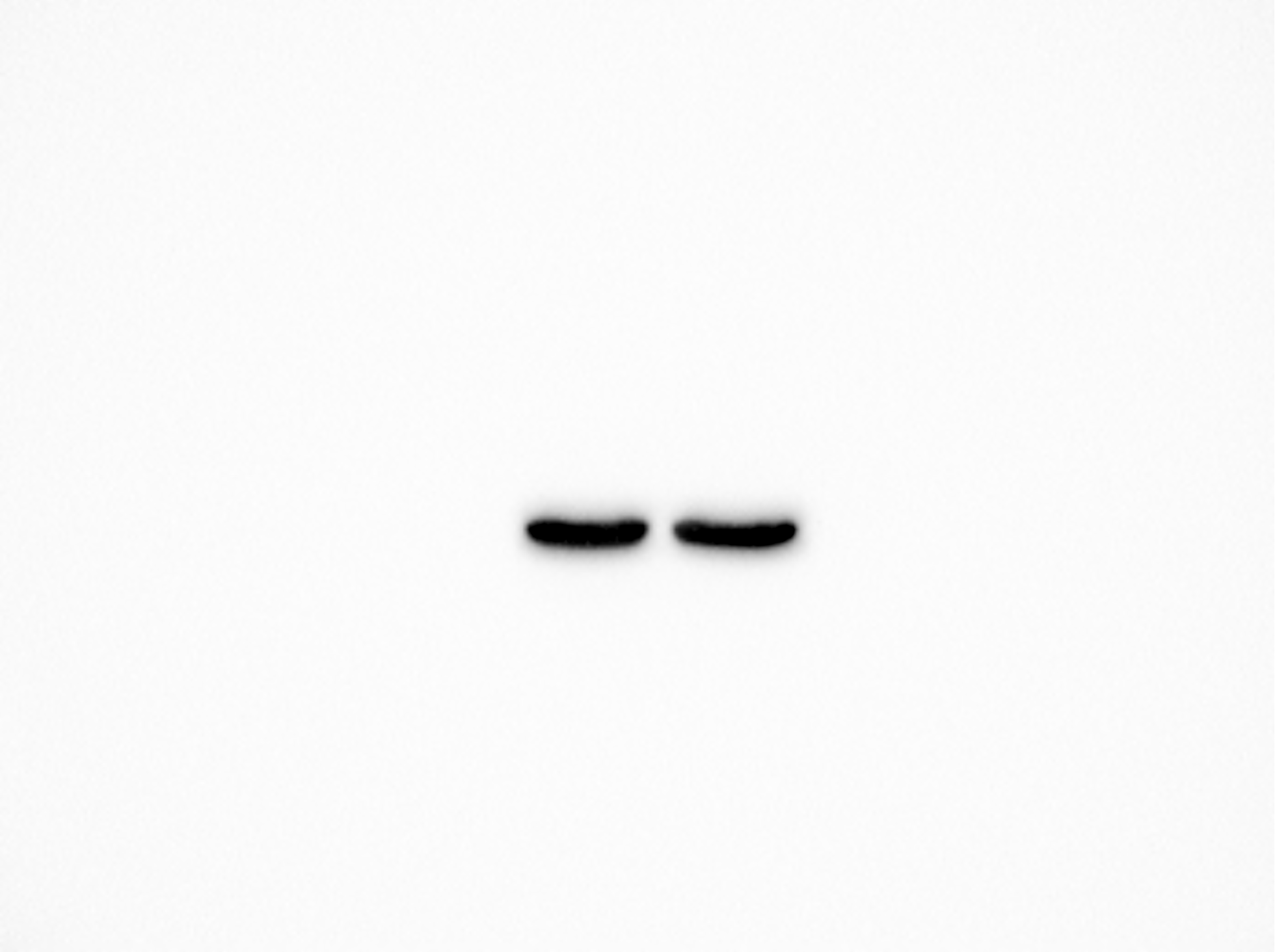

Supplement: Supplementary file 3 [file DataSheet_3.zip › 3H.tif]

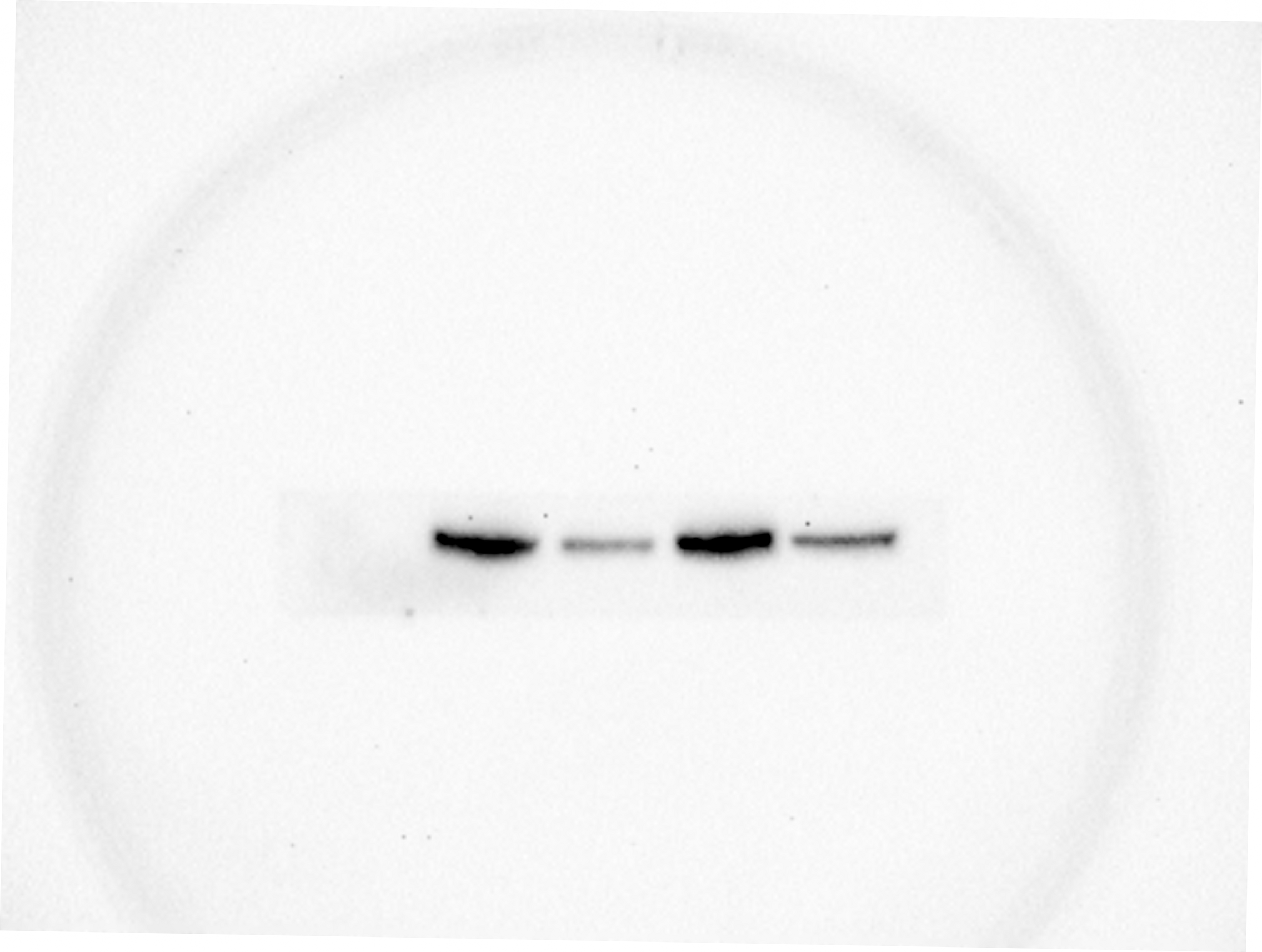

Supplement: Supplementary file 3 [file DataSheet_3.zip › 4A.tif]

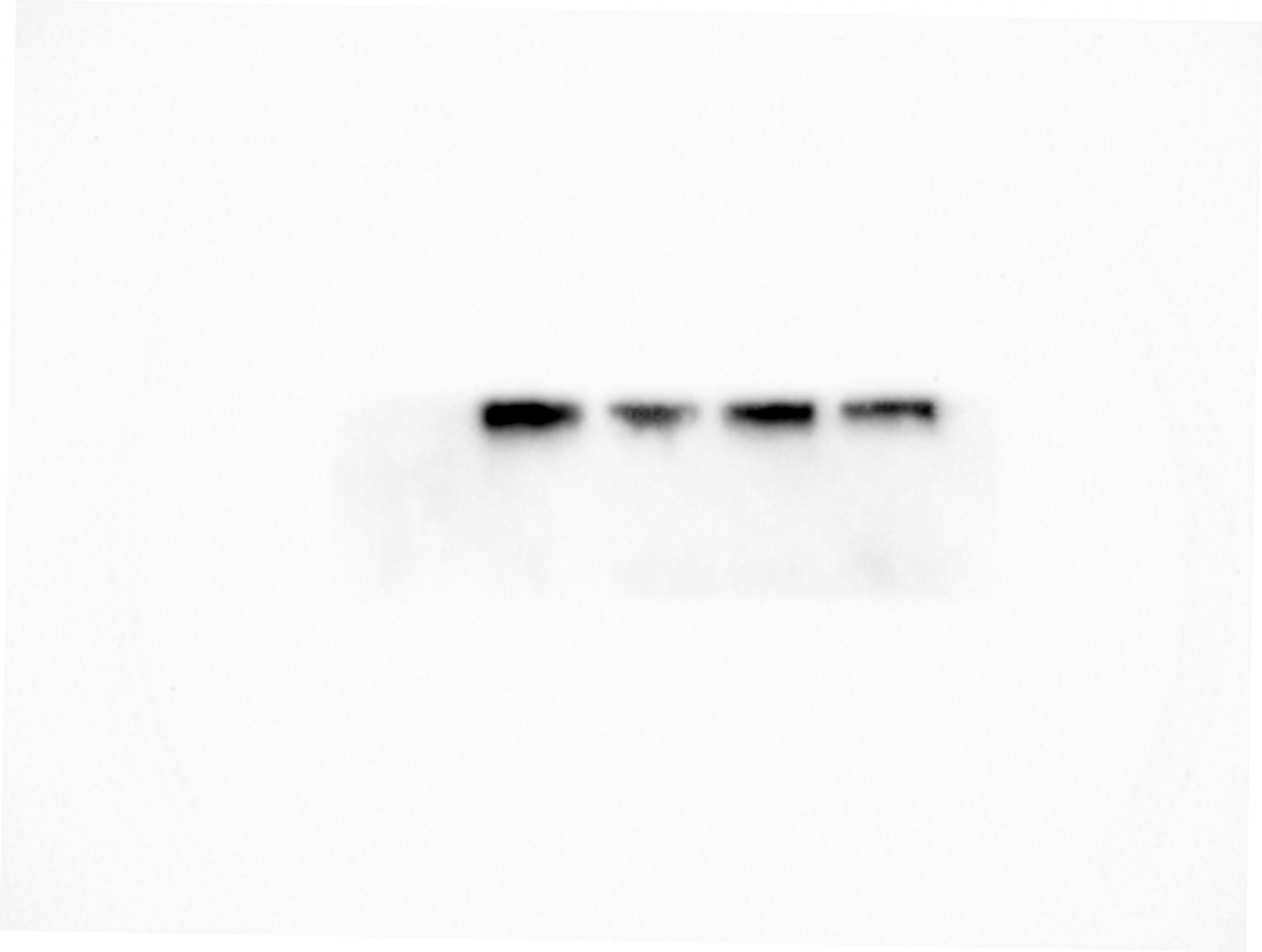

Supplement: Supplementary file 3 [file DataSheet_3.zip › 4B.tif]

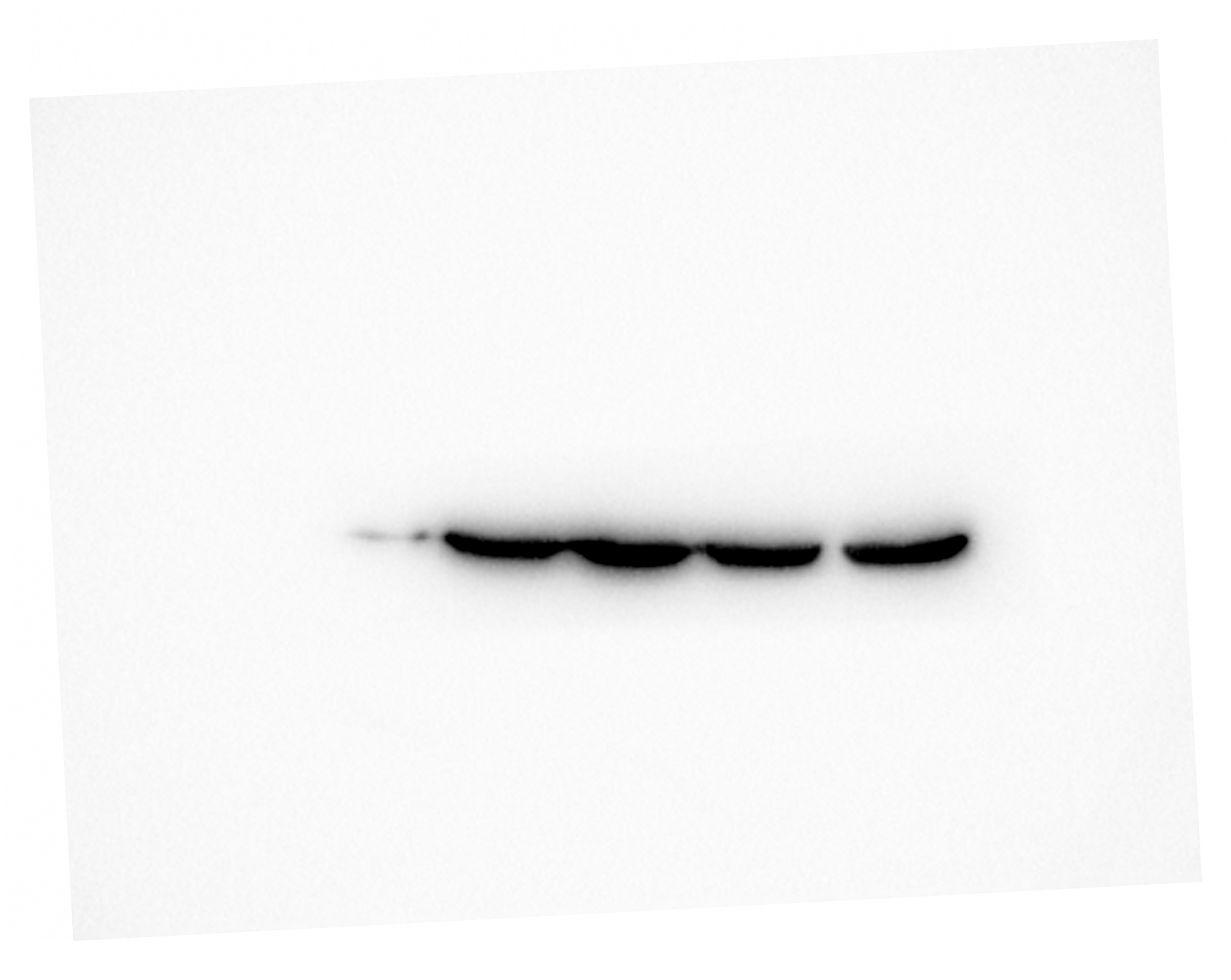

Supplement: Supplementary file 3 [file DataSheet_3.zip › 4C.tif]

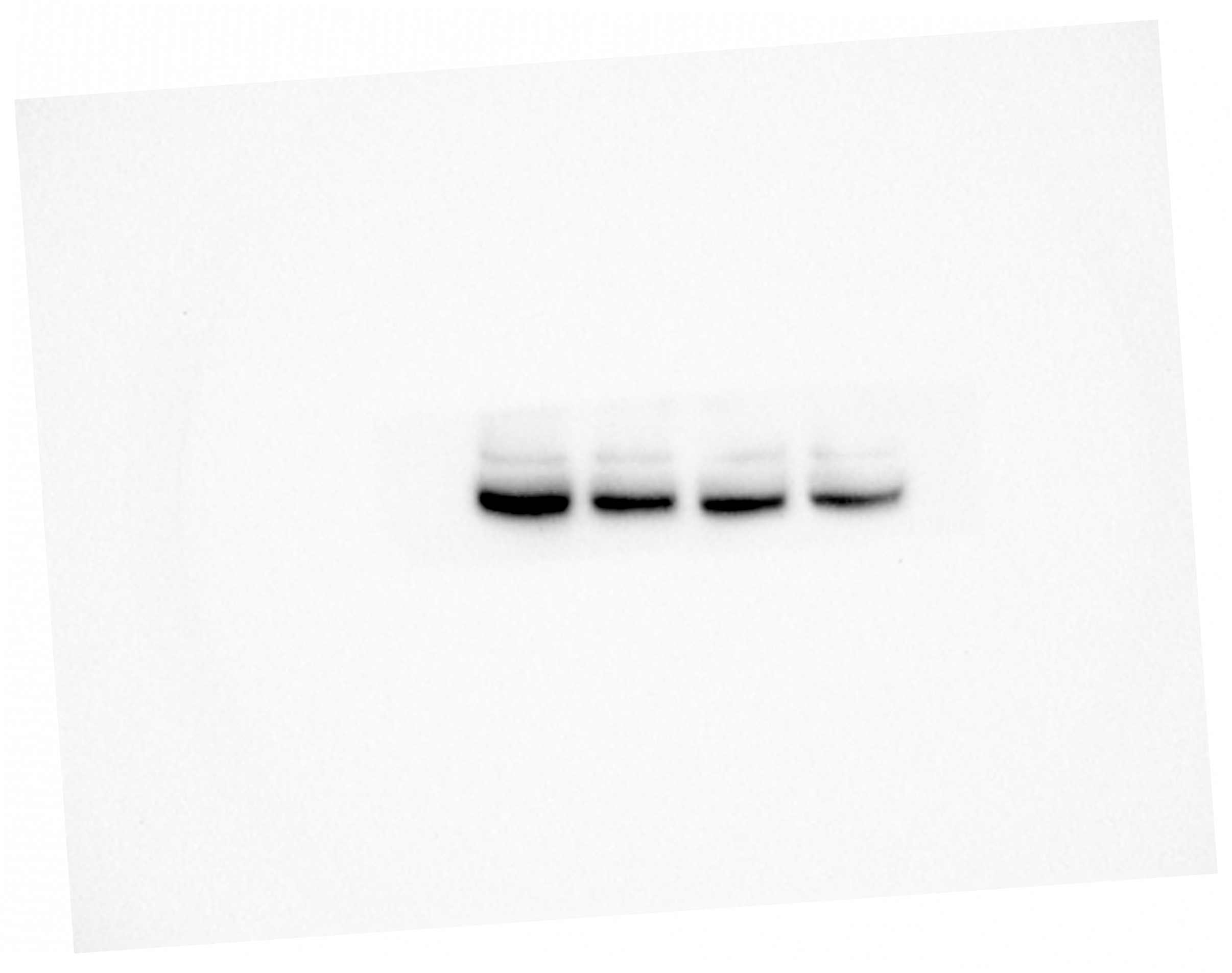

Supplement: Supplementary file 3 [file DataSheet_3.zip › 4D.tif]

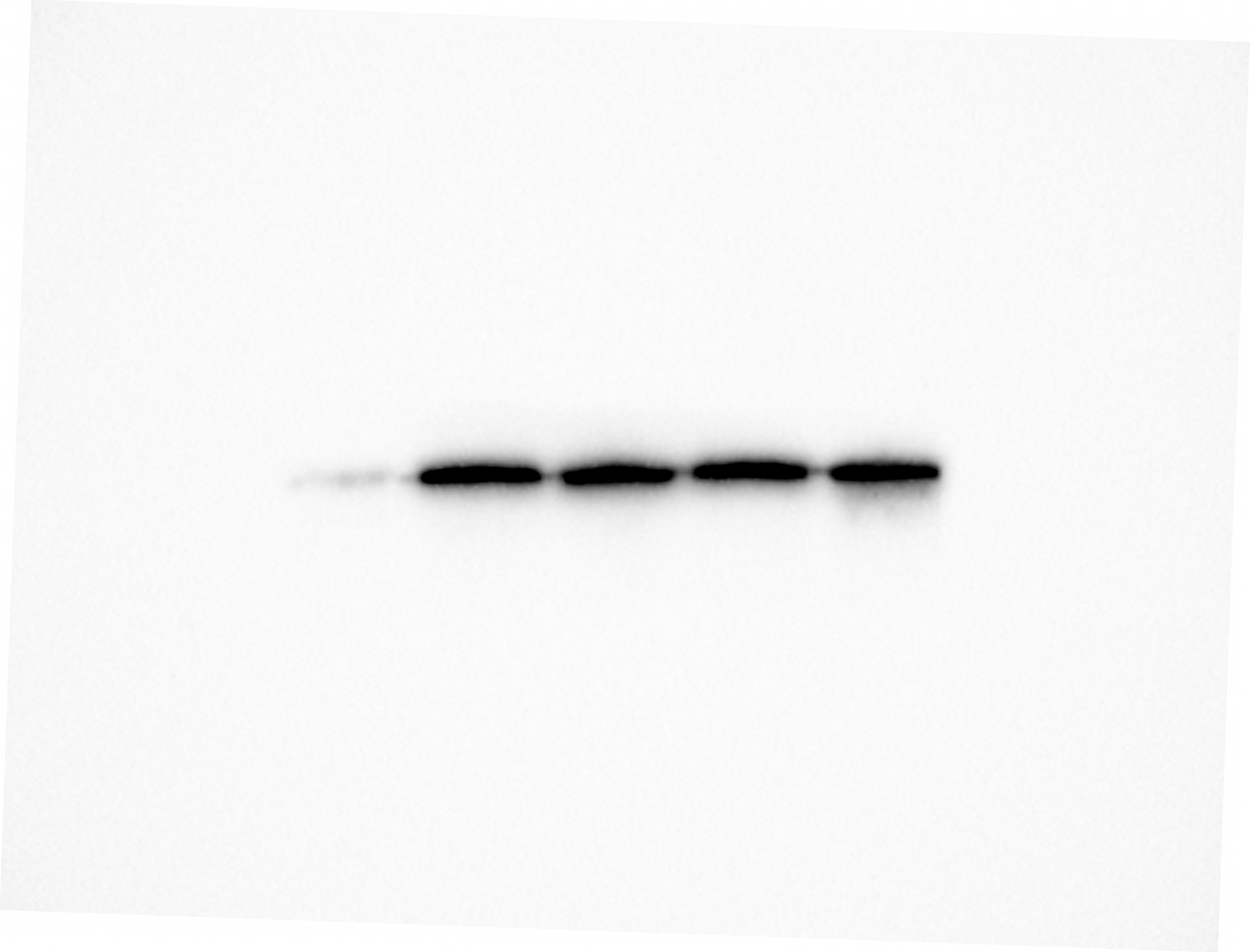

Supplement: Supplementary file 3 [file DataSheet_3.zip › 4E.tif]

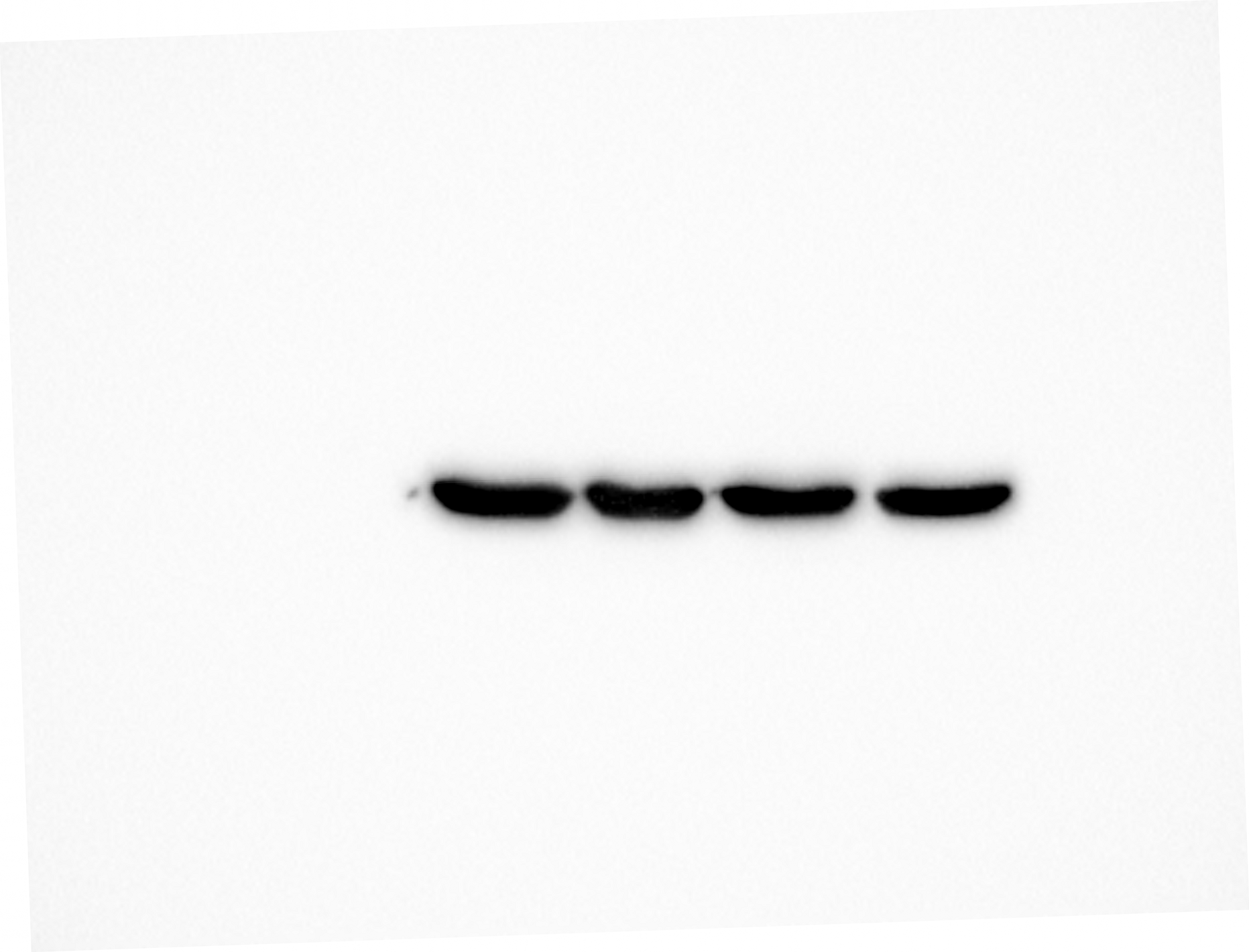

Supplement: Supplementary file 3 [file DataSheet_3.zip › 4F.tif]

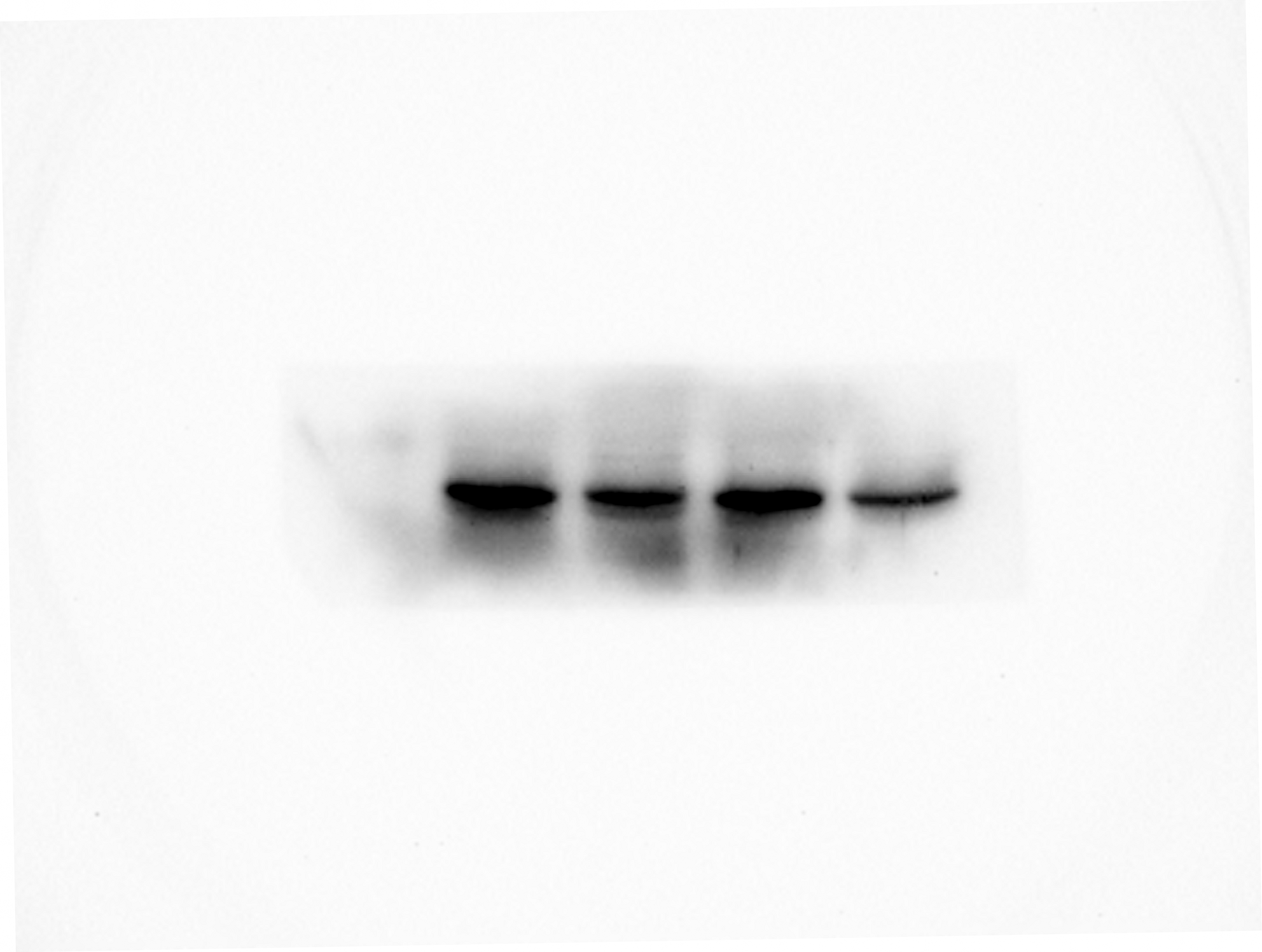

Supplement: Supplementary file 3 [file DataSheet_3.zip › 4G.tif]

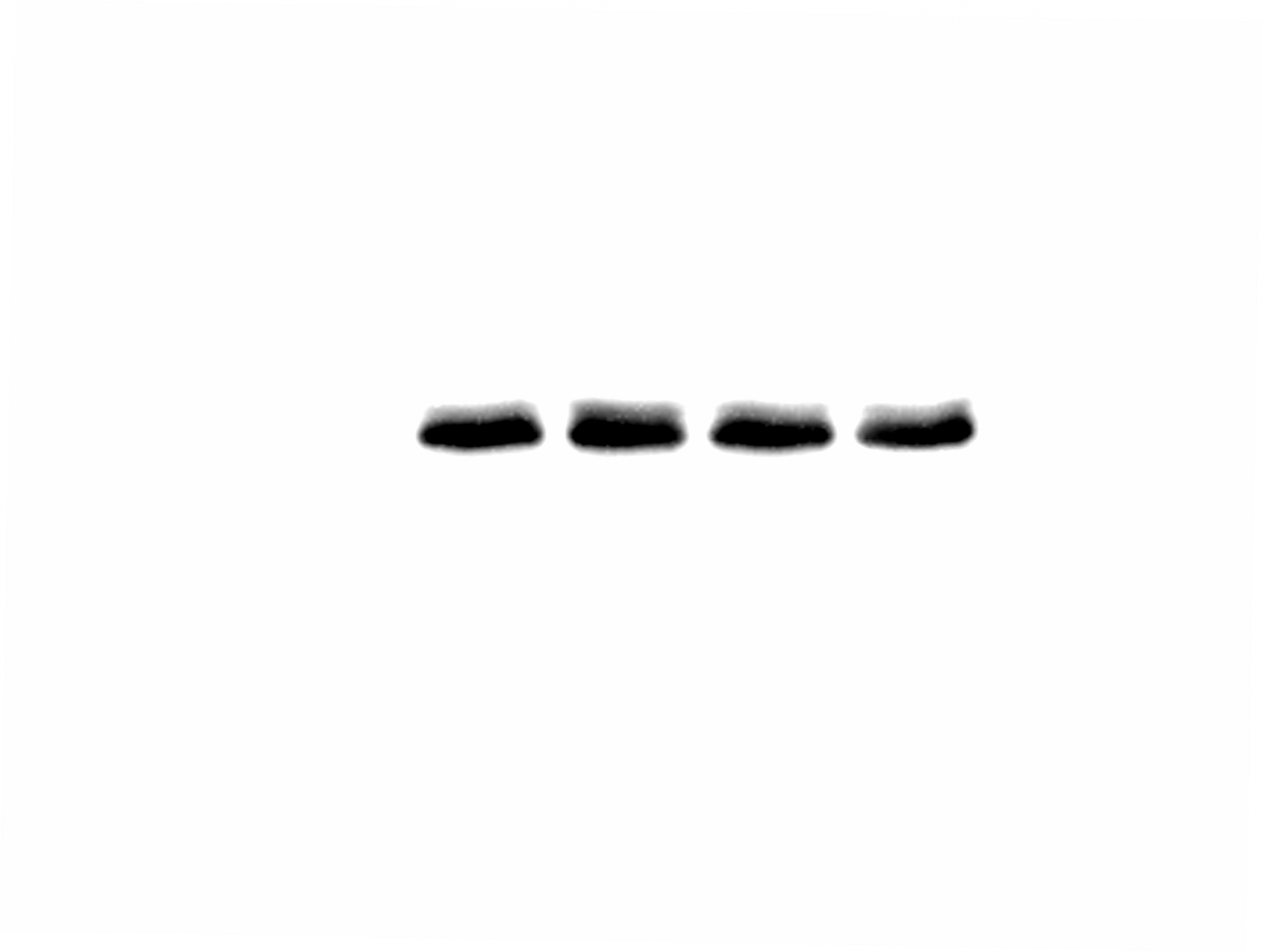

Supplement: Supplementary file 3 [file DataSheet_3.zip › 4H.tif]

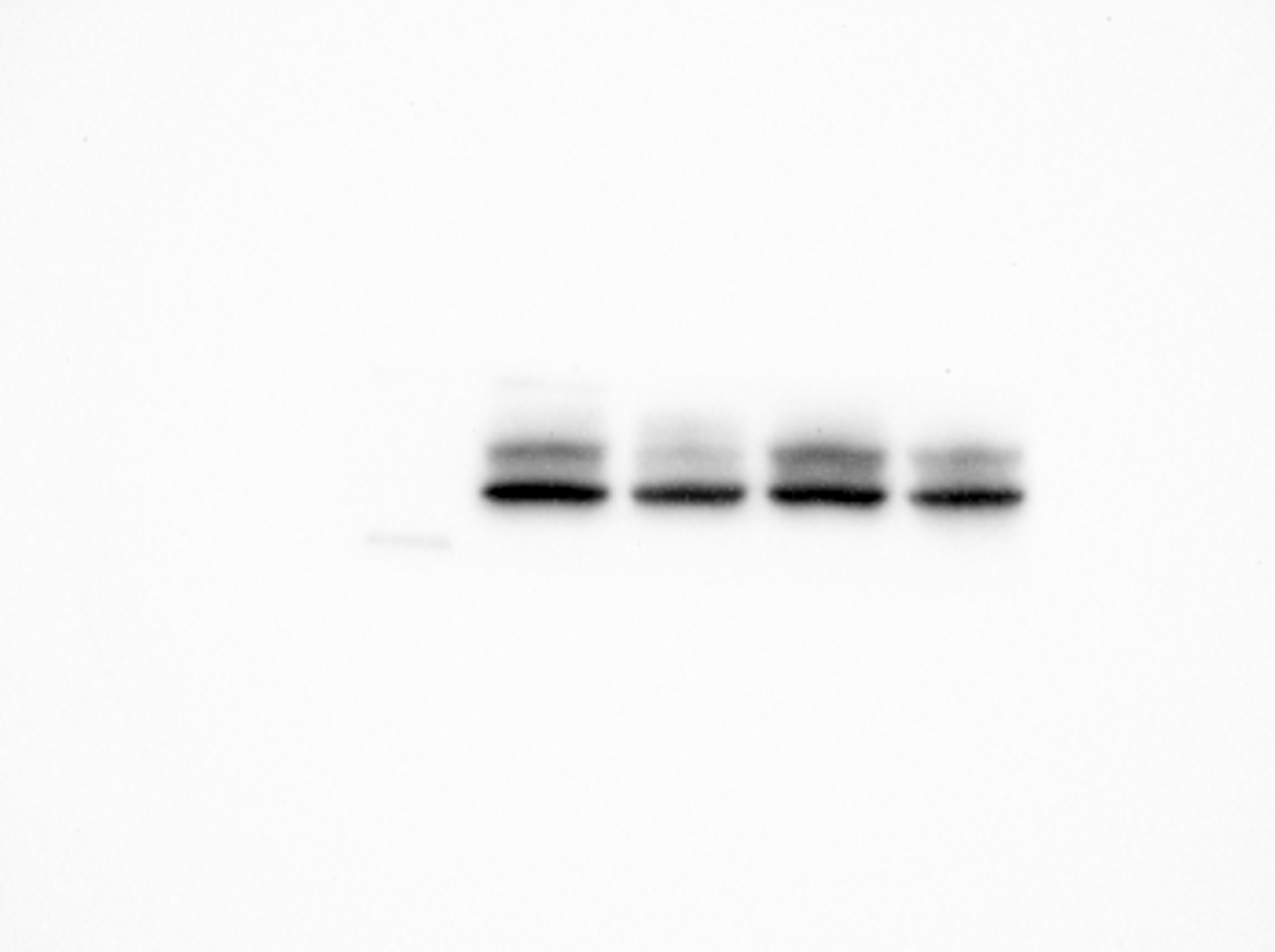

Supplement: Supplementary file 3 [file DataSheet_3.zip › 4I.tif]

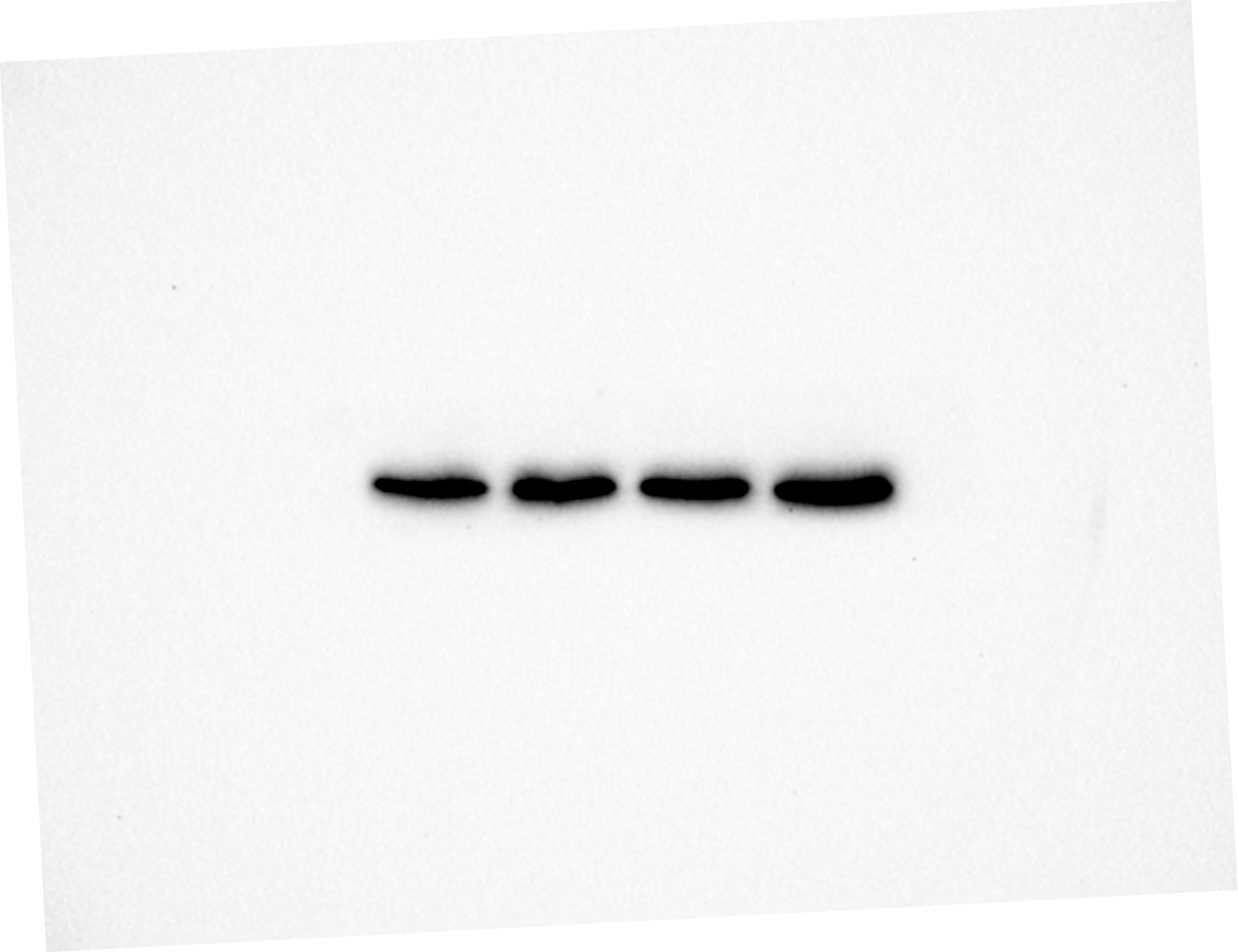

Supplement: Supplementary file 3 [file DataSheet_3.zip › 4J.tif]

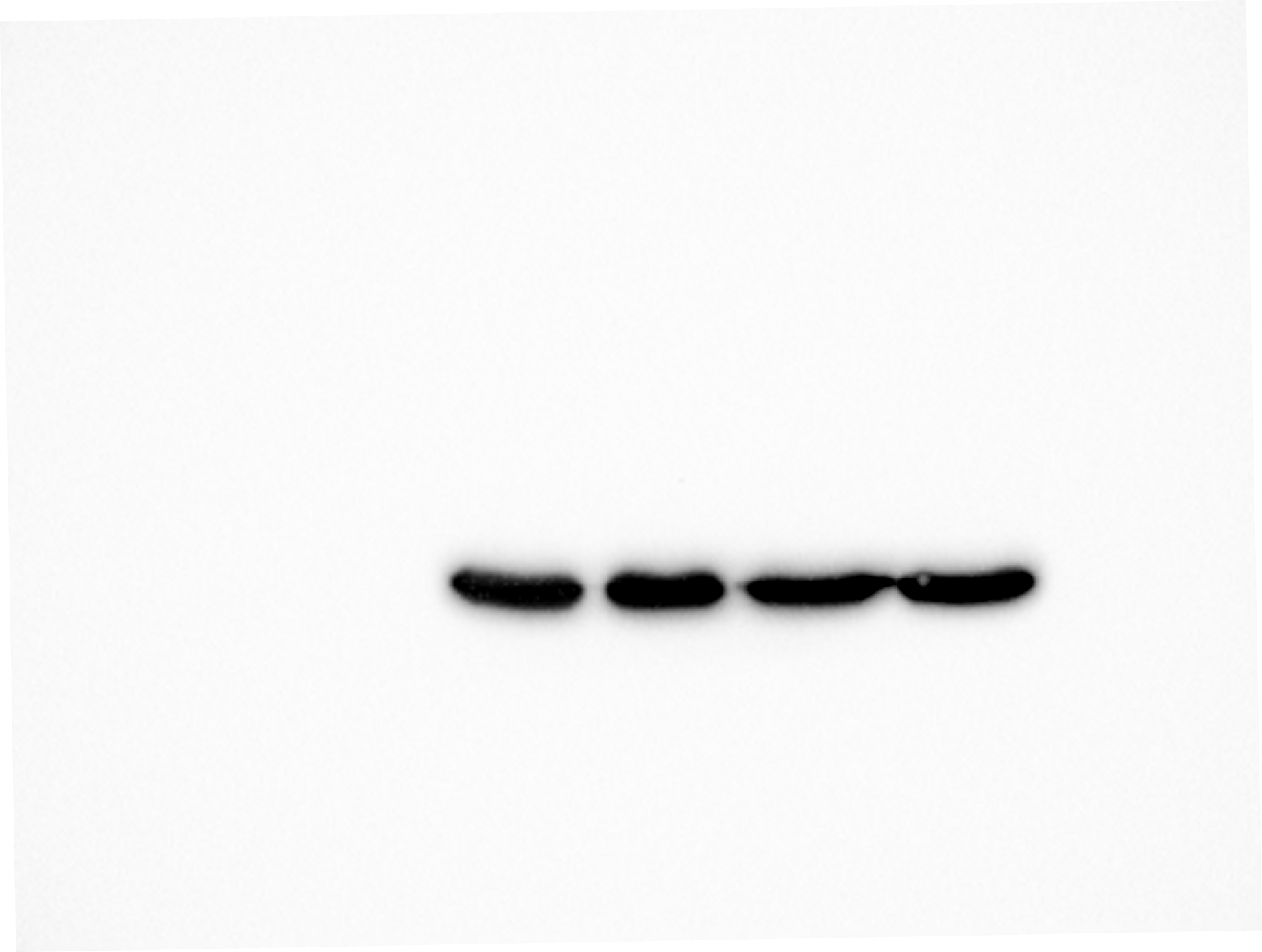

Supplement: Supplementary file 3 [file DataSheet_3.zip › 4K.tif]

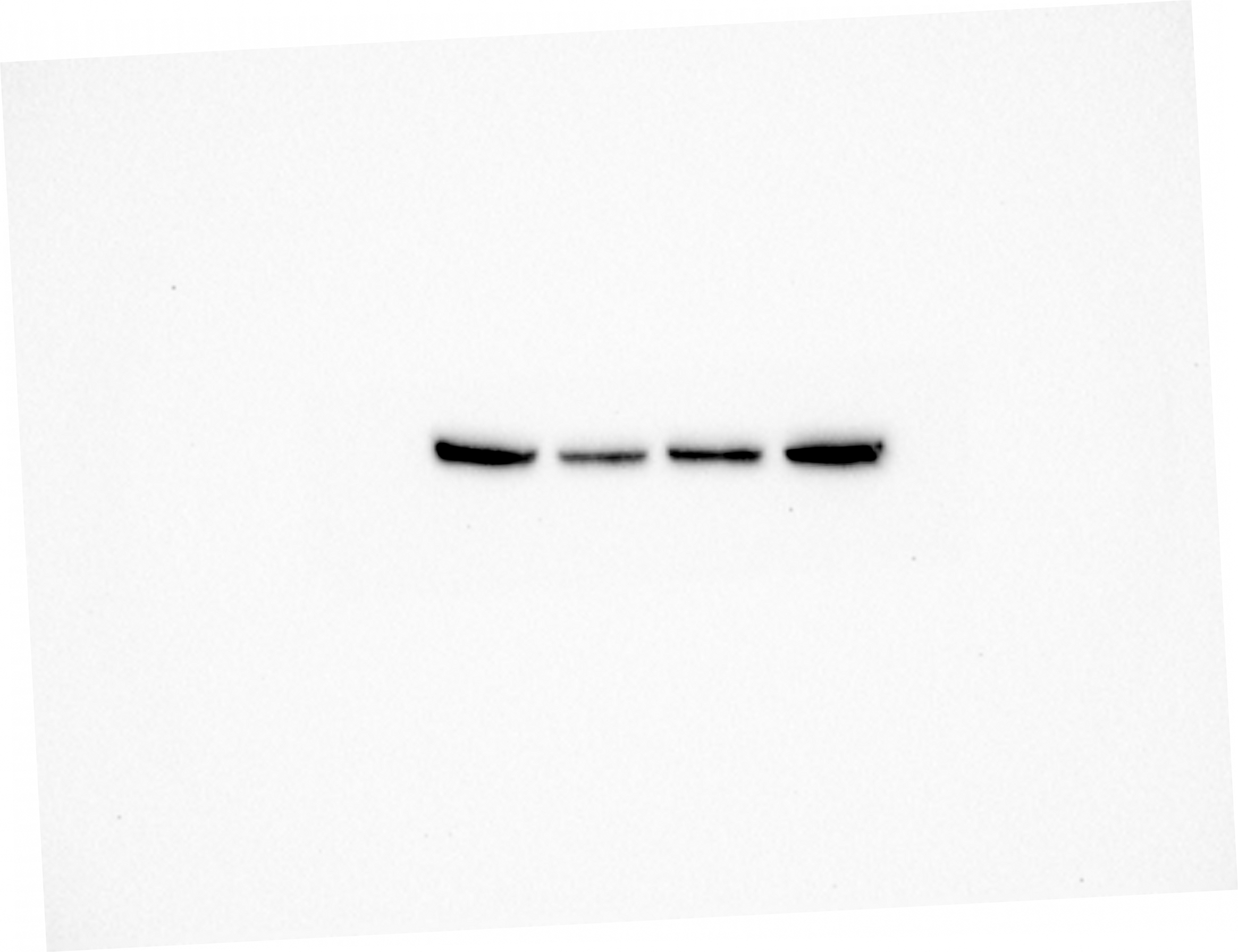

Supplement: Supplementary file 4 [file DataSheet_4.zip › 5A.tif]

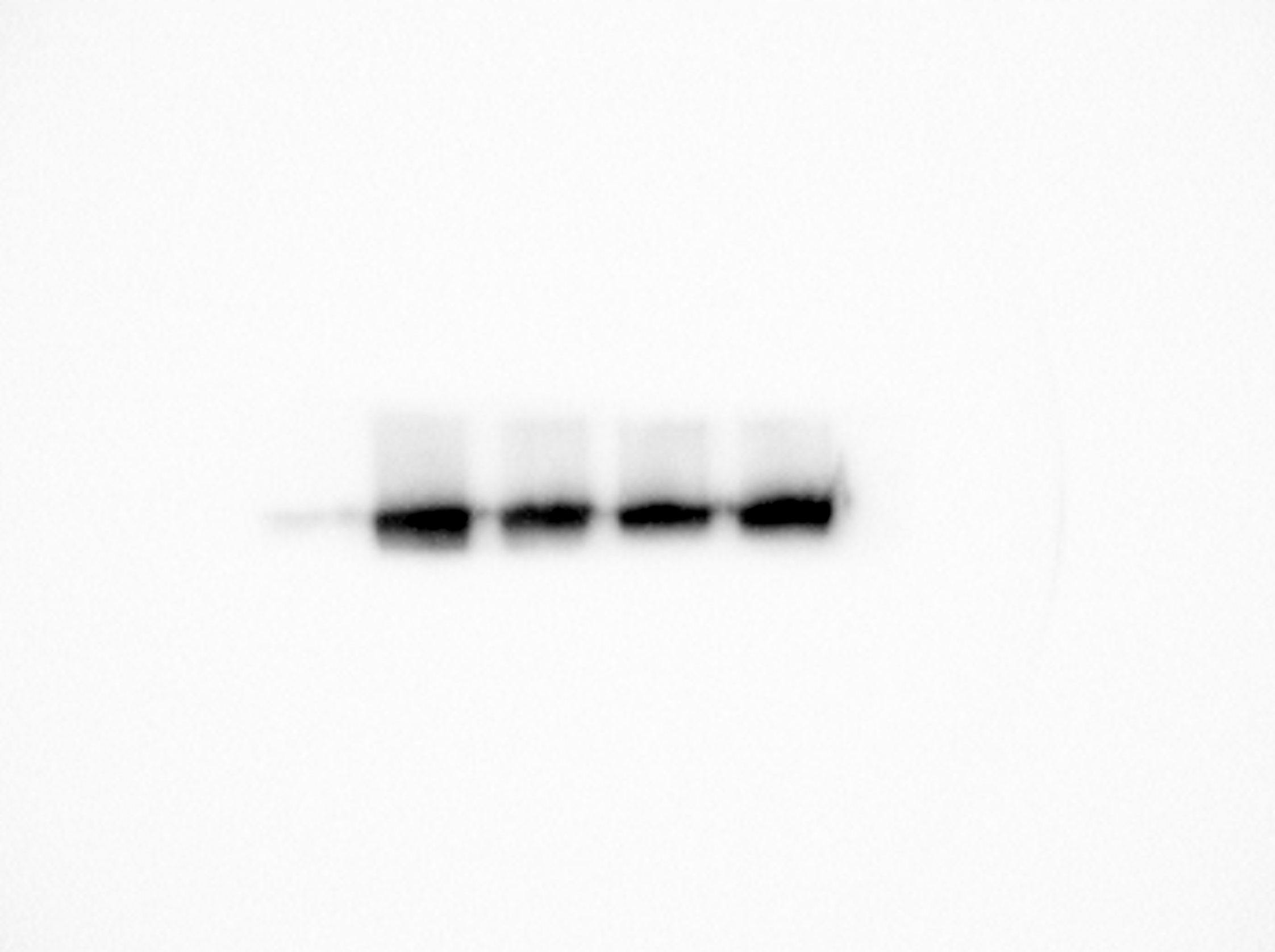

Supplement: Supplementary file 4 [file DataSheet_4.zip › 5B.tif]

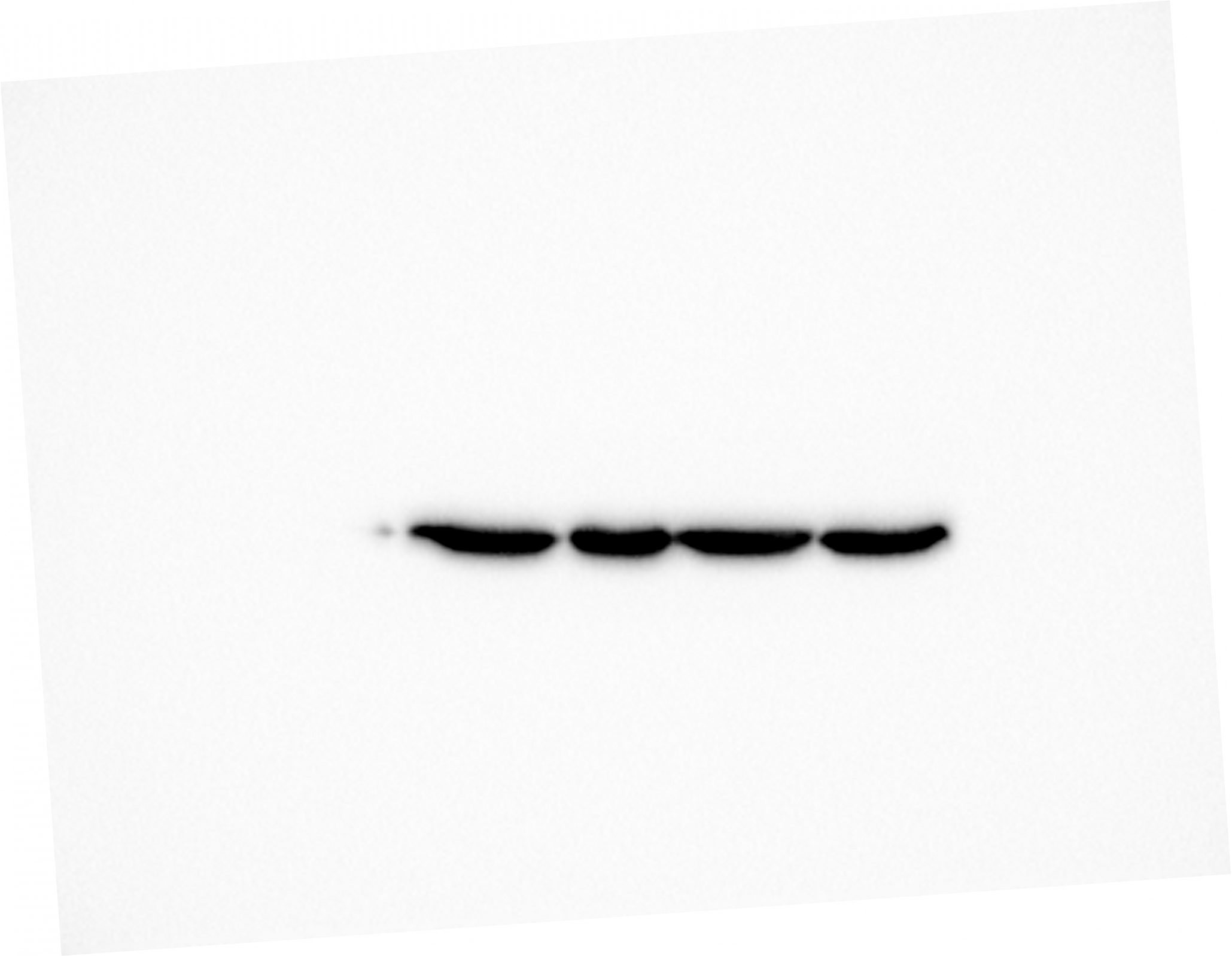

Supplement: Supplementary file 4 [file DataSheet_4.zip › 5C.tif]

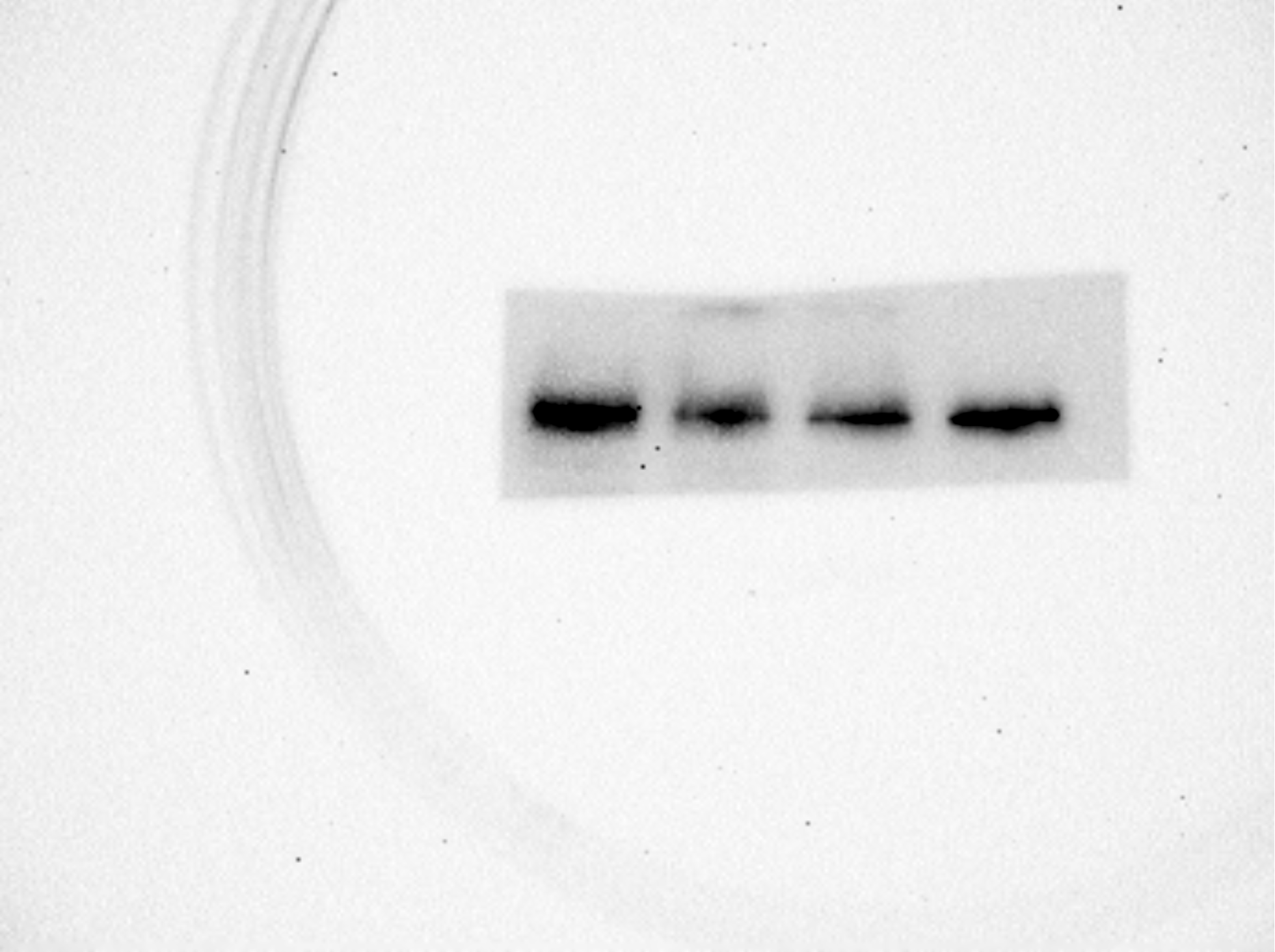

Supplement: Supplementary file 4 [file DataSheet_4.zip › 5D.tif]

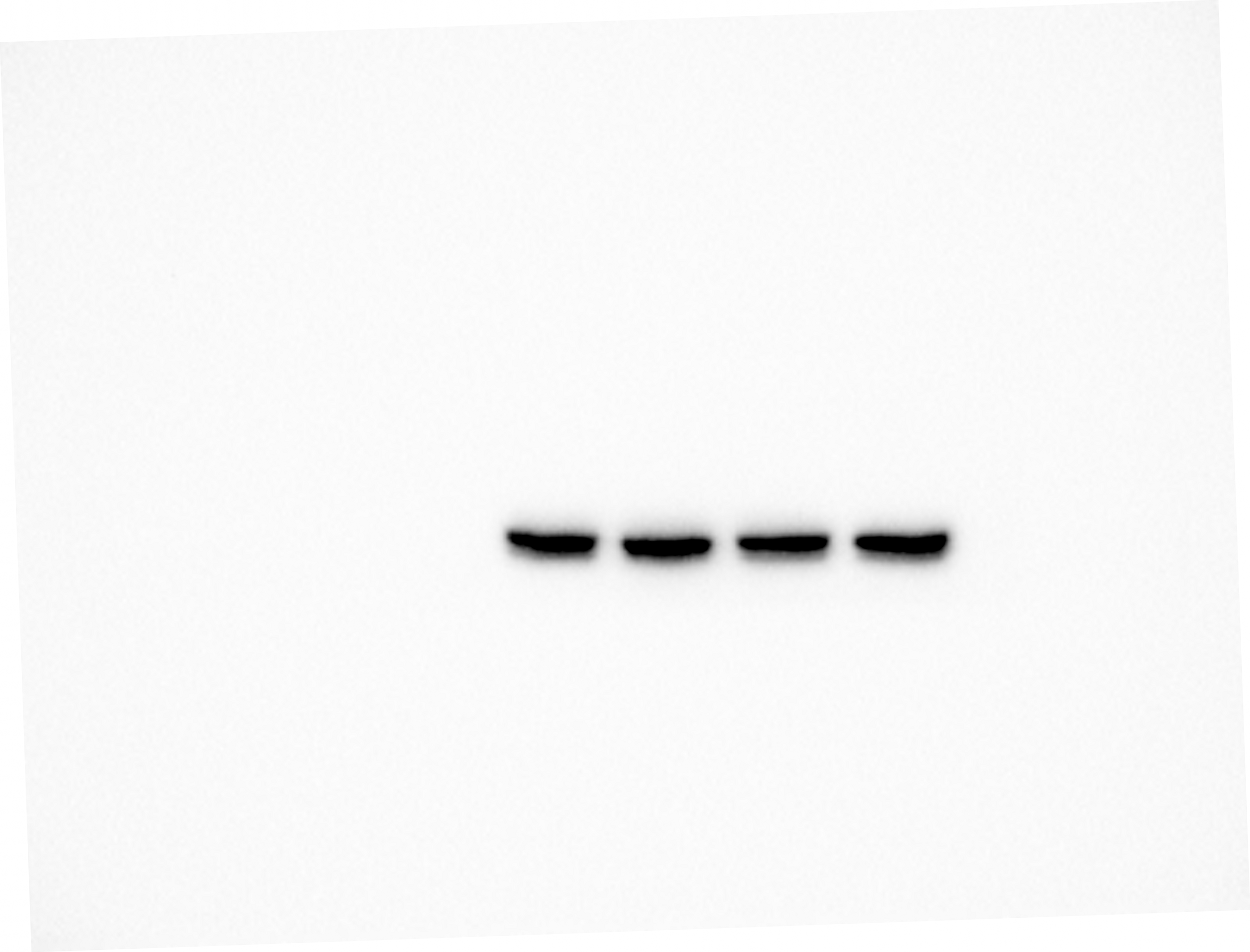

Supplement: Supplementary file 4 [file DataSheet_4.zip › 5E.tif]

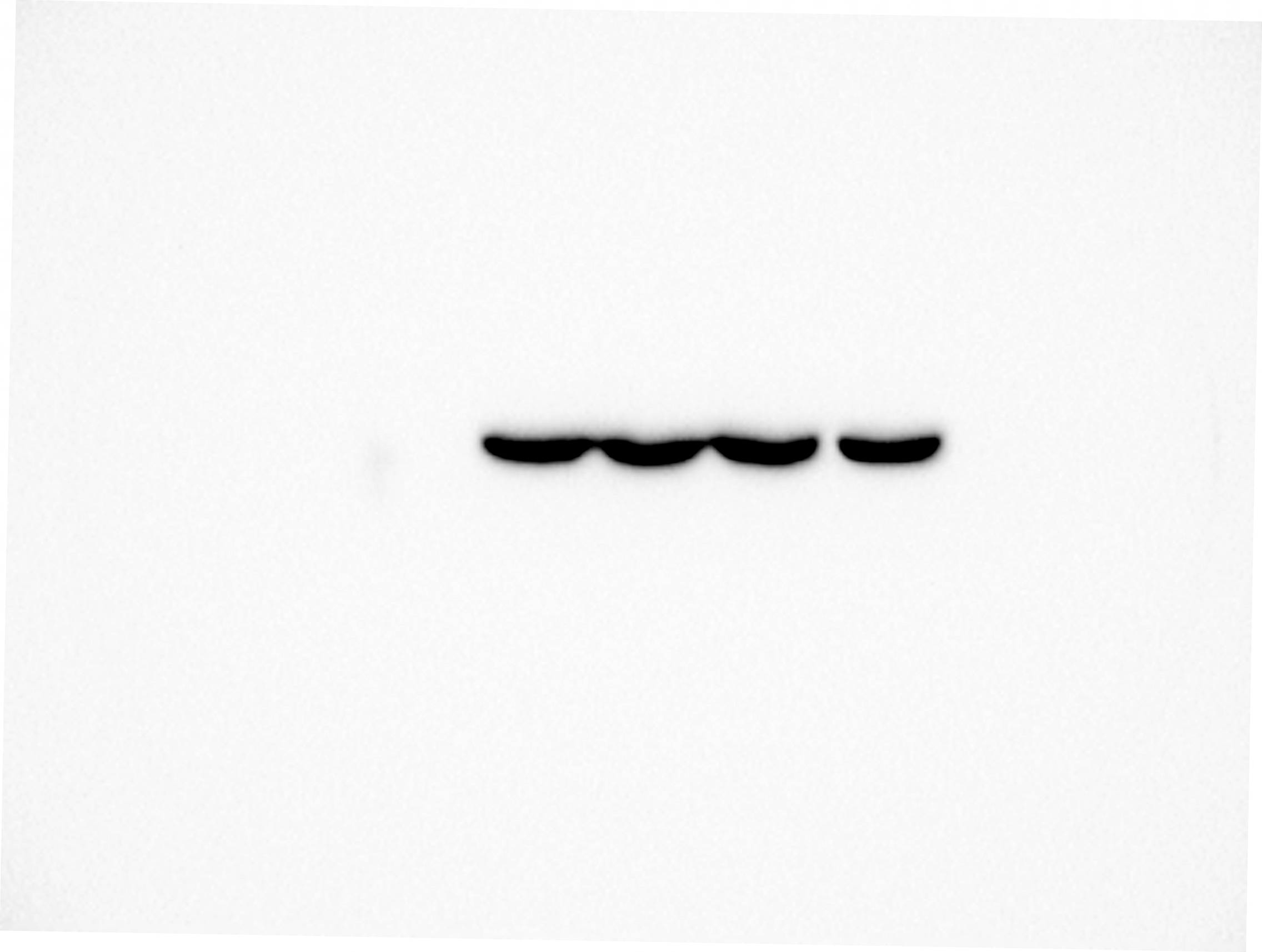

Supplement: Supplementary file 4 [file DataSheet_4.zip › 5F.tif]

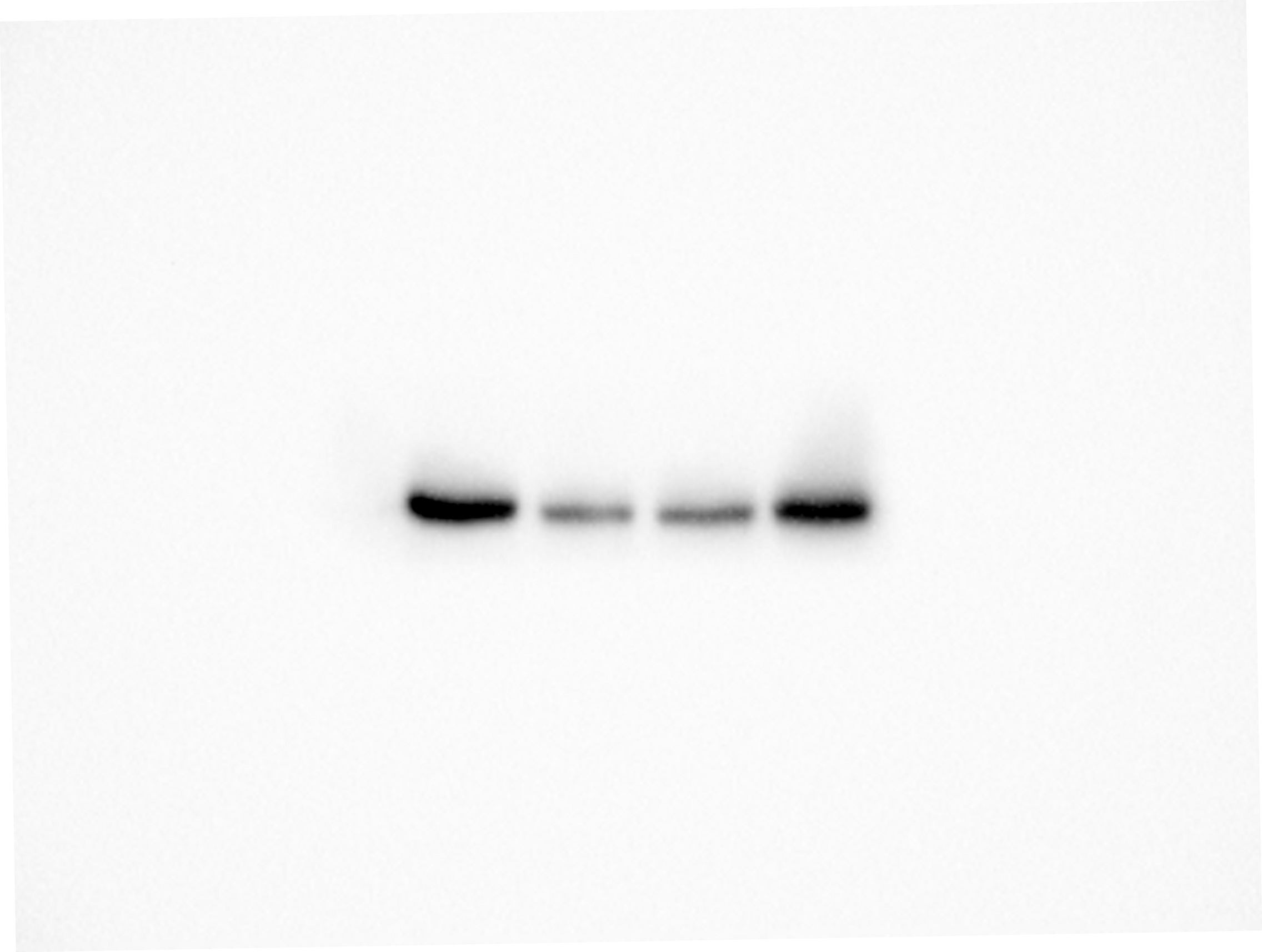

Supplement: Supplementary file 4 [file DataSheet_4.zip › 5G.tif]

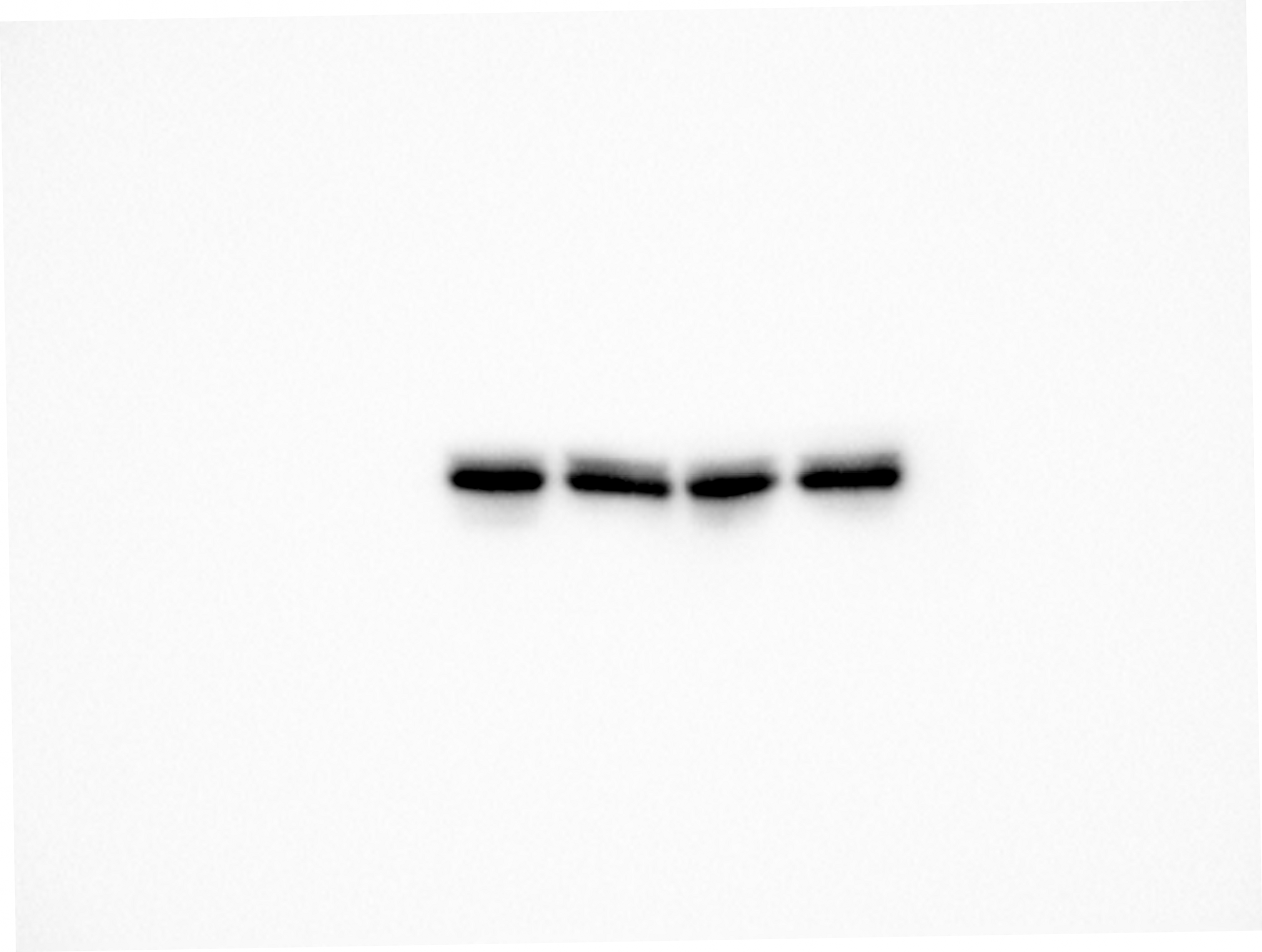

Supplement: Supplementary file 4 [file DataSheet_4.zip › 5H.tif]

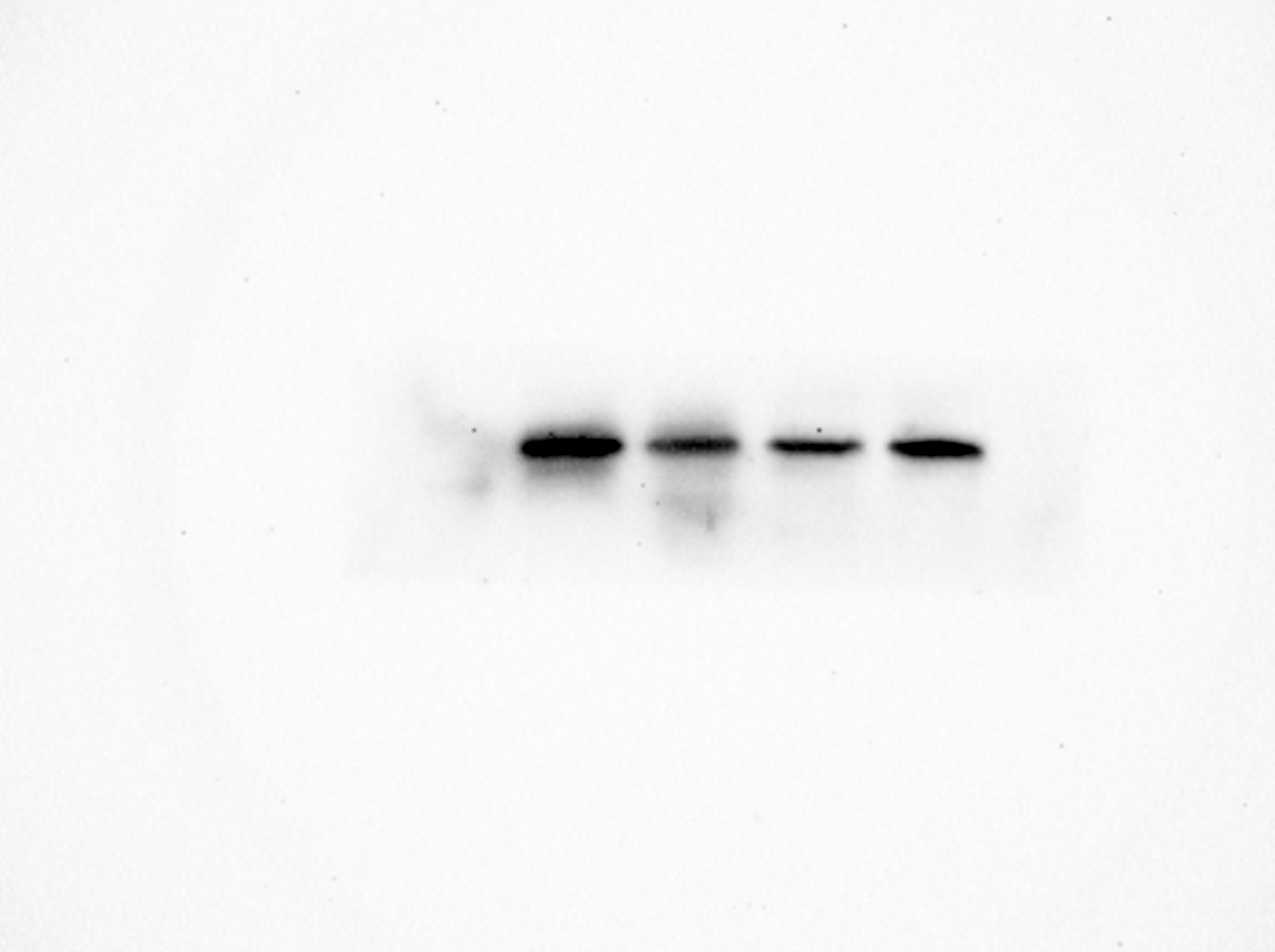

Supplement: Supplementary file 4 [file DataSheet_4.zip › 5I.tif]

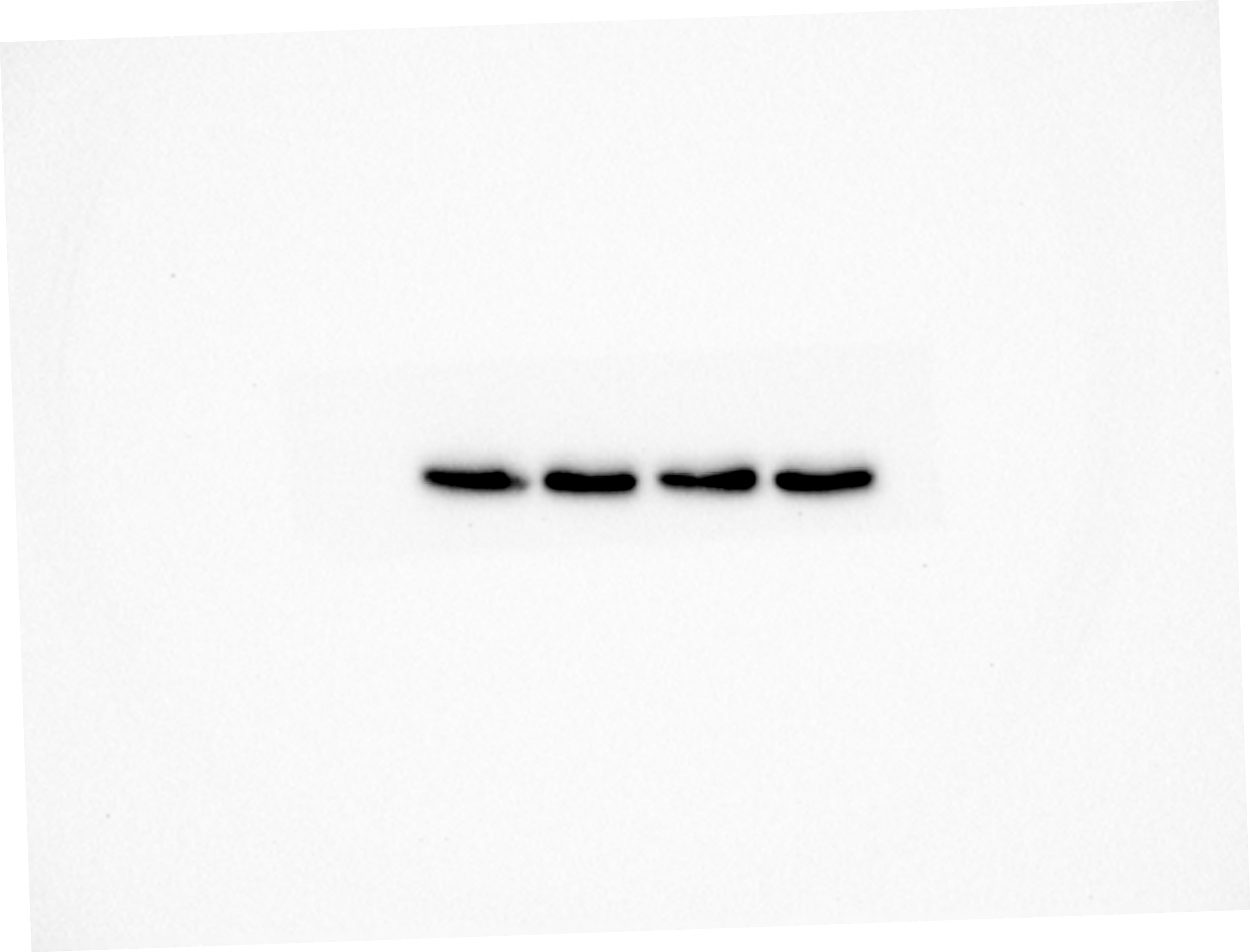

Supplement: Supplementary file 4 [file DataSheet_4.zip › 5J.tif]

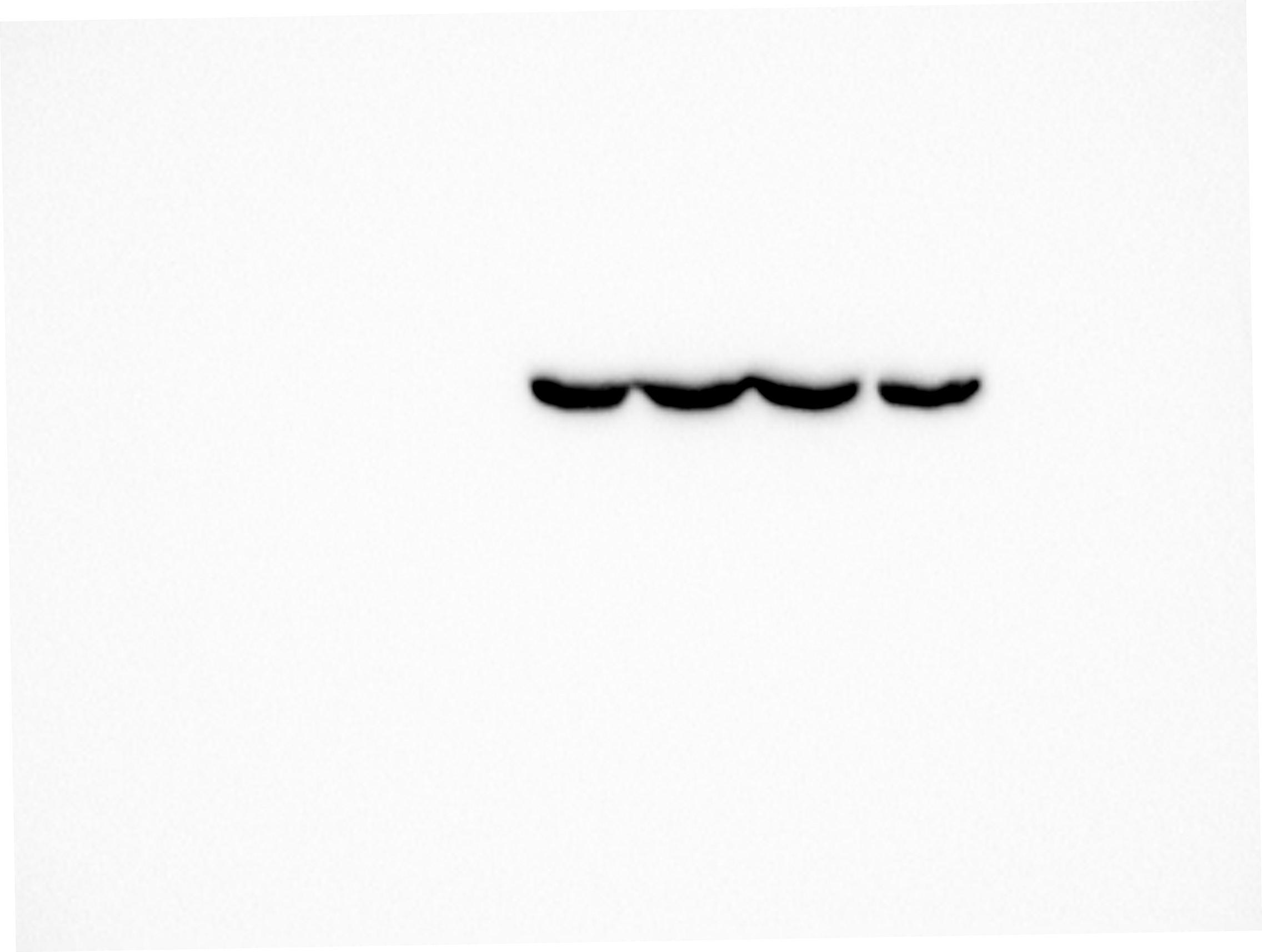

Supplement: Supplementary file 4 [file DataSheet_4.zip › 5K.tif]

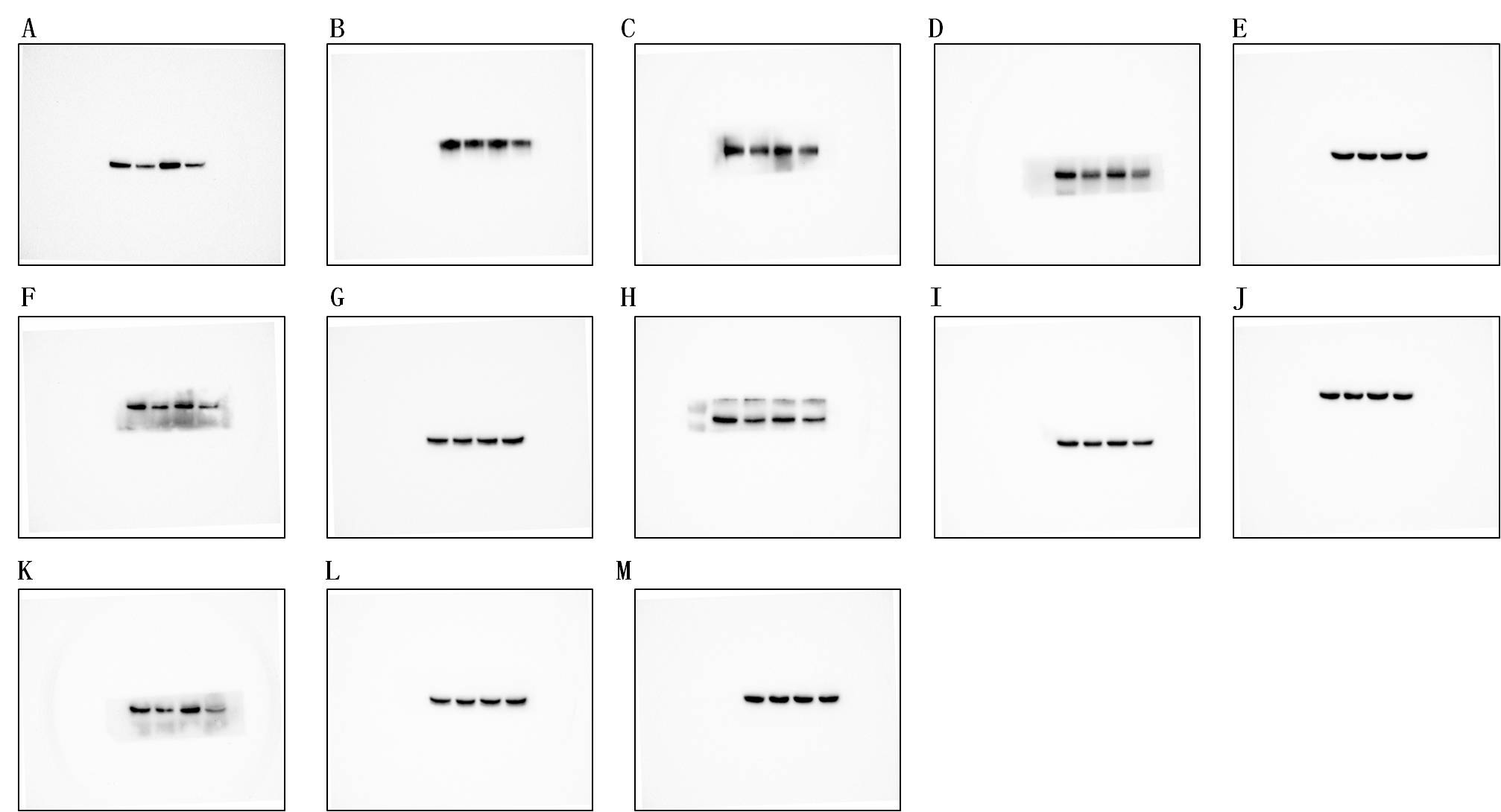

Supplement: Supplementary file 5 [file Image_1.jpeg]

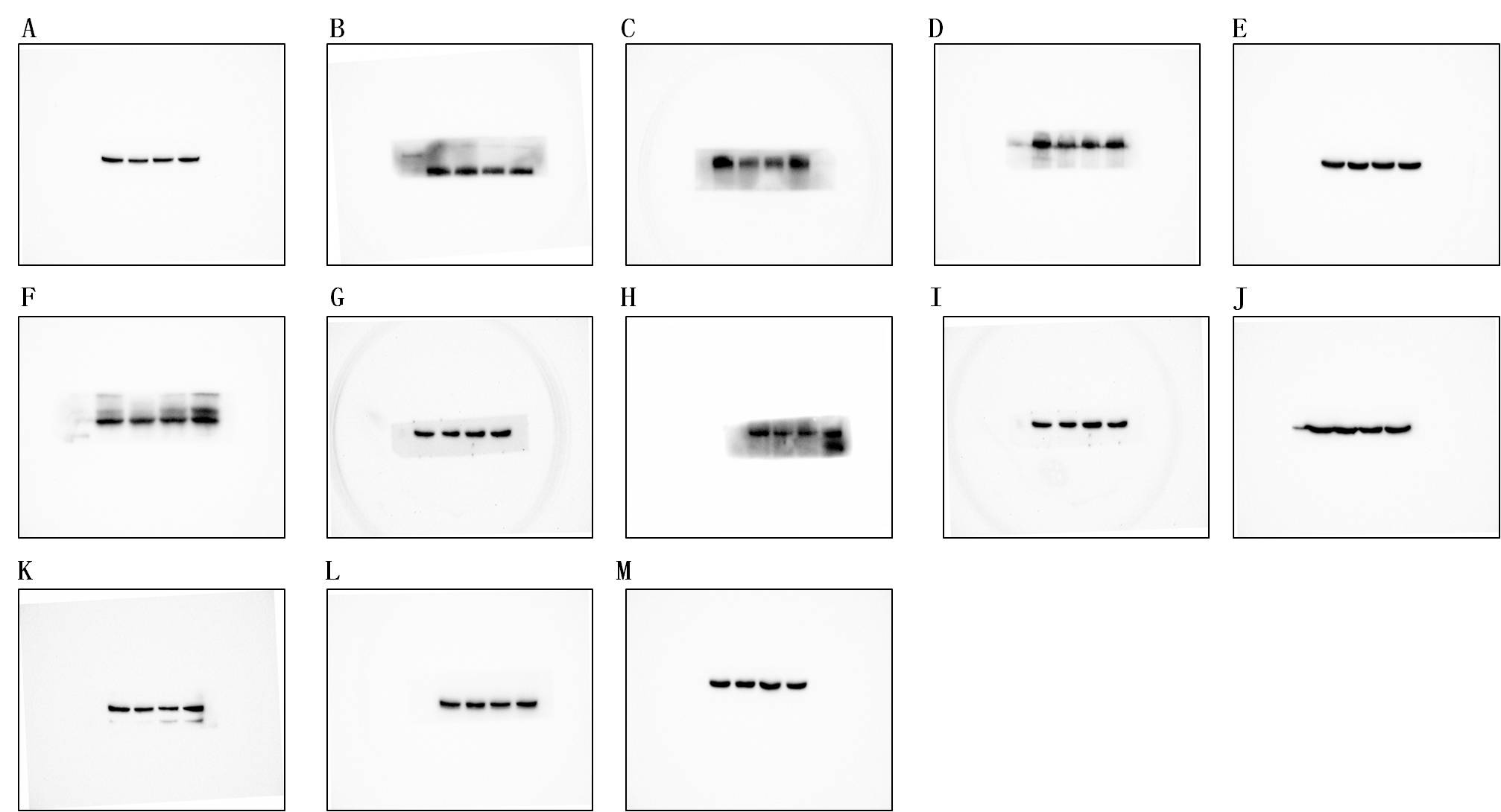

Supplement: Supplementary file 6 [file Image_2.jpeg]

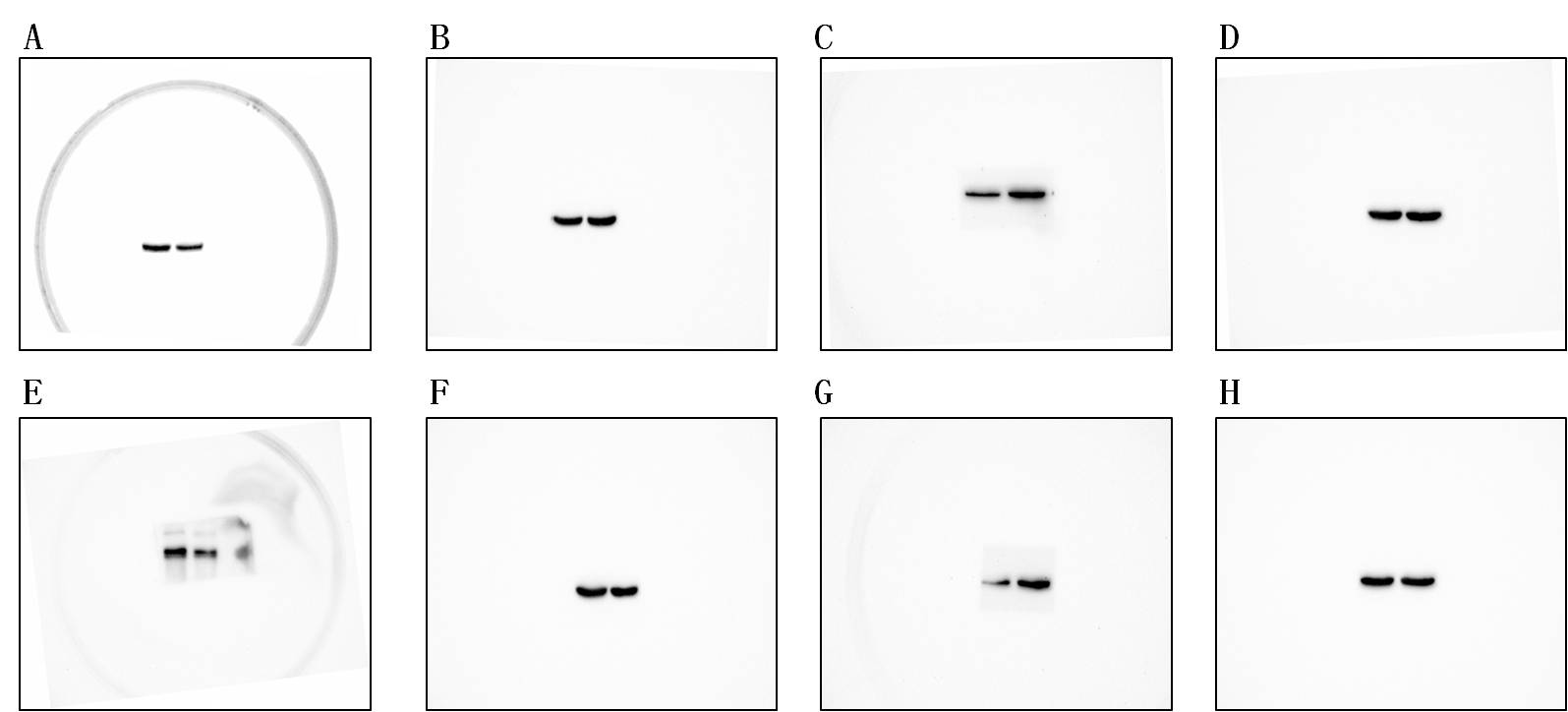

Supplement: Supplementary file 7 [file Image_3.jpeg]

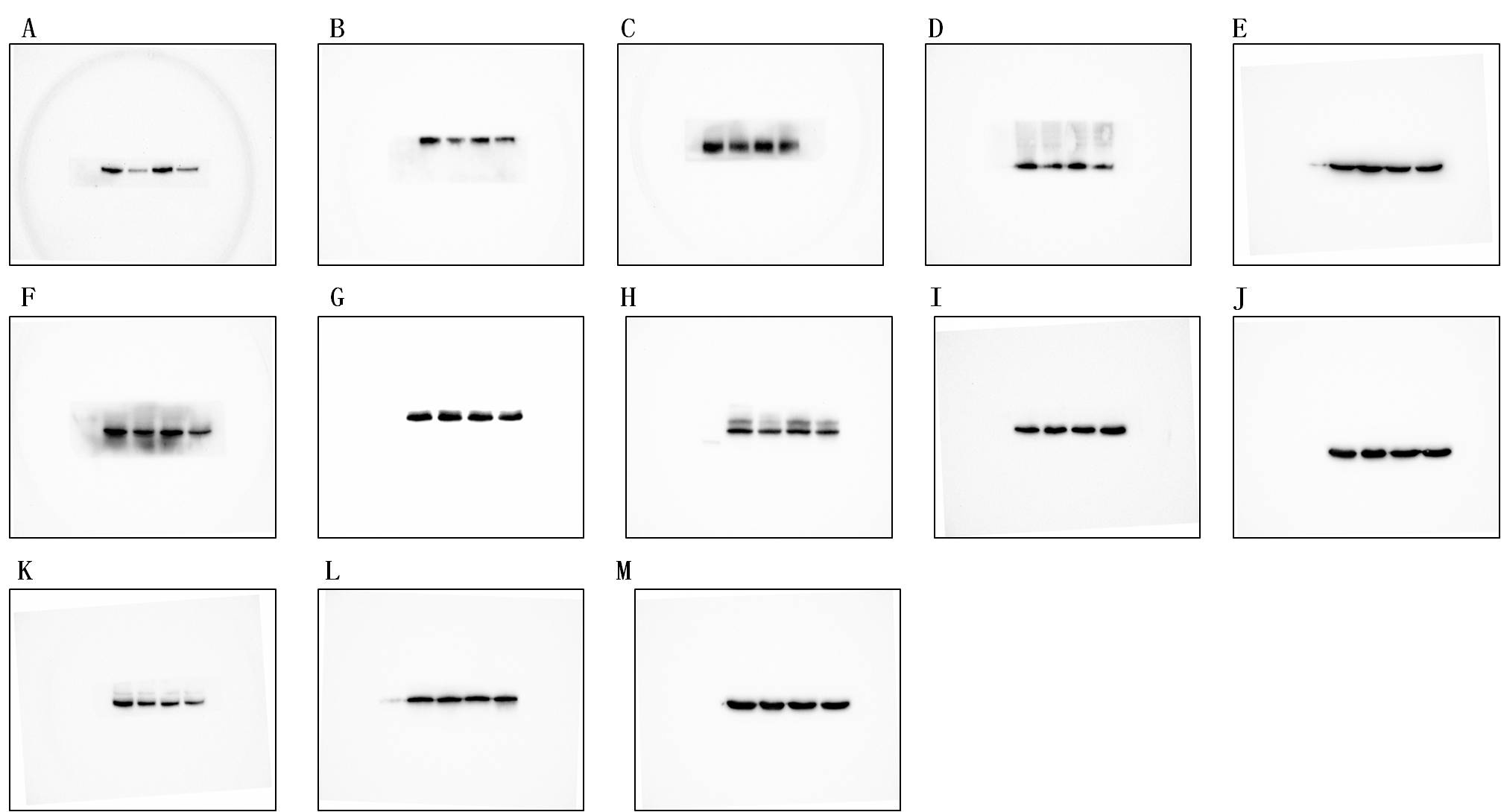

Supplement: Supplementary file 8 [file Image_4.jpeg]

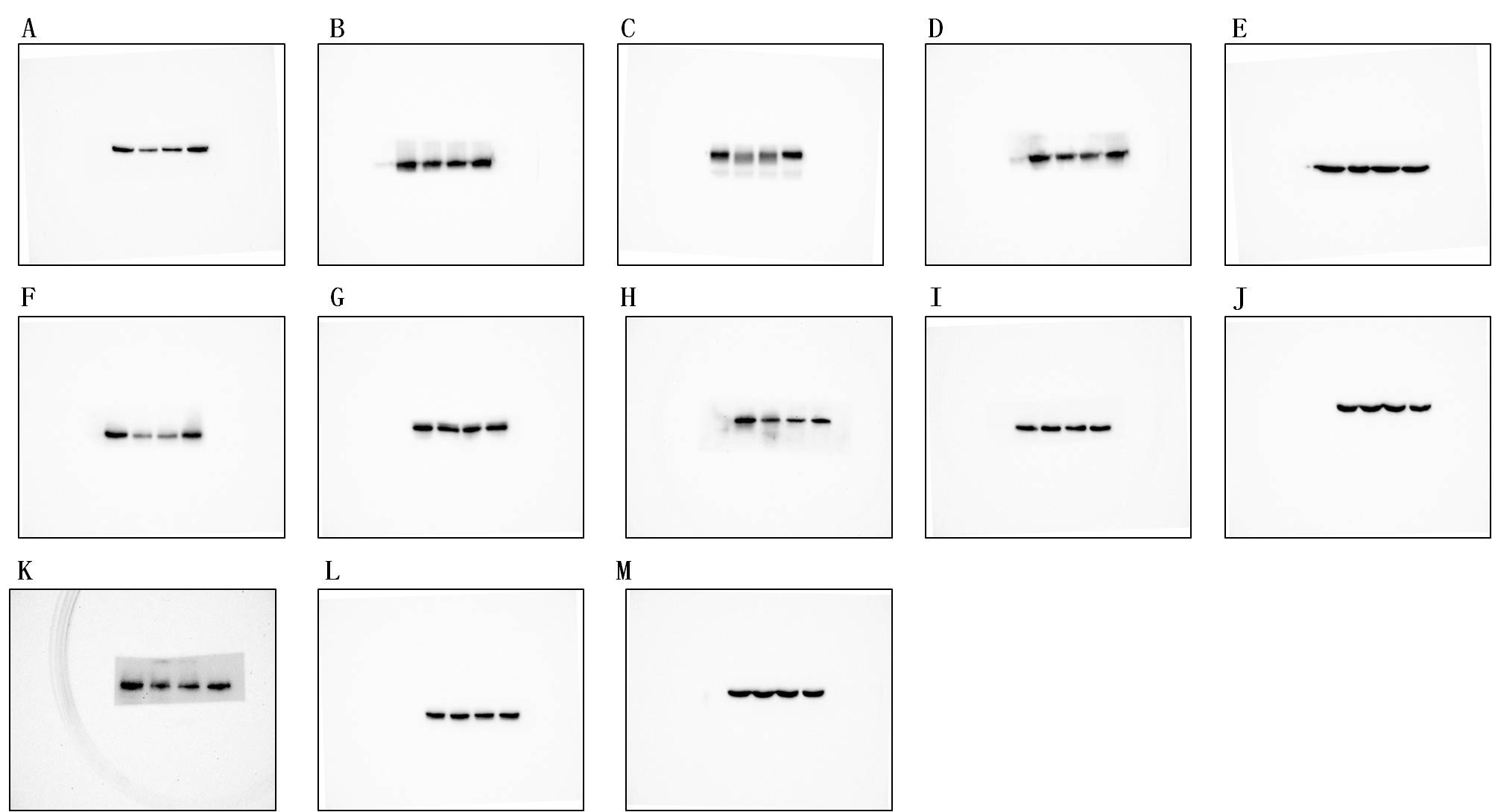

Supplement: Supplementary file 9 [file Image_5.jpeg]
